# Targeted perimetry for mapping the shape of glaucomatous arcuate scotomata

**DOI:** 10.1111/opo.70014

**Published:** 2025-09-08

**Authors:** Ivan Marín-Franch, Brett J. King, William H. Swanson

**Affiliations:** 1 Computational Optometry, Atarfe, Granada Spain; 2 Envision Health Technologies, Brooklyn, New York USA; 3 School of Optometry, Indiana University Bloomington, Bloomington, Indiana USA

**Keywords:** scotomata, static automated perimetry, supra-threshold perimetry, visual field defects

## Abstract

**Purpose:**

Recent work has shown potential benefits for perimetry with dense spacing. To investigate the impact of normal inhomogeneity of perimetric sensitivity on perimetry with dense spacing, suprathreshold perimetry was used near the optic disc where shadows of blood vessels affect sensitivity in healthy eyes.

**Methods:**

Three groups of participants were tested: 58 healthy older controls, 29 healthy younger controls and 18 patients with glaucoma. A Compass perimeter was operated with the Open Perimetry Interface, using custom software with three components: the first found the centre of the blind spot and assessed the general height, the second performed ‘circle perimetry’ with suprathreshold testing at 75 locations on three circles around the disc (radius 6°, 8° and 10°) and the third conducted adaptive ‘high-density perimetry’ to map scotomata. Suprathreshold contrasts were set to a chosen offset above mean normal contrast threshold, adjusted by the general height.

**Results:**

Circle perimetry at different offsets in controls found that an offset of 5 dB was needed to keep the false positive rate below 5%. Test–retest measurements in controls found limits of agreement for general height were ±2.5 dB, and that reproducible defects on circle perimetry were at locations consistent with shadows of blood vessels. Circle perimetry and high-density perimetry with a small offset of 1 dB led to the appearance of artefactual arcuate defects in controls. Circle perimetry with an offset of 5 dB in 26 eyes of 18 patients with glaucoma found scotomata in 22 sectors of 16 eyes in 13 patients. High-density perimetry was performed with a 5 dB offset for 15 sectors in 12 of these eyes and confirmed arcuate defects in all cases.

**Conclusions:**

Targeted perimetry identified arcuate perimetric defects but required an offset of 5 dB to have reasonable specificity. Suggestions are made for improving the performance of targeted perimetry.

**Supplementary Information:**

The online version of this article (doi:10.1111/opo.70014) contains supplementary material, which is available to authorized users.

## Key points

- The ability to delineate shapes of visual defects has potential for structure–function comparisons and assessing progression in terms of an increase in the area of such defects.
- Customised locations for suprathreshold perimetric testing near the blind spot made it possible to quickly delineate the shapes of visual field defects.
- Small depressions of sensitivity near the optic disc due to large blood vessels required sufficiently large suprathreshold contrasts in order to avoid false positive results in healthy eyes.

## INTRODUCTION

Static automated perimetry is used clinically to detect glaucomatous visual field defects by measuring luminance increment thresholds and comparing them to values in a normative database. The approach of measuring thresholds requires either using relatively coarse spacing, such as the 24-2 grid with 6° spacing, or limiting the size of the region tested such as the 10-2 grid with 2° spacing. Coarse spacing can miss localised defects in patients with early glaucoma,^1–6^ as can limiting the size of the region tested.^7^ These factors can also reduce the ability to detect progression.^8^ Suprathreshold testing has potential to address this problem, by allowing a larger number of test locations.^9–13^

One approach for finer spacing is to choose alternative locations for different eyes, such as where defects are identified on retinal imaging.^2^ In the absence of information from retinal imaging, locations may be chosen based on perimetric asymmetry across the horizontal meridian,^4,14^ or using results from an initial grid of locations and iteratively choosing additional locations based on responses at prior locations in order to delineate shapes of glaucomatous scotomata.^15^ The current study further explores the approach of delineating scotomata with suprathreshold testing; in this case using initial locations near the optic disc and then assessing the shapes of scotomata that extend from the disc. The motivation is the finding that thin retinal nerve fibre layer (RNFL) wedge defects can reveal corresponding arcuate perimetric defects that fall between the locations in the 24–2 grid and connect to the physiological blind spot.^16^ The goal is to identify challenges in delineating scotomata with suprathreshold testing, in order to provide guidance for future studies working towards clinically useful tools for using denser spacing in perimetric testing.

A challenge in the use of denser spacing of perimetric locations is that healthy eyes can have local inhomogeneities in perimetric sensitivity due to the shadows of the blood vessels causing local regions with depressed sensitivity, called angioscotomata.^17–20^ Angioscotomata can occur across the retina and can be readily studied near the optic disc.^19,21^ Therefore, four studies were performed near the optic disc. All used suprathreshold perimetry at a set of fixed locations targeting the region around the disc, and two followed this by adaptively selecting locations along expected paths of nerve fibre bundles. The first three studies evaluated factors affecting specificity by performing suprathreshold perimetry in 87 healthy controls free of eye disease. Stimulus contrasts were defined in terms of offsets from age norms adjusted by the ‘general height’ for an individual, measured by another brief protocol. The general height accounts for factors such as differences across devices (how ‘decibels’ are calibrated and optical differences among perimeters), effects of light scatter (due to lens ageing) and psychophysical factors (an individual's criterion and attentiveness).^22^ Suprathreshold stimuli were presented at 75 fixed locations on three circles of 25 locations each, centred on the blind spot. The first study evaluated the effect of offset while the second evaluated test–retest variability both for general height and a single offset. The third study assessed the effect of false positives for adaptive location selection by using an offset small enough that a number of stimulus presentations would not be seen. The fourth study tested patients with glaucomatous perimetric defects, using an offset that had a low false positive rate to determine how often adaptive location selection confirmed arcuate defects detected with the fixed locations. The findings provide guidance for further studies of perimetry with dense locations.

## METHODS

### Participants

Four groups of participants were recruited from patients at the Atwater Eye Care Center at Indiana University School of Optometry. Two older control groups were recruited for the first two studies: a group of 28 people, aged 44 to 88 years, median 62 years and interquartile range (IQR) 55 to 72 years and a group of 30 people, aged 41 to 82 years, median 62 years and IQR 51 to 68 years. A group of 29 younger controls was recruited for the third study: aged 20 to 27 years, median 23 years and IQR 22 to 24 years. A group of 18 patients with glaucoma was also recruited, aged 62 to 87 years, median 76 years and IQR 70 to 80 years.

Details of inclusion and exclusion criteria have been published elsewhere.^16,23^ Participants were required to have clear ocular media and corrected monocular distance visual acuity of at least 6/12 at the study visit; in fact, all participants had visual acuity of 6/7.5 or better. Participants were required to have a spherical equivalent refractive correction between −12 dioptres (D) and+3 D and cylindrical correction <3.0 D. High refractive error can be a problem with bowl perimeters because corrective lenses can cause magnification or minification, but the Compass perimeter (icare-world.com) focuses on the retina and thus does not require corrective lenses. Myopic refractive error can reduce the measured circumpapillary RNFL thickness, so norms can require spherical error to not be worse than −6 D; for the patients with glaucoma, the spherical equivalent refractive error was in the range +1.6 to −5.5 D, which is within the range for the normative database.

Participants were required to be under the care of an eye care practitioner and to have had a recent clinical examination with normal retinal findings (except for those associated with glaucoma in the diagnosed patients); patients with glaucoma were also required to have glaucomatous perimetric defects. A glaucomatous visual field was defined as having a reproducible defect (in at least two consecutive reliable visual fields) of either two or more contiguous points with *p* < 0.01 loss or greater, three or more contiguous points with *p* < 0.05 loss or greater, or a 10-dB difference across the nasal horizontal midline at two or more adjacent points in the total deviation plot. At least one eye had to meet these criteria in order for the patient to be diagnosed with glaucoma. For individuals in which only one eye met the criteria for a glaucomatous visual field, after that eye was tested the fellow eye was examined if there was sufficient time (this ended up being just two patients). For the participants with glaucoma, perimetric results with 24-2 or 10-2 test patterns on the Zeiss Humphrey Field Analyzer (zeiss.com) were collected from clinic records with the patient's written permission, and circumpapillary RNFL thickness was measured during the study visit with a Heidelberg Spectralis optical coherence tomograph (heidelbergengineering.com). Circumpapillary RNFL thickness was used to assess RNFL damage because testing was conducted near the optic disc and a quantitative evaluation was desired.

The research for this study adhered to the tenets of the Declaration of Helsinki and was approved by the Institutional Review Board at Indiana University. Written informed consent was obtained from each participant after an explanation of the procedures and goals of the study, before the testing began. The test results analysed here, along with a full list of ages and refractive errors for all participants, are given in Appendix S1. Graphs of 24-2 visual fields for all 26 tested eyes of patients with glaucoma are provided in Appendix S1.

### Equipment

Testing was performed with an iCare Compass automated perimeter (icare-world.com) driven through the Open Perimetry Interface (OPI), which is described elsewhere.^24–26^ The Compass has a background luminance of 10 cd m^−2^ and defines stimulus contrast in units of ‘dB’ in the same manner as the Humphrey Field Analyzer: 100% Weber contrast is called ‘25 dB’ and a change by 1 dB represents a 0.1 log unit change in contrast. The Compass has active fundus-based retinal tracking at 25 Hz and its software actively repositions the stimulus to compensate for shifts from the fixation point in order to deliver the visual stimuli as close as possible to the intended retinal region.^27^ When the integrated system loses track of the retina, the test is paused until the retina is tracked again. The experiments conducted here were run with a custom app written in R (r-project.org)^28^ with the Shiny package^29^ along with the OPI and the normative values from the visualFields package.^25,26^ The app is freely available in the public GitHub repository https://github.com/imarinfr/mapPerim.

### Algorithms

An algorithm to locate the blind spot and three algorithms developed as part of this work (general height estimation, circle perimetry and high-density perimetry) were run in order for the experimental protocols described in the next subsection. Thus, the location of the centre of the physiological blind spot was first estimated, followed by the general height and then circle perimetry. For two protocols, if circle perimetry found a region of reduced sensitivity, then a fourth algorithm, high-density perimetry, was used to try and delineate an arcuate region with reduced sensitivity.

#### Locating the blind spot

The centre of the blind spot was identified using a series of presentations at a suprathreshold stimulus contrast of 17 dB (631% Weber contrast). The stimulus diameter was 0.43° for this and all algorithms. The algorithm first found the horizontal segment spanning the blind spot, calculated its midpoint, used that as the horizontal position for the centre of the blind spot and tested a vertical segment spanning the blind spot at that horizontal location. The midpoint of the vertical segment was used as the estimated vertical position of the centre of the blind spot. In the first part, to obtain the horizontal segment, the algorithm presented stimuli sequentially from 6° (for a right eye) or −6° (for a left eye) at the vertical position of *y* = −3°, and then moved temporally in 2° steps until the participant did not respond to a stimulus presentation. Then it was moved nasally in horizontal 1° steps until the participant responded to the stimulus and back again in steps of 1° temporally until the stimulus was seen again. The edges of the horizontal segment were the midpoints between seen and not-seen locations at both sides of the blind spot. The vertical segment was obtained by moving the stimulus at the estimated horizontal location of the blind spot, moving vertically from −18° in 3° steps superiorly until the participant did not respond to a stimulus presentation. Then it moved inferiorly in steps of 1° until the participant responded to the stimulus and then moved in steps of 1° superiorly until the stimulus was seen again. The edges of the vertical segment were the midpoints between seen and not-seen locations at both sides of the blind spot.

#### Estimating the general height (GH)

The GH, mathematically defined as the difference between the overall sensitivity of the visual field and the age-corrected mean reference field, is typically estimated as the 85th percentile of the deviations^30^ from the reference field sensitivities. In conventional perimetry, this post hoc calculation is used to compute the pattern deviations (PD) and their corresponding probability maps.^31^ Here, the estimate was based, instead, on a series of direct measurements from an algorithm whose purpose was to quantify the GH using a simple implementation of the family of ZEST algorithms^24,32^ that presented 12 visual stimuli at eight locations, 6° away from the centre of the blind spot. In this ZEST implementation, the prior distribution was a Gaussian centred at 25 dB with a standard deviation of 6 dB. Visual stimuli were presented (in polar coordinates) at angles of 0°, 45°, 90°, 135°, 180°, 225°, 270° and 315° from the blind spot. It was assumed that perimetric sensitivity would be roughly uniform around this circle,^21^ and the ZEST algorithm was applied treating the subject's responses from all eight locations indistinguishably. The first eight stimuli were presented in order at 0°, then 180°, 90°, 270°, 45°, 135°, 225° and 315°. To reduce the chance of damaged locations affecting the estimate of the GH, the four locations for which the participant responded to the dimmest stimuli were selected. Or if in the previous step fewer than four stimuli were seen, then all seen locations were selected first followed by the locations where the dimmest stimuli were presented, up to four. The ZEST algorithm then continued presenting four more stimuli once at each of these locations in a random spatial sequence. The sensitivity in that region as estimated by the ZEST algorithm was subtracted from the average of the age-corrected mean normal values for those eight locations. The resulting value was the estimated GH as defined earlier and in the post-hoc calculation in the Statpac 2.^30^ The GH was subtracted from the age-corrected normative values from the SUNY-IU dataset,^25^ processed with the visualFields package^25,26^ to give the individual's reference visual field, defined as the expected thresholds for a healthy eye of the same age and GH.

#### Circle perimetry

Circle perimetry was conducted using contrasts set to a fixed offset above the expected thresholds. For example, if the expected threshold for a healthy eye of the same age and GH at a location was 30 dB and the offset was 5 dB, then the stimulus contrast would be 25 dB, corresponding to a PD value of −5 dB. Seventy-five locations were spread in three groups of 25 locations in a regular pattern along three concentric circles centred at the blind spot, with radii of 6°, 8° and 10°. At each location, the suprathreshold visual stimuli were presented at a pre-determined offset *∆ dB* from the reference visual field. At least one visual stimulus was presented at each location. If the observer responded to the stimulus presentation, then this location was marked as *seen*. When the first presentation was *not seen* at a location, then a second presentation with the same luminance was presented at that location later in the test (not immediately after the first presentation). Each location would then be categorised as seen on the first presentation, missed on the first presentation but seen on the second or missed twice. A nearest-neighbour analysis was used to identify clusters of damaged locations. A scotoma was defined as a cluster of locations for which there were non-seen trials, where at least two neighbouring locations were marked as missed twice or at least three neighbouring locations were marked as missed on the first presentation.

#### High-density perimetry

High-density perimetry was conducted only when circle perimetry identified a potential scotoma, using the same values for GH and offset. The initial test locations were determined after conducting a spatial analysis to identify clusters of damaged locations from the results of circle perimetry. High-density perimetry tested visual field locations with the goal of delineating edges of scotomata. Therefore, it iteratively selected test locations to identify the edges of each scotoma. In Stage 1, high-density perimetry started by retesting only locations in the identified clusters of damage and their immediate neighbourhood in the same region tested by circle perimetry, from 6° to 10° from the centre of the blind spot. From there, the algorithm proceeded iteratively in stages. At each stage, the radius from the centre of the blind spot was increased by 4°, so Stage 2 tested up to 14° from the centre of the blind spot, Stage 3 tested up to 18° and so on. At each stage, the locations not responded to at the edge of each cluster were identified and a curve was fitted to each edge. The fitted curves are the confines of the estimated scotoma. An additional curve was fitted to cut through the middle of the scotoma. The curves were not direct fits but were obtained after transforming the data from the Cartesian coordinates into the coordinate system used to define the Jansonius map.^33,34^ The coordinates in the Jansonius space are given by the distance from the centre of the blind spot and the angle of incidence on the optic nerve head. Linear regression of the angle of incidence as a function of distance from the blind spot yielded the curves that described the edges and centre of the scotoma, transformed back to Cartesian coordinates. At the beginning of each stage and for each scotoma, the estimates were used to:

1. project the scotoma 4° farther away from the centre of the blind spot,
2. expand both edges of the scotoma 4° outwards orthogonally with respect to its middle path,
3. select new test locations within the expanded edges that have not yet been tested,
4. present visual stimuli on the new test locations and record the participant's response and
5. update the estimated edges and middle paths of the scotoma.

The outward expansion at each scotoma in Step 2 was designed to find seen locations that surrounded the estimated scotomata. The algorithm stopped when no new test locations were selected, the scotoma reached the boundaries of the test region, typically set at 30° from the fovea (the limit for the normative data) or it was stopped by the operator.

### Protocols

Four experimental protocols assessed circle perimetry with the four groups of participants. Two protocols were conducted with the two groups of older controls to assess specificity and test–retest variability. One protocol was conducted with the group of younger controls to assess the specificity of circle perimetry followed by high-density perimetry. A further protocol was conducted with a group of patients with glaucoma to assess the performance of circle perimetry followed by high-density perimetry.

The first protocol estimated the centre of the blind spot, measured GH and conducted circle perimetry with offsets of 5, 4, 3 and 2 dB for one eye for the first group of older controls. This was part of a larger study involving imaging and perimetry, and when time allowed, the measurements were repeated on the fellow eye.

The second protocol assessed test–retest variability for GH measurements and circle perimetry with an offset of 4 dB, using one eye each for the second group of older controls. After estimating the centre of the blind spot, GH was measured twice in a row, then circle perimetry was conducted twice, after which GH was measured again. The retest variability of GH was assessed using the repeatability index, defined as 1.96 times the standard deviation of the differences between the two sets of measurements, which is one of the key elements of the mean-difference plots, also known as Bland–Altman plots.^35,36^

The third protocol assessed high-density perimetry in healthy eyes using an offset of 1 dB, with one eye each for the group of younger controls; when time allowed, the measurements were repeated on the fellow eye. After estimating the centre of the blind spot and measuring GH, circle perimetry was conducted and then high-density perimetry if it was triggered (there was a cluster of locations for which there were non-seen trials), where at least two neighbouring locations were marked as missed twice or at least three neighbouring locations were marked as missed on the first presentation.

The fourth protocol assessed circle perimetry with an offset of 5 dB, for one eye each with the group of patients with glaucoma; when time allowed, the measurements were repeated on the fellow eye. After estimating the centre of the blind spot and measuring GH, circle perimetry was conducted, and then high-density perimetry was carried out if it was triggered and there was sufficient time available.

## RESULTS

**FIGURE 1:**
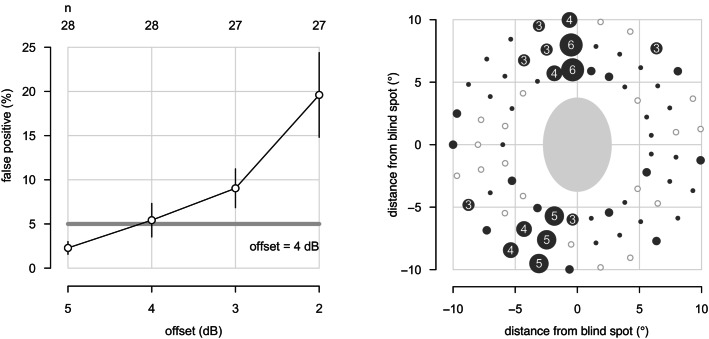
Results from the first protocol, which assessed the specificity of circle perimetry with 28 healthy older controls. Left panel: False positive rates for the four offset ∆ dB values. Open circles show the percentage of the 75 locations for which the stimulus was not seen at least once. The length of each vertical line is ± twice the standard error of the mean. The horizontal grey line represents a false positive rate of 5%. The number of eyes tested at each offset level is shown on top of the graph. Right panel: Number of eyes at each location for which the stimulus with a suprathreshold offset of 4 dB was not seen. Results are plotted in right-eye format, where a negative-x value is temporal to disc and a positive x-value is nasal to disc. Light-grey open circles indicate locations where all 28 eyes saw the initial stimulus presentation, whereas dark-grey solid circles indicate locations where at least one stimulus presentation was not seen. Circle size increases with the number of eyes for which the stimulus was not seen, from one to six (for 3–6 presentations the number is also given inside the circle).

Results for the first protocol, which assessed specificity for different offsets, are summarised in Figure 1. The left panel shows the percentage of locations for which a stimulus presentation did not elicit a response, as a function of offset; this reached ~5% with an offset of 4 dB and ~20% with an offset of 2 dB. The right panel shows the spatial distribution of false positives for an offset of 4 dB, most of which were at meridians that were consistent with angioscotomata.

**FIGURE 2:**
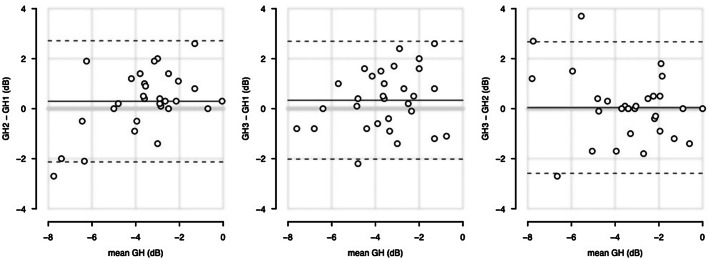
Mean-difference plots for general height (GH) estimates from the second protocol which assessed test–retest variability in 30 healthy older controls. The left panel compares the first versus the second set of GH estimates, the middle panel compares the first versus the third set of estimates and the right panel compares the second versus the third set of estimates. The *x*-axis shows the average over the two estimates, while the *y*-axis shows the difference between the two values. The solid horizontal line shows the mean difference, and the dashed lines show the mean plus and minus the repeatability index. The horizontal grey line indicates zero difference.

**FIGURE 3:**
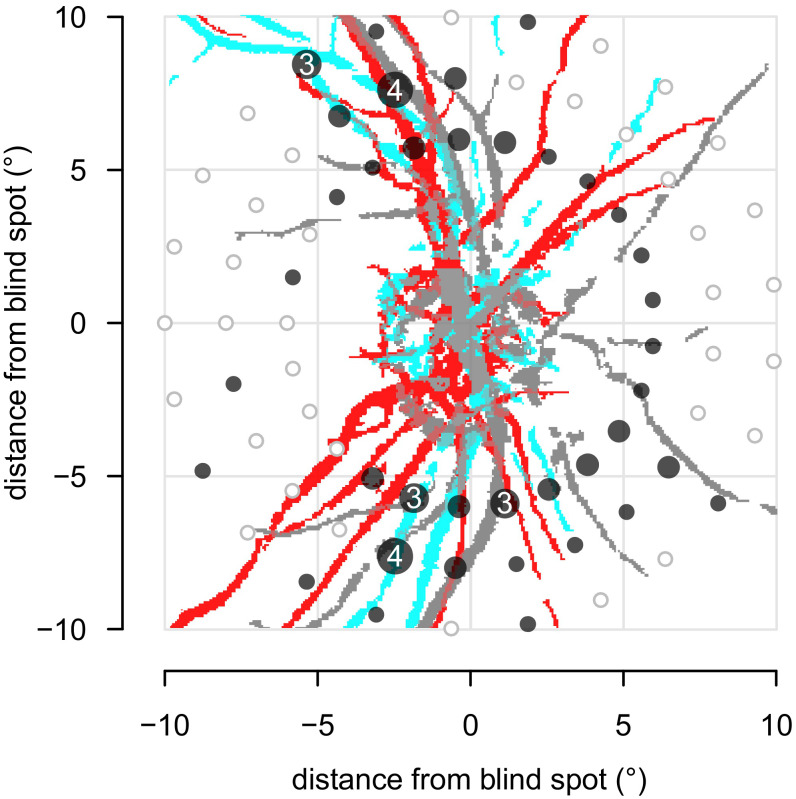
Reproducible defects after retest as a function of retinal location superimposed on an image of vessel traces from three of the subjects in the study (in different colours to indicate variability in vessel location), from the second protocol with 30 healthy older controls using circle perimetry with an offset of 4 dB. Light-grey open circles are locations with no retest defects. Dark-grey solid circles are locations for which both test and retest found a defect in at least one participant. Circle size increases with the number of retest defects found, from one to four.

Results from the second protocol, which assessed test–retest variability, are summarised in Figures 2 and 3. As shown in Figure 2, for GH the repeatability index (distance from the mean difference to the dashed lines) ranged from 2.4 to 2.6 dB over all three pairwise comparisons. For circle perimetry, an offset of 4 dB was used because that had specificity near 95% in protocol 1. Figure 3 shows locations where for both test and retest the stimulus was either not seen on both presentations or else seen on the first presentation but not seen on the second presentation; most of these were either at meridians consistent with the expected locations of angioscotomata in healthy eyes, or were on the circle closest to the blind spot where they may have fallen on the optic disc.

**FIGURE 4:**
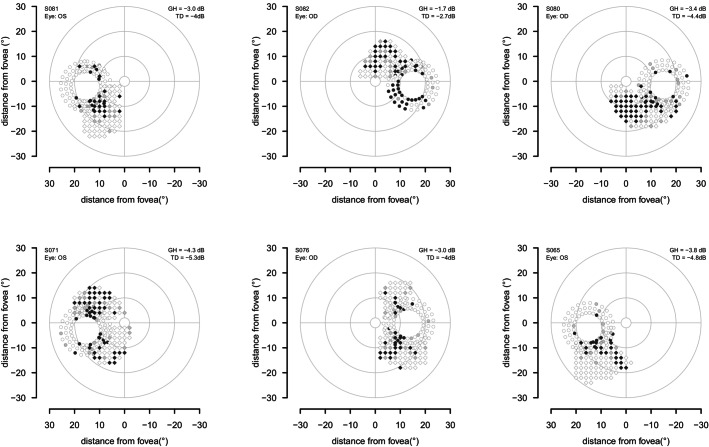
Examples of results for the third protocol, using an offset of 1 dB in healthy younger controls. Diamonds show results for high-density perimetry and circles show results for circle perimetry; black for two presentations not seen, grey for first presentation not seen and second presentation seen, white for first (and only) presentation seen. The upper left shows an example where the ‘defect’ on high-density perimetry became less clear away from the optic disc, the remainder show examples where defects were also found away from the optic disc. The value for the total deviation (TD) is the sum of the general height (GH) and the offset of −1 dB.

For the third protocol, which assessed high-density perimetry in healthy eyes, for the first stage circle perimetry was performed with an offset of 1 dB on 40 eyes of 29 healthy young controls. High-density perimetry was triggered for 26 eyes, failed to confirm a scotoma in six eyes and found an apparent defect for 20 eyes. Examples are shown in Figure 4. GH ranged from −6.8 dB to −1.7 dB, median −3.5 dB and interquartile interval −4.9 dB to −3.0 dB. Of the 11 people in whom both eyes were tested, the difference in GH for the left and right eyes ranged from −2.0 dB to +1.9 dB, median −0.6 dB, interquartile interval −0.9 dB to +0.4 dB.

**FIGURE 5:**
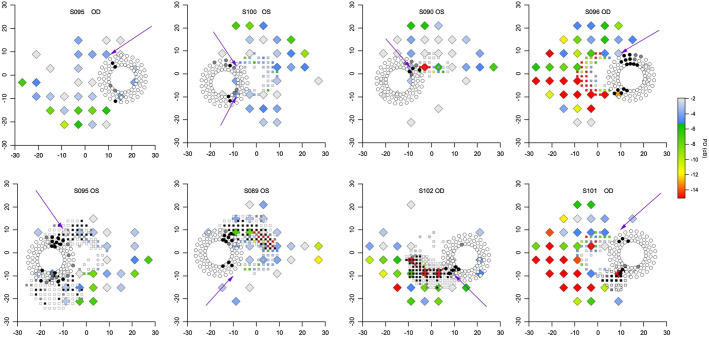
Data from eight patients with glaucoma where circle perimetry found a scotoma for a sector and 24-2 did not have pattern deviation (PD) worse than −4 dB near the optic disc, as indicated by arrows. Diamonds show 24-2 locations and coloured squares show 10-2 locations, with PD in colour as in the colour scale on the far right; for clarity, when PD was not deeper than −2 dB, no symbol is shown. Small circles show results for circle perimetry, with black for two presentations not seen, grey for first presentation not seen and second presentation seen, white for first (and only) presentation seen. The bottom row shows results for high-density perimetry (smaller squares), with white-grey-black the same as for circle perimetry.

**FIGURE 6:**
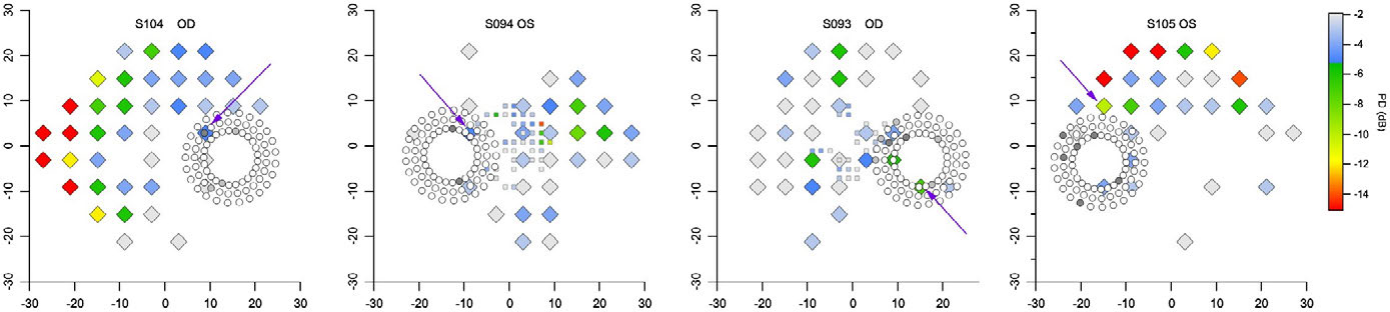
Eyes where 24-2 testing showed a pattern deviation (PD) worse than −4 dB near the optic disc and circle perimetry did not find a scotoma, as indicated by the arrows. Retinal nerve fibre layer (RNFL) thickness for the sector was outside normal limits for eyes in the two panels on the left. Symbols as in Figure 5.

For the fourth protocol, circle perimetry was performed on 26 eyes of 18 patients with glaucoma and identified scotomata in 22 sectors in 16 eyes of 13 patients; it did not identify defects in the two eyes that did not meet the criteria for visual field defect on the 24-2. Figures 5 and 6 give examples of results and Table 1 summarises findings for all patients with glaucoma. High-density perimetry was performed with a 5 dB offset for 14 sectors in 11 of these 16 eyes and found arcuate defects in all 14 sectors tested. The 24-2 grid identified a perimetric location near the optic disc with PD of −5 dB or worse for 13 of the 22 sectors where circle perimetry found a scotoma. Figure 5 shows data from the eight patients with a sector where PD was not worse than −4 dB but circle perimetry found a scotoma for the sector (indicated with an arrow); for seven of these eyes, high-density perimetry and/or 10-2 data were available which confirmed the presence of a scotoma. Of the 10 eyes for which circle perimetry did not find a scotoma, the 24−2 identified 4 cases with a perimetric location near the disc with PD of −5 dB or worse; results for these eyes are shown in Figure 6. Three of these sectors had perimetric defects in the hemifield (two or more adjoining locations with PD values at *p* < 0.01). Of the 22 sectors for which circle perimetry identified scotomata, circumpapillary RNFL thickness flagged 20 as below the 1st percentile (‘Outside Normal Limits’, ONL) and two below the 5th percentile (‘Borderline’, BL). Circumpapillary RNFL thickness scored an additional 12 sectors as below the 1st percentile and another eight as below the 5th percentile. Individual results for all protocols can be found in Appendix S1.

**TABLE 1:** Results for protocol 4, circle perimetry for 26 eyes of 18 patients with glaucoma. ST and IT represent results for the superior-temporal and inferior-temporal sectors, respectively.

ID	Eye	ST scotoma	IT scotoma	ST dB	IT dB	GH dB	ST score	IT score	ST μm	IT μm
S091	OS	Yes	Yes	−31	−7	−4.5	ONL	ONL	27	41
S101	OD	Yes	Yes	−20	−1	−5.3	ONL	ONL	61	68
S096	OD	Yes	Yes	−13	−3	−5.3	ONL	ONL	51	51
S095	OS	Yes	Yes	−5	−2	−0.3	ONL	ONL	65	91
S089	OS	Yes	Yes	−1	−7	−8.8	BL	ONL	93	28
S100	OS	Yes	Yes	−4	−2	−4.2	ONL	ONL	82	58
S102	OS	Yes	No	−33	−3	−1.7	ONL	ONL	28	95
S093	OS	Yes	No	−10	−4	−3.1	ONL	WNL	46	129
S102	OD	Yes	No	−3	−1	−1.7	ONL	WNL	55	125
S103	OD	No	Yes	−2	−33	−3.5	WNL	ONL	125	64
S092	OS	No	Yes	−4	−29	−5.3	BL	ONL	92	47
S097	OS	No	Yes	−1	−14	−5.2	WNL	ONL	120	75
S100	OD	No	Yes	−5	−6	−4.3	ONL	ONL	76	42
S088	OS	No	Yes	−6	−5	−7	BL	ONL	92	79
S090	OS	No	Yes	−3	−4	−4.8	BL	ONL	94	51
S095	OD	No	Yes	−4	−4	−1.8	ONL	BL	83	94
S105	OS	No	No	−4	−10	−5.7	BL	BL	83	91
S093	OD	No	No	−7	−4	−0.7	WNL	WNL	107	150
S094	OS	No	No	−3	−5	−2.4	WNL	ONL	116	52
S104	OD	No	No	−2	−5	−3.5	BL	ONL	99	75
S097	OD	No	No	−2	−4	−2.4	WNL	WNL	104	133
S099	OS	No	No	−2	−3	−5.2	WNL	BL	108	97
S099	OD	No	No	−2	−3	−6.2	WNL	WNL	109	117
S098	OS	No	No	−3	−2	−3.3	BL	WNL	76	96
S101	OS	No	No	−2	−2	−5.8	WNL	WNL	106	122
S091	OD	No	No	−1	0	−1	ONL	ONL	65	69

*Note*: The columns labelled ‘Scotoma’ have ‘Yes’ when circle perimetry found a scotoma for the sector, and ‘No’ when it did not. The values in dB (decibel) are from the 24-2 and give the deepest pattern deviation (PD) near the optic disc for the sector. GH gives the General Height for the eye. ‘Score’ is how machine norms labelled the sector: Outside normal limits (ONL) indicates below the 1st percentile, borderline (BL) indicates below the 5th percentile, within normal limits (WNL) indicates above the 5th percentile. The values in μm give the retinal nerve fibre layer (RNFL) thickness for the sector. 24-2 visual fields met the criteria for perimetric defect for all eyes except S097 OD and S101 OS. OD, right eye; OS, left eye.

## DISCUSSION

Targeted perimetry with dense spacing of perimetric locations can improve the ability to detect and delineate glaucomatous scotomata,^2,4,14,15^ and has the potential to improve structure–function relations and assessment of progression. Before targeted perimetry can be used clinically, studies are needed to understand its potential and challenges better. The current investigation sought methods that are relatively unaffected by normal inhomogeneities in perimetric sensitivity, by testing near the optic disc where shadows of blood vessels cause angioscotomata in healthy eyes. These need to be distinguished from glaucomatous scotomata that are connected to the blind spot. The success of this approach supports the further development of suprathreshold algorithms that choose locations adaptively, depending on the eye's overall visual field sensitivity and the shape and size of its scotomata. Understanding the challenges encountered can provide guidance for future development of algorithms with dense spacing.

Results from control subjects showed that contrasts of more than 4 dB above expected thresholds may be required to keep high specificity, which may limit the ability to detect mild glaucomatous defects. Of the four patients with glaucoma from Figure 6 who did not have scotomata on circle perimetry with an offset of 5 dB, despite a PD worse than –4 dB on the 24-2, three were also tested with circle perimetry using an offset of 3 dB. Scotomata were found in the hemifields with 24-2 defects (superior and inferior for S104, superior for S094 and inferior for S093). Results from controls tested with an offset of 1 dB found that circle perimetry often identified defects that high-density perimetry confirmed with that offset, indicating that using lower offsets risks having a high false alarm rate. It may be better to search for scotomata without assuming an arcuate path, unless large offsets are used.

Results from patients with glaucoma showed that when circle perimetry with an offset of 5 dB found a scotoma near the optic disc, then a scotoma connected to the optic disc was confirmed with the 10-2 or high-density perimetry, if those were available. This is further evidence that an offset of 5 dB has good specificity, but due to the small number of subjects, a precise estimate of specificity was not possible. More work is needed to determine how to use contrasts that can yield both high specificity and good ability to detect defects.

Perimetry uses relatively small visual stimuli, for which contrast thresholds in healthy eyes increase monotonically with the distance from the fovea (i.e., eccentricity). The three-dimensional profile of sensitivity with retinal location gives the impression of a hill, which is why the geographical map of sensitivities spanning the visual field is called the ‘hill of vision’.^37^ Operationally, inter-individual differences in the hill of vision among healthy eyes can be separated into differences in overall sensitivity—also known as GH,^31,38^ and as differences in shape—or how rapidly sensitivity decreases with eccentricity.^39^ There are systematic decreases in sensitivity with age that can be partially corrected using pointwise linear models,^40^ but large inter-individual differences still remain.^41^ Current diagnostic methods for static perimetry reduce these differences by assessing deviation from the ‘pattern’ of the hill of vision^31,38^ after removing the estimated effect of the GH. These estimates of PD are computed after testing is completed and are subject to statistical artefacts in eyes with scotomata.^22,42^ In this study, GH was estimated at the start of testing and this was used to adjust normative data in order to select stimulus contrasts equivalent to a PD value of −5 dB.

The importance of the use of GH is illustrated in Figure 4, where incorporating a 1 dB offset often yielded results in healthy eyes that resembled glaucomatous scotomata. The GH values used in these examples ranged from −1.7 dB to −4.3 dB, which means that contrasts ranged from 2.7 dB to 5.3 dB above mean normal contrast threshold for a location. A contrast threshold 5 dB above mean normal is referred to as a total deviation (TD) of −5 dB, which is at about the 2nd percentile for these locations. On a TD map, the pattern in the lower left of Figure 4 would be a set of adjoining locations worse than −5 dB, that would look like a glaucomatous scotoma. However, when GH is accounted for, these contrasts correspond to a PD of −1 dB, which is well within the normal range and would not be mistaken for a glaucomatous scotoma. Given the importance of GH, a challenge is that test–retest variability of GH was sufficiently high that a difference of 2 dB on retest was not unusual. For future use of targeted perimetry, it may be helpful to develop methods for assessing GH having better repeatability.

The average value for GH over repeated estimates and all participants was −3.5 dB, but a mean near zero would be expected based on the normative data. The normative data were gathered with the Humphrey Field Analyzer (HFA), whereas this study used the Compass perimeter.^43^ The contrast sensitivities measured with the Compass instrument were found to be 1–2 dB lower, on average, than for the HFA,^43^ so it is not surprising that the average value for GH determined here was negative. Further work is justified to assess methods for measuring GH.

The first stage presented the stimulus at 75 locations around the optic disc, but the locations where it was not seen usually corresponded with the superior temporal and inferior temporal sectors of the optic disc. This study assessed the locations of false positives, for which purpose it was useful to test locations all around the disc. Future studies could use a smaller number of initial positions as the basis for selecting customised locations. The first stage required estimation of the location of the optic disc because the goal was to find defects that reached the disc, but future algorithms could use locations based on the type of defect being sought.

This study focused on defects that extended to the optic disc because this is a region in which healthy eyes have relatively large changes in sensitivity with location due to shadows of the blood vessels.^21^ Therefore, the low false positive rate for the 5 dB offset is likely to apply elsewhere in the normal visual field, and smaller offsets may also have low false positive rates when used in regions farther from the optic disc.

In summary, this study used suprathreshold testing of targeted densely spaced perimetric locations near the optic disc, where inhomogeneities in sensitivity are caused by shadows of blood vessels. It was determined that contrasts 5 dB greater than expected thresholds were needed to avoid false positives in control eyes. Use of these contrasts limited the ability to detect mild glaucomatous damage, but when these contrasts did identify damage then an arcuate defect could be delineated which connected to the blind spot. In order for targeted perimetry to be suitable for clinical use, more research is needed to optimise the contrast levels used and to find more appropriate locations other than near the optic disc. This study selected locations based on the expected paths of nerve fibre bundles when delineating scotomata, but that method seems unsuitable because it led to arcuate defects in healthy eyes when contrasts were low. Since targeted perimetry can delineate the shapes of scotomata, future research could assess structure–function concordance in terms of spatial maps.

## Supplementary Information

**Appendix S1.** (ZIP 3.30 MB)

## References

[1] Maddess T. The influence of sampling errors on test-retest variability in perimetry. *Invest Ophthalmol Vis Sci.* 2011;52:1014–1022. 21051713 10.1167/iovs.10-6014

[2] Schiefer U, Malsam A, Flad M, Stumpp F, Dietrich TJ, Paetzold J, et al. Evaluation of glaucomatous visual field loss with locally condensed grids using fundus-oriented perimetry (FOP). *Eur J Ophthalmol.* 2001;11:S57–S62. 11592532 10.1177/112067210101102s07

[3] Numata T, Matsumoto C, Okuyama S, Tanabe F, Hashimoto S, Nomoto H, et al. Detectability of visual field defects in glaucoma with high-resolution perimetry. *J Glaucoma.* 2016;25:847–853. 27367134 10.1097/IJG.0000000000000460

[4] Westcott MC, Garway-Heath DF, Fitzke FW, Kamal D, Hitchings RA. Use of high spatial resolution perimetry to identify scotomata not apparent with conventional perimetry in the nasal field of glaucomatous subjects. *Br J Ophthalmol.* 2002;86:761–766. 12084745 10.1136/bjo.86.7.761 PMC1771187

[5] Hood DC, Raza AS, de Moraes CG, Liebmann JM, Ritch R. Glaucomatous damage of the macula. Prog Retin Eye Res. 2013;32. 10.1016/j.preteyeres.2012.08.003 10.1016/j.preteyeres.2012.08.003 PMC3529818 22995953

[6] De Moraes CG, Hood DC, Thenappan A, Girkin CA, Medeiros FA, Weinreb RN, et al. 24-2 visual fields miss central defects shown on 10-2 tests in glaucoma suspects, ocular hypertensives and early glaucoma. *Ophthalmology.* 2017;124:1449–1456. 28551166 10.1016/j.ophtha.2017.04.021 PMC5610609

[7] Sullivan-Mee M, Karin Tran MT, Pensyl D, Tsan G, Katiyar S. Prevalence, features and severity of glaucomatous visual field loss measured with the 10-2 achromatic threshold visual field test. *Am J Ophthalmol.* 2016;168:40–51. 27173372 10.1016/j.ajo.2016.05.003

[8] Hood DC, La Bruna S, Tsamis E, Leshno A, Melchior B, Grossman J, et al. The 24-2 visual field guided progression analysis can miss the progression of glaucomatous damage of the macula seen using OCT. *Ophthalmol Glaucoma.* 2022;5:614–627. 35358755 10.1016/j.ogla.2022.03.007 PMC9515237

[9] Artes PH, Henson DB, Harper R, McLeod D. Multisampling suprathreshold perimetry: a comparison with conventional suprathreshold and full-threshold strategies by computer simulation. *Invest Ophthalmol Vis Sci.* 2003;44:2582–2587. 12766060 10.1167/iovs.02-1036

[10] Henson DB. Visual field screening and the development of a new screening program. *J Am Optom Assoc.* 1989;60:893–898. 2693509

[11] Henson DB, Artes PH. New developments in supra-threshold perimetry. *Ophthalmic Physiol Opt.* 2002;22:463–468. 12358319 10.1046/j.1475-1313.2002.00055.x

[12] Johnson CA, Keltner JL, Balestrery FG. Suprathreshold static perimetry in glaucoma and other optic nerve disease. *Ophthalmology.* 1979;86:1278–1286. 233860 10.1016/s0161-6420(79)35399-4

[13] Sukumar S, Harper RA, Tsamis E, Hood D, Henson DB. Diagnostic accuracy of Smart Supra perimetry in comparison with standard automated perimetry in the detection of early glaucoma. *J Glaucoma.* 2025;34:710–718. 40366217 10.1097/IJG.0000000000002596 PMC13266405

[14] Jones PR. MEDTEG (minimum entropy dynamic test grids): a novel algorithm for adding new test locations to a perimetric test grid. *Transl Vis Sci Technol.* 2025;14:25. 10.1167/tvst.14.2.25 10.1167/tvst.14.2.25 PMC11881782 40009356

[15] Denniss J, McKendrick AM, Turpin A. Suprathreshold approaches to mapping the visual field in advanced glaucoma. *Transl Vis Sci Technol.* 2023;12:19. 10.1167/tvst.12.6.19 10.1167/tvst.12.6.19 PMC10297785 37358492

[16] Alluwimi MS, Swanson WH, Malik R. Structure-function assessment in glaucoma based on perimetric sensitivity and en face optical coherence tomography images of retinal nerve fiber bundles. *Sci Rep.* 2023;13:2497. 10.1038/s41598-023-28917-1 36781886 10.1038/s41598-023-28917-1 PMC9925735

[17] Chauhan BC, Henson DB, Hobley AJ. Cluster analysis in visual field quantification. *Doc Ophthalmol.* 1988;69:25–39. 3168709 10.1007/BF00154416

[18] Zulauf M. Quantification of angioscotomas. *Ophthalmologica.* 1990;200:203–209. 2367084 10.1159/000310108

[19] Remky A, Beausencourt E, Elsner AE. Angioscotometry with the scanning laser ophthalmoscope. Comparison of the effect of different wavelengths. *Invest Ophthalmol Vis Sci.* 1996;37:2350–2355. 8843920

[20] Schiefer U, Benda N, Dietrich TJ, Selig B, Hofmann C, Schiller J. Angioscotoma detection with fundus-oriented perimetry. A study with dark and bright stimuli of different sizes. *Vis Res.* 1999;39:1897–1909. 10343881 10.1016/s0042-6989(98)00295-8

[21] Marin-Franch I, Wyatt HJ, Swanson WH. Using high-density perimetry to explore new approaches for characterizing visual field defects. *Vis Res.* 2023;210:108259. 10.1016/j.visres.2023.108259 37285782 10.1016/j.visres.2023.108259 PMC10526895

[22] Marin-Franch I, Swanson WH, Malinovsky VE. A novel strategy for the estimation of the general height of the visual field in patients with glaucoma. *Graefes Arch Clin Exp Ophthalmol.* 2014;252:801–809. 24638255 10.1007/s00417-014-2602-x PMC4079702

[23] Swanson WH, Malinovsky VE, Dul MW, Malik R, Torbit JK, Sutton BM, et al. Contrast sensitivity perimetry and clinical measures of glaucomatous damage. *Optom Vis Sci*. 2014;91:1302–1311. 25259758 10.1097/OPX.0000000000000395 PMC4243800

[24] Turpin A, Artes PH, McKendrick AM. The Open Perimetry Interface: an enabling tool for clinical visual psychophysics. *J Vis.* 2012;12:22. 10.1167/12.11.22 10.1167/12.11.22 23104815

[25] Marin-Franch I, Swanson WH. The visualFields package: a tool for analysis and visualization of visual fields. *J Vis.* 2013;13:10. 10.1167/13.4.10 10.1167/13.4.10 PMC3600987 23492926

[26] Marin-Franch I, Turpin A, Artes PH, Chong LX, McKendrick AM, Alawa KA, et al. The Open Perimetry Initiative: a framework for cross-platform development for the new generation of portable perimeters. *J Vis.* 2022;22:1. 10.1167/jov.22.5.1 10.1167/jov.22.5.1 PMC8994165 35385053

[27] Rossetti L, Digiuni M, Rosso A, Riva R, Barbaro G, Smolek MK, et al. Compass: clinical evaluation of a new instrument for the diagnosis of glaucoma. *PLoS One.* 2015;10:e0122157. 10.1371/journal.pone.0122157 25807241 10.1371/journal.pone.0122157 PMC4373771

[28] R Core Team. R: a language and environment for statistical computing. Vienna, Austria: R Foundation for Statistical Computing; 2024. Available from: https://cran.r-project.org/package=shiny. Accessed Sept 5, 2025.

[29] Chang WCJ, Allaire J, Sievert C, Schloerke B, Xie Y, Allen J, et al. shiny: Web Application Framework for R. 2024. R package version 1.9.1.9000. Available from: https://shiny.posit.co/. Accessed Sept 5, 2025.

[30] Heijl A, Lindgren G, Lindgren A, Olsson J, Åsman P, Myer JS, et al. Extended empirical statistical package for evaluation of single and multiple fields in glaucoma: Statpac 2. In: Mills RP, Heijl A, editors. Perimetry update 1990/1991. New York: Kugler & Ghedin; 1991. p. 303–315.

[31] Heijl A, Lindgren G, Olsson J. A package for the statistical analysis of visual fields. *Doc Ophthalmol Proc Ser.* 1987;49:153–168.

[32] King-Smith PE, Grigsby SS, Vingrys AJ, Benes SC, Supowit A. Efficient and unbiased modifications of the QUEST threshold method: theory, simulations, experimental evaluation and practical implementation. *Vis Res.* 1994;34:885–912. 8160402 10.1016/0042-6989(94)90039-6

[33] Jansonius NM, Nevalainen J, Selig B, Zangwill LM, Sample PA, Budde WM, et al. A mathematical description of nerve fiber bundle trajectories and their variability in the human retina. *Vis Res.* 2009;49:2157–2163. 19539641 10.1016/j.visres.2009.04.029 PMC2848177

[34] Jansonius NM, Schiefer J, Nevalainen J, Paetzold J, Schiefer U. A mathematical model for describing the retinal nerve fiber bundle trajectories in the human eye: average course, variability and influence of refraction, optic disc size and optic disc position. *Exp Eye Res.* 2012;105:70–78. 23099334 10.1016/j.exer.2012.10.008

[35] Altman DG, Bland JM. Measurement in medicine: the analysis of method comparison studies. *The Statistician.* 1983;32:307–318.

[36] Bland JM, Altman DG. Statistical methods for assessing agreement between two methods of clinical measurement. *Lancet.* 1986;1:307–310. 2868172

[37] Traquair HM. An introduction to clinical perimetry. London: Henry Kimpton; 1938.

[38] Heijl A, Lindgren G, Olsson J, Asman P. Visual field interpretation with empiric probability maps. *Arch Ophthalmol.* 1989;107:204–208. 2916973 10.1001/archopht.1989.01070010210024

[39] Swanson WH, Dul MW, Horner DG, Malinovsky VE. Individual differences in the shape of the nasal visual field. *Vis Res.* 2017;141:23–29. 27187584 10.1016/j.visres.2016.04.001 PMC5161726

[40] Bengtsson B, Heijl A. Inter-subject variability and normal limits of the SITA standard, SITA fast and the Humphrey full threshold computerized perimetry strategies, SITA STATPAC. *Acta Ophthalmol Scand.* 1999;77:125–129. 10321523 10.1034/j.1600-0420.1999.770201.x

[41] Ashimatey BS, Swanson WH. Between-subject variability in healthy eyes as a primary source of structural-functional discordance in patients with glaucoma. *Invest Ophthalmol Vis Sci.* 2016;57:502–507. 26873511 10.1167/iovs.15-18633 PMC4758296

[42] Blumenthal EZ, Sapir-Pichhadze R. Misleading statistical calculations in far-advanced glaucomatous visual field loss. *Ophthalmology.* 2003;110:196–200. 12511366 10.1016/s0161-6420(02)01297-6

[43] Montesano G, Bryan SR, Crabb DP, Fogagnolo P, Oddone F, McKendrick AM, et al. A comparison between the Compass fundus perimeter and the Humphrey field analyzer. *Ophthalmology.* 2019;126:242–251. 30114416 10.1016/j.ophtha.2018.08.010

