# Supplementary figures and images for: Targeted perimetry for mapping the shape of glaucomatous arcuate scotomata
Source: Ophthalmic Physiol Opt. 2025 Sep 8;45(7):2025–35. doi: 10.1111/opo.70014 (PMC12682095; doi:10.1111/opo.70014)

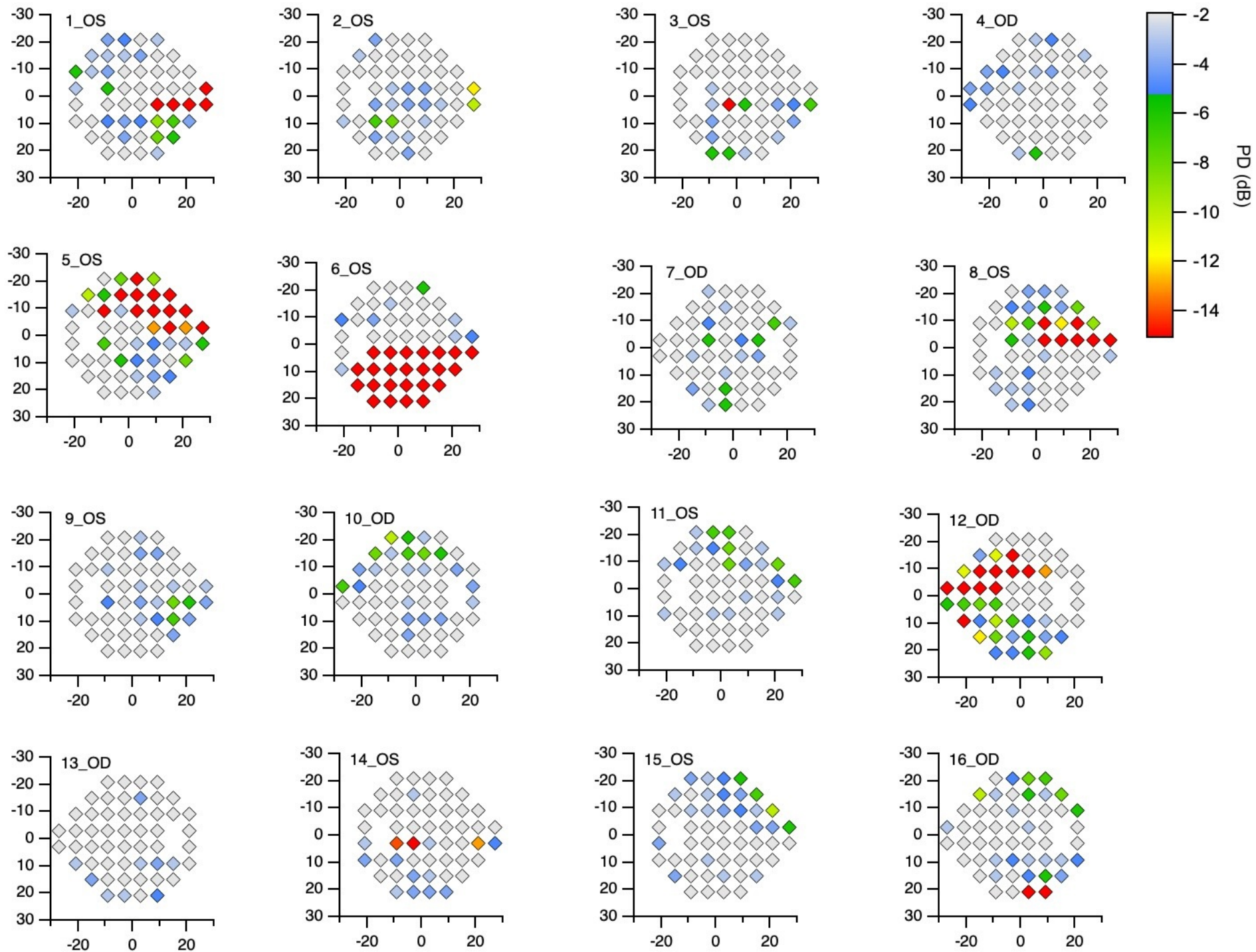

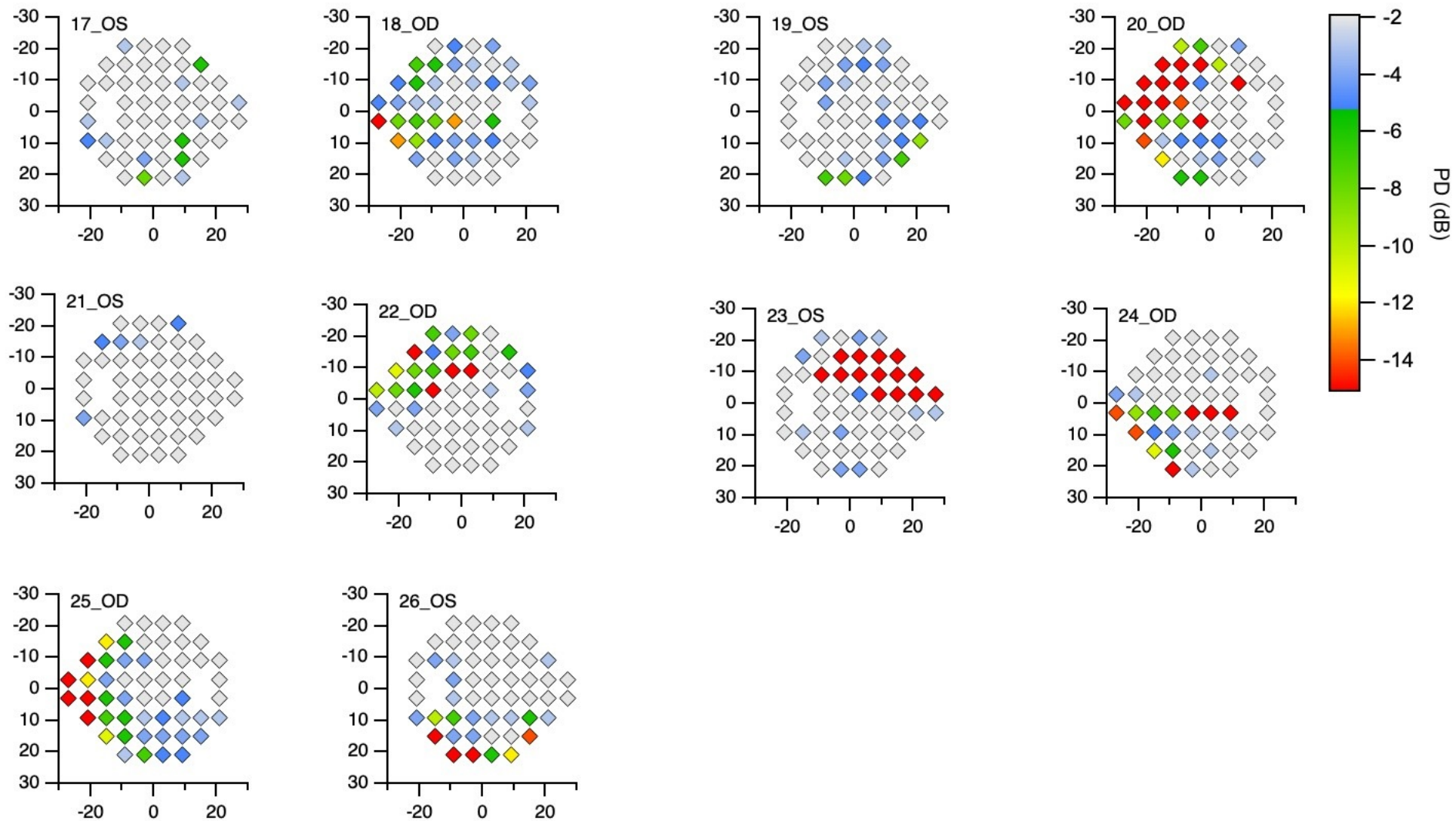

Supplement: Supplementary file 1 — Appendix S1. (ZIP 3.30 MB) [file 44402_2025_4507042_MOESM1_ESM.zip › opo70014-sup-0001-Supinfo1.pdf]

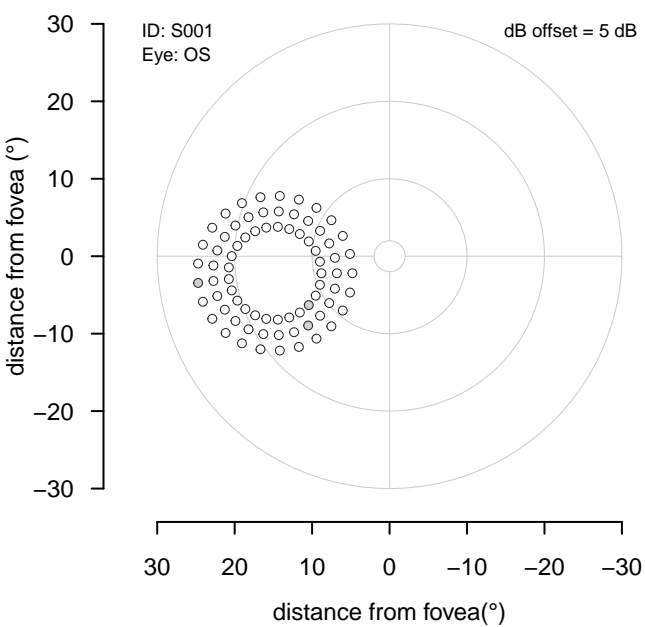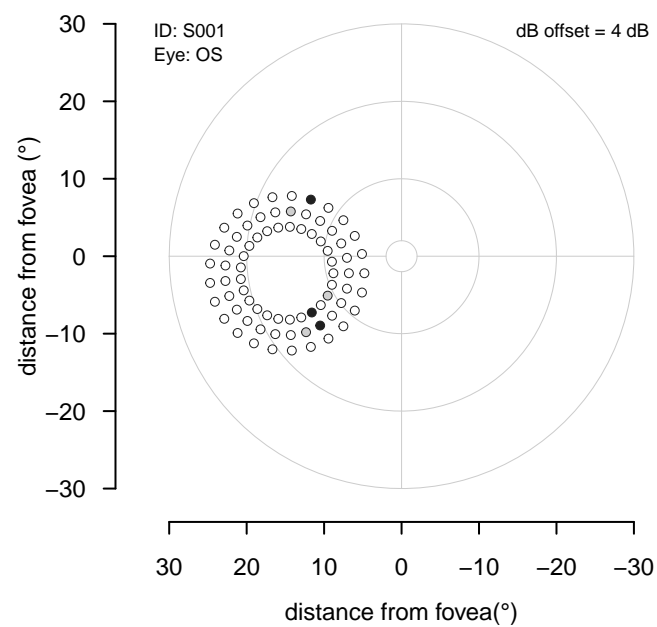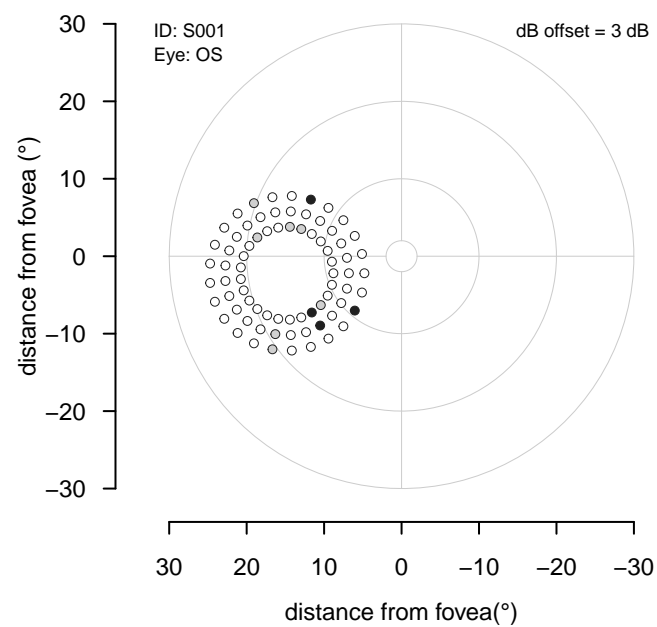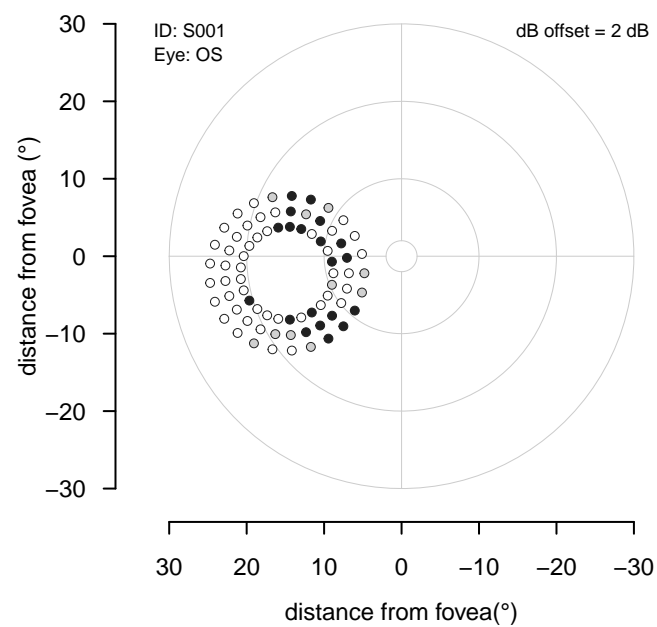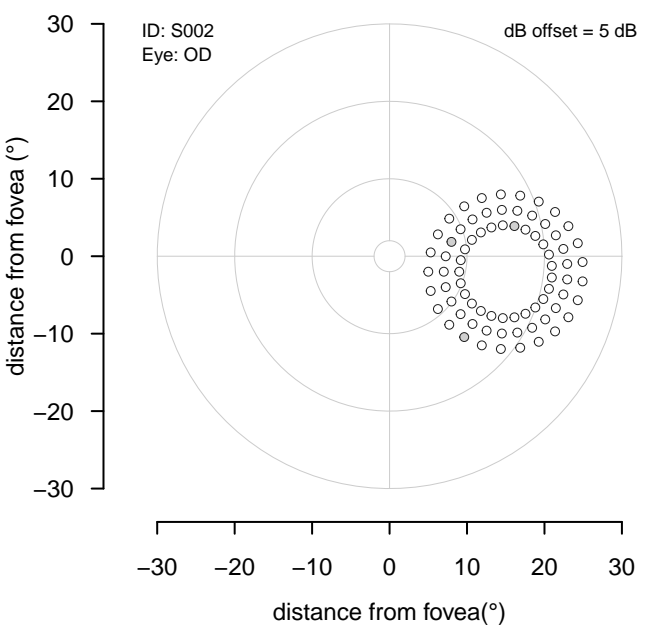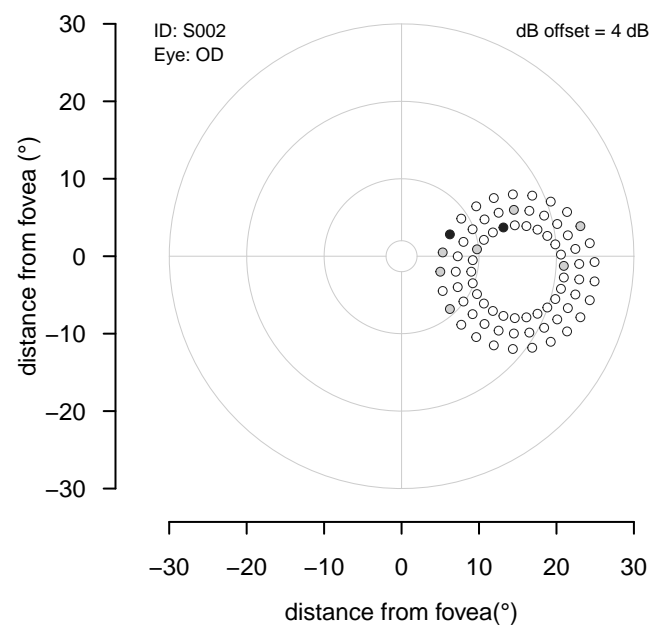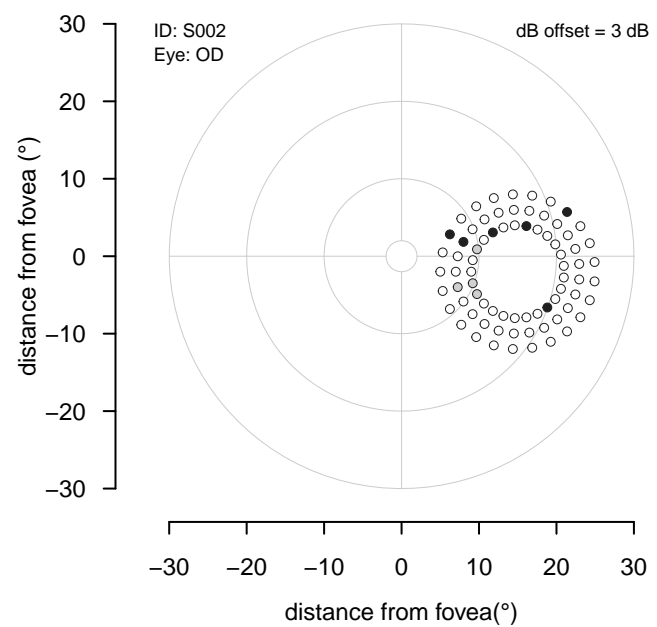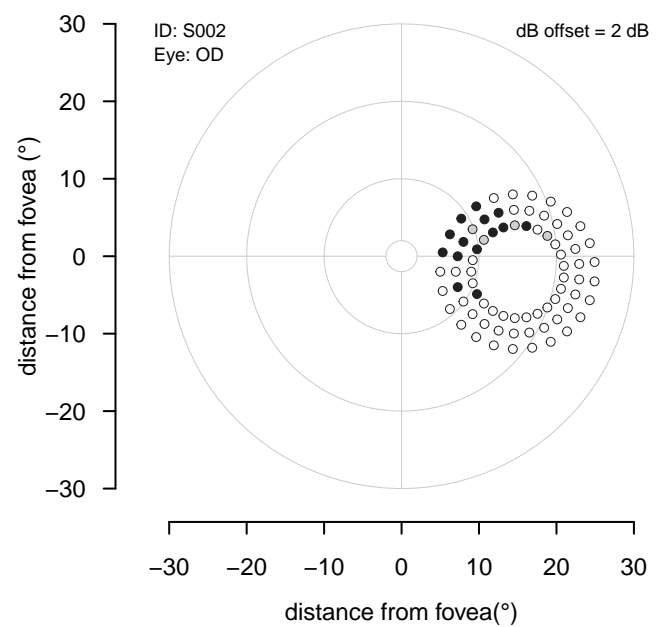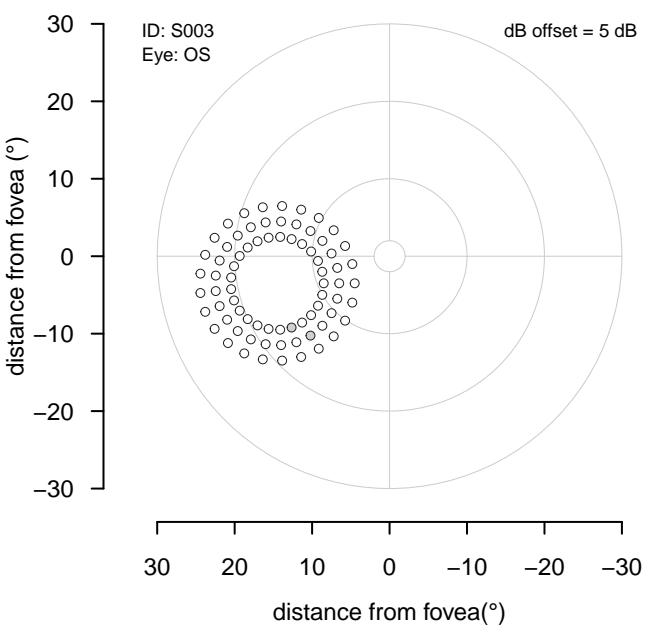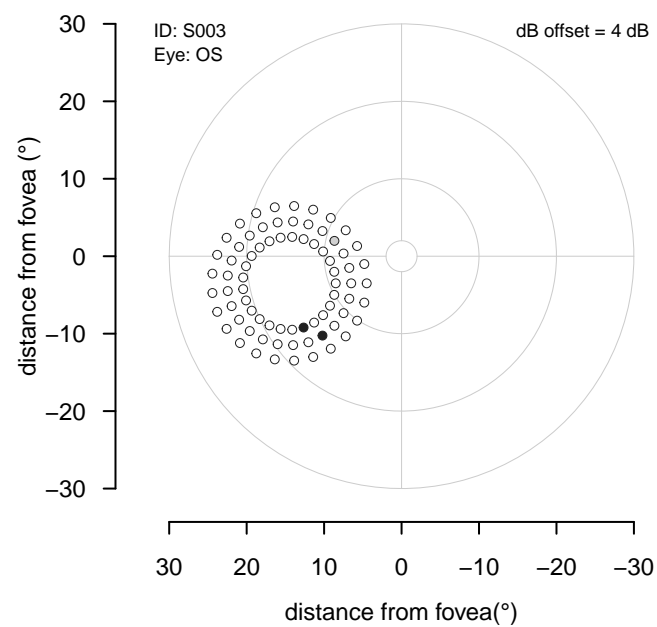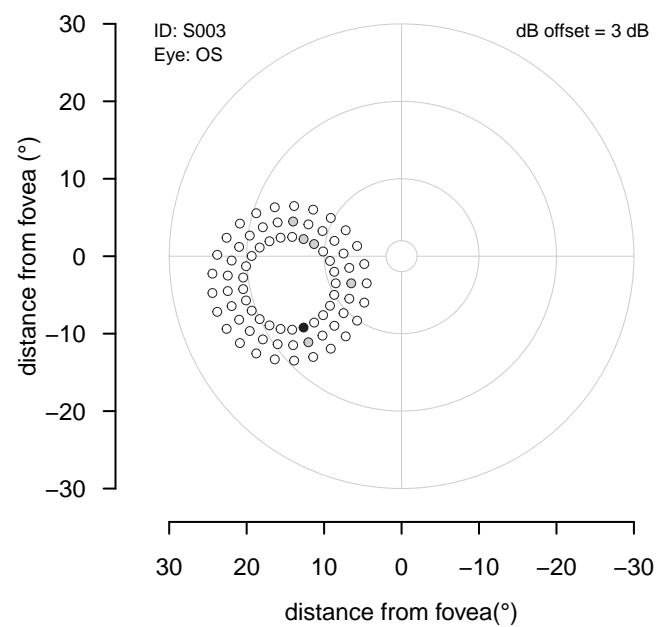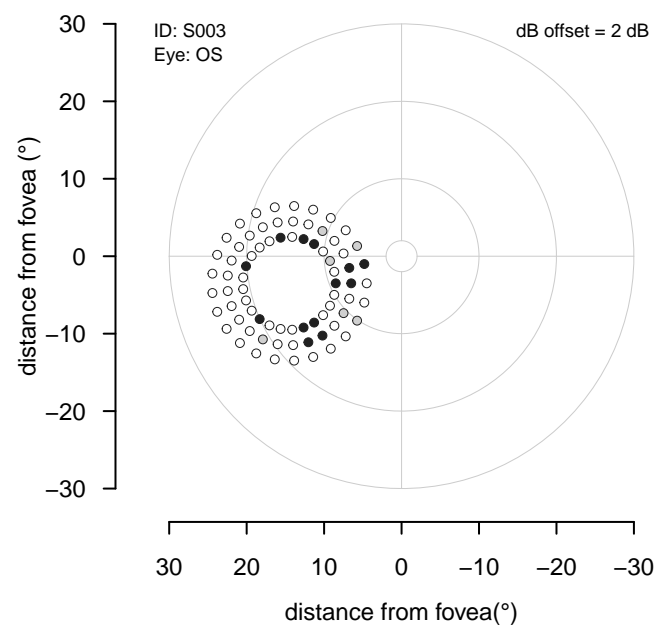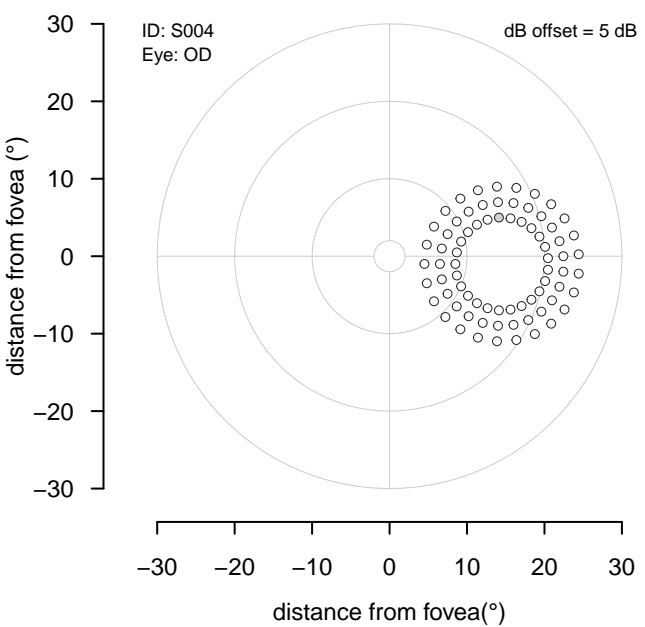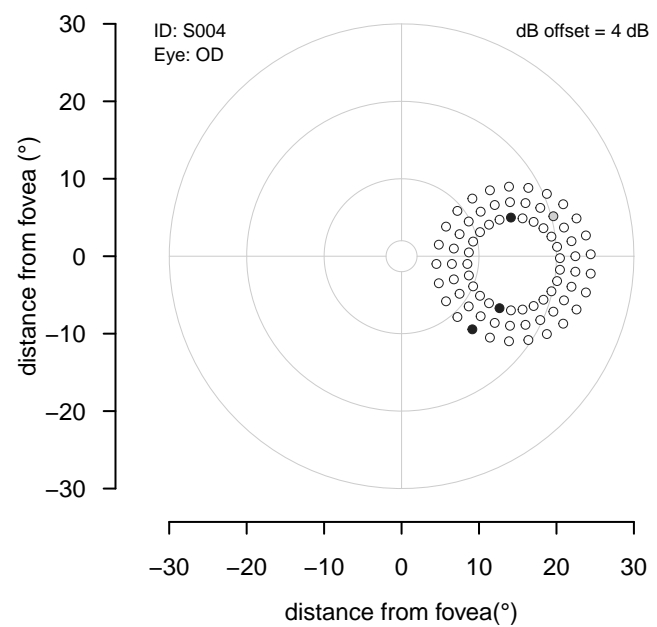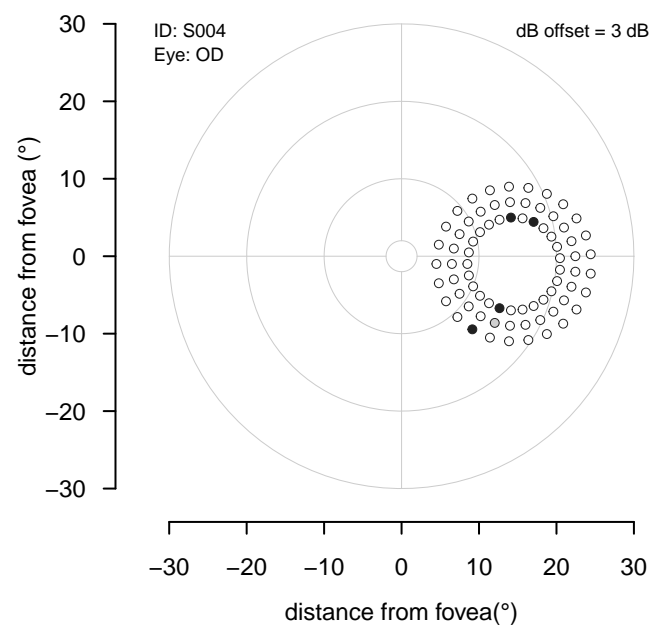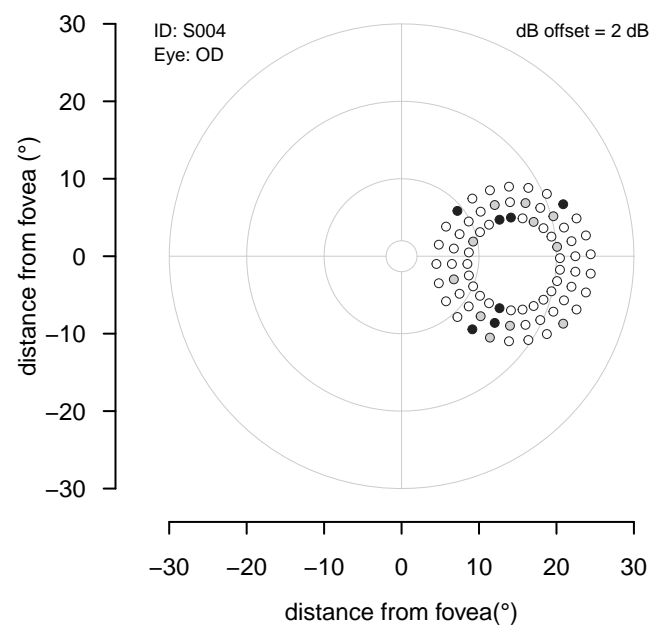

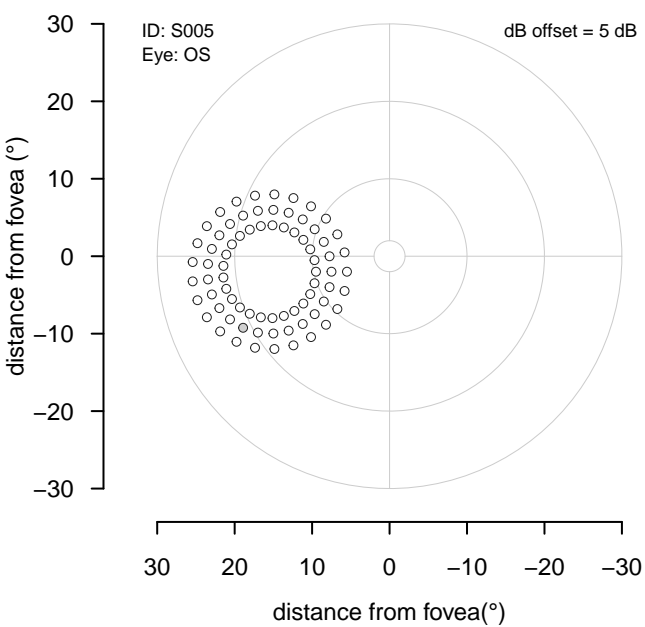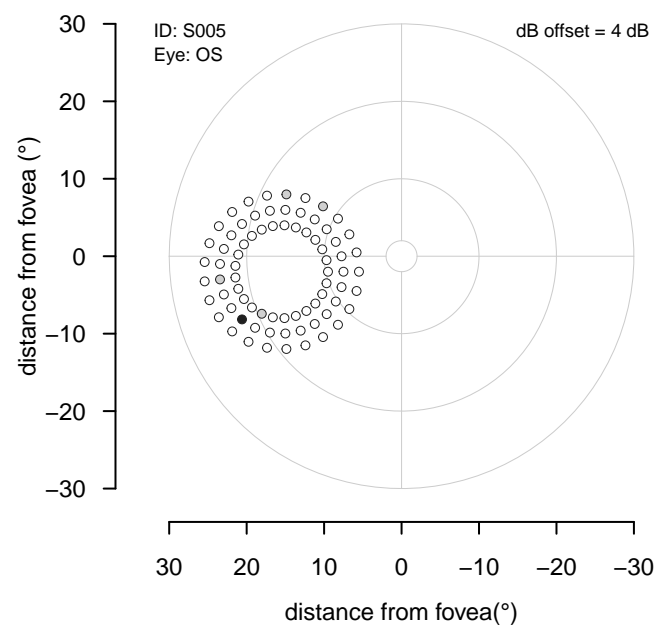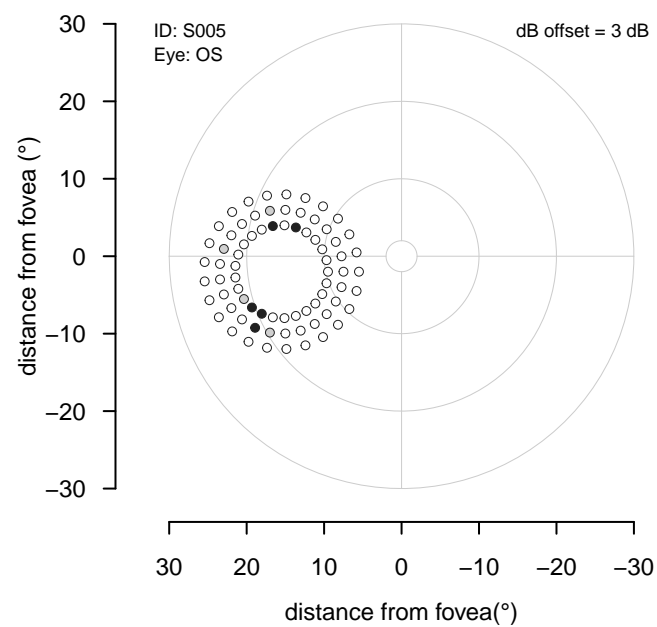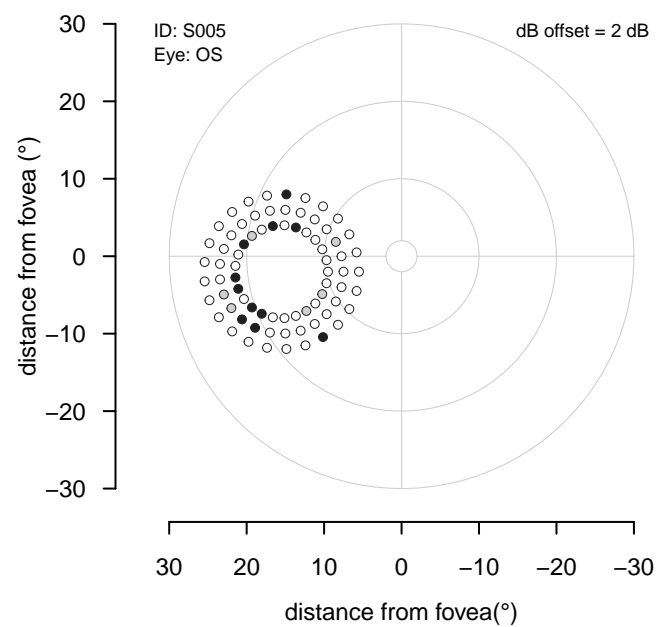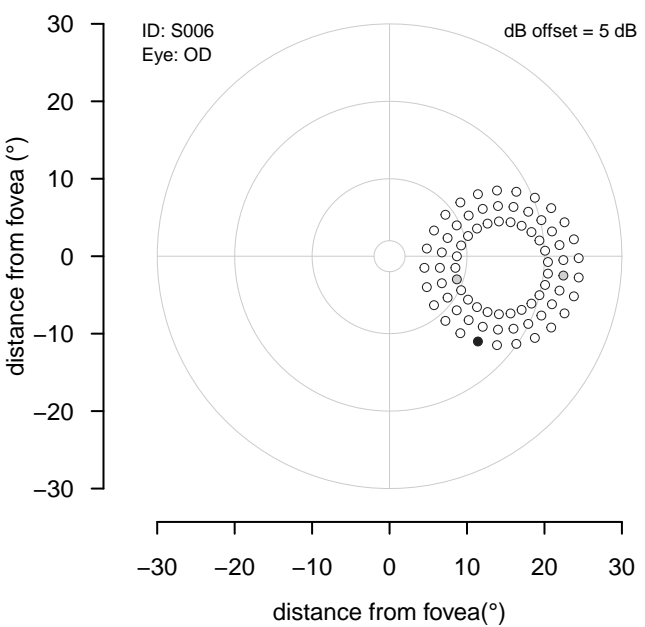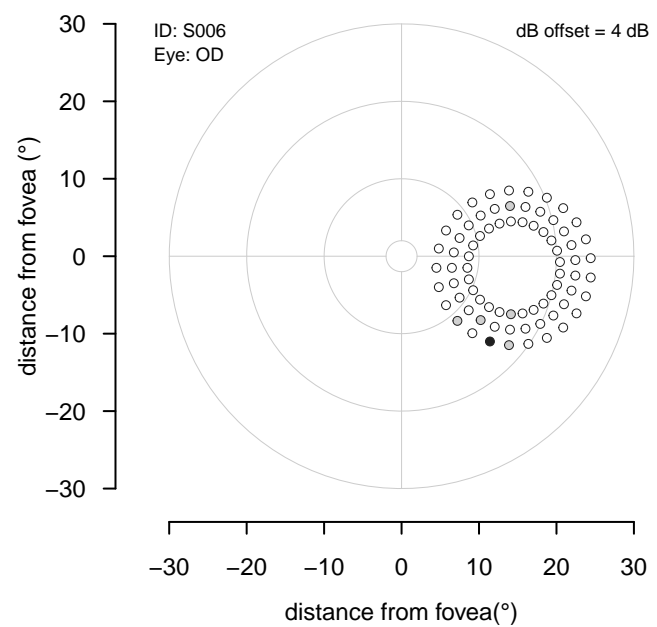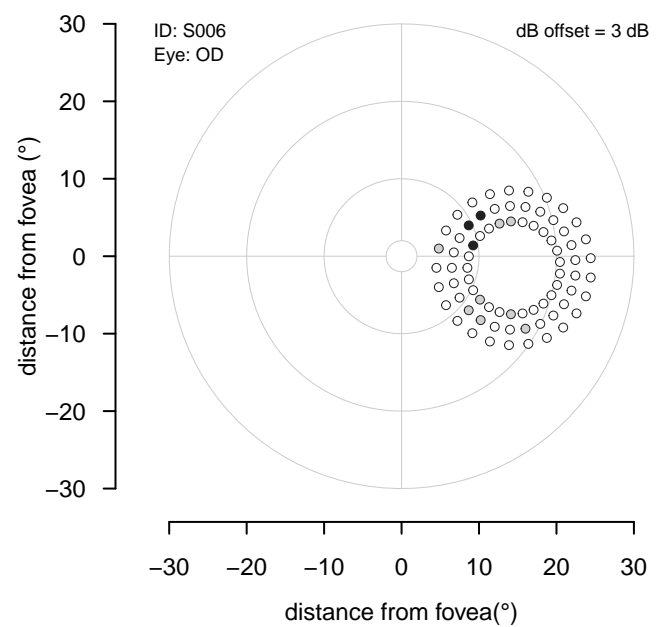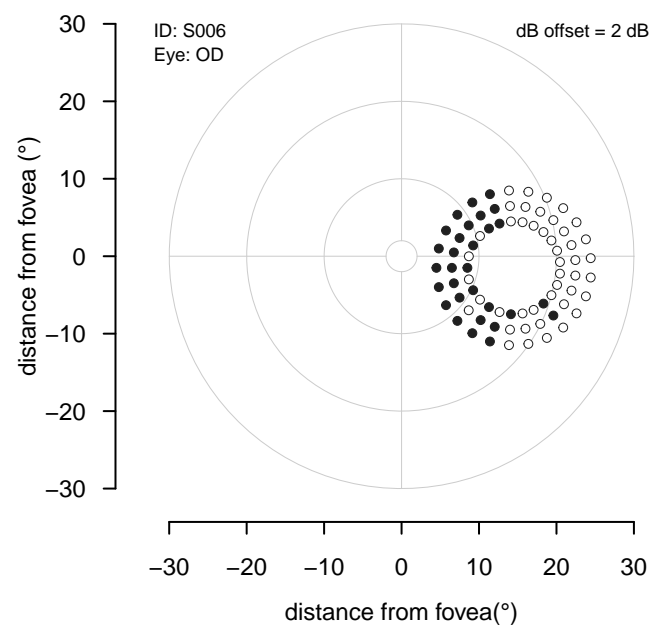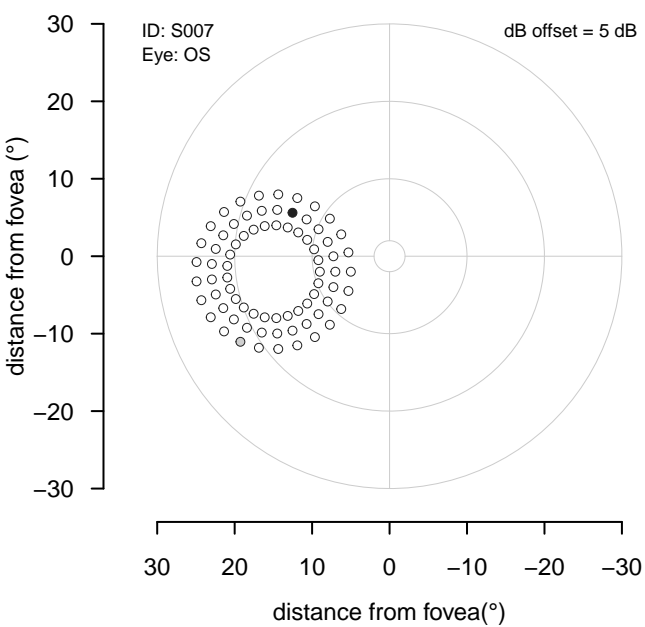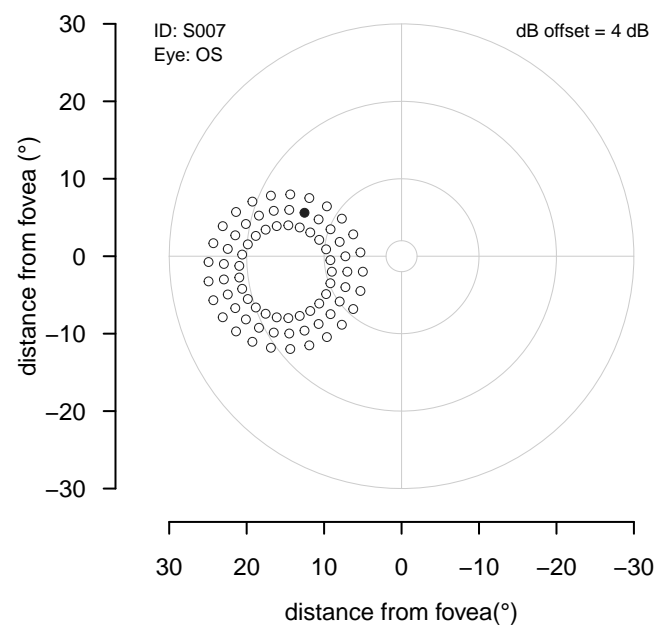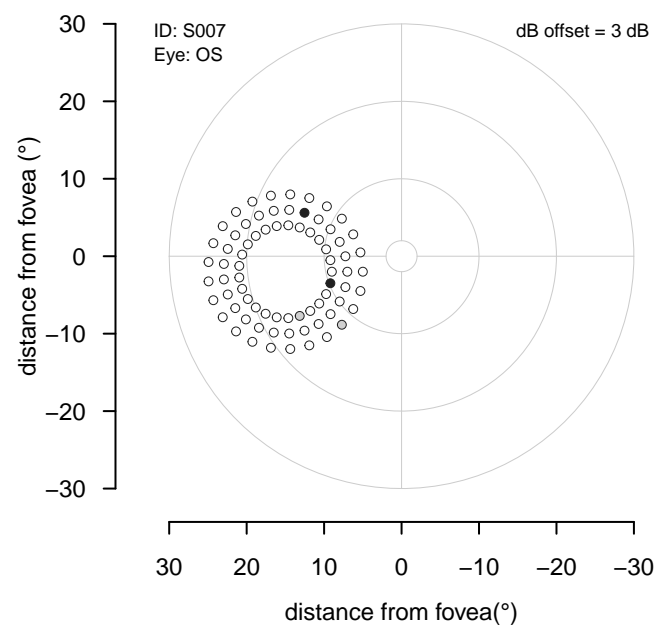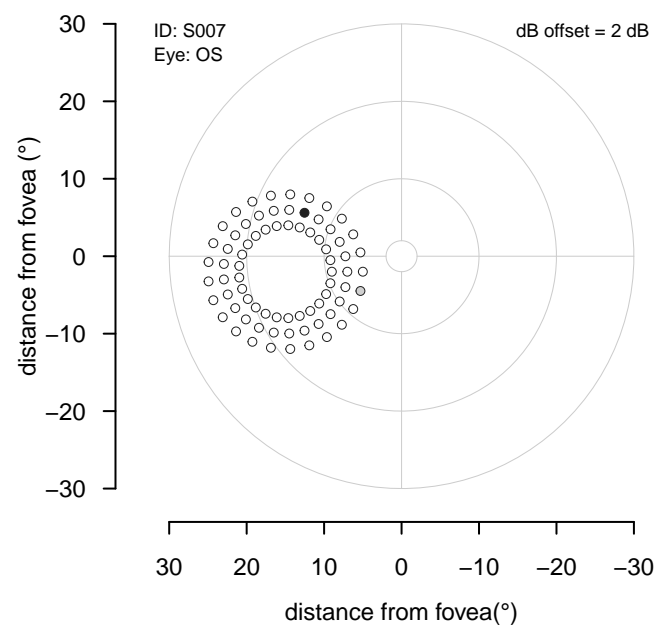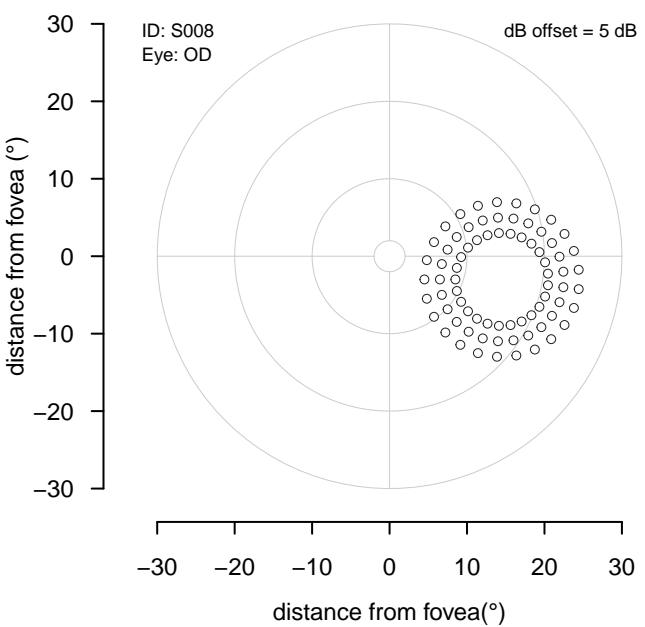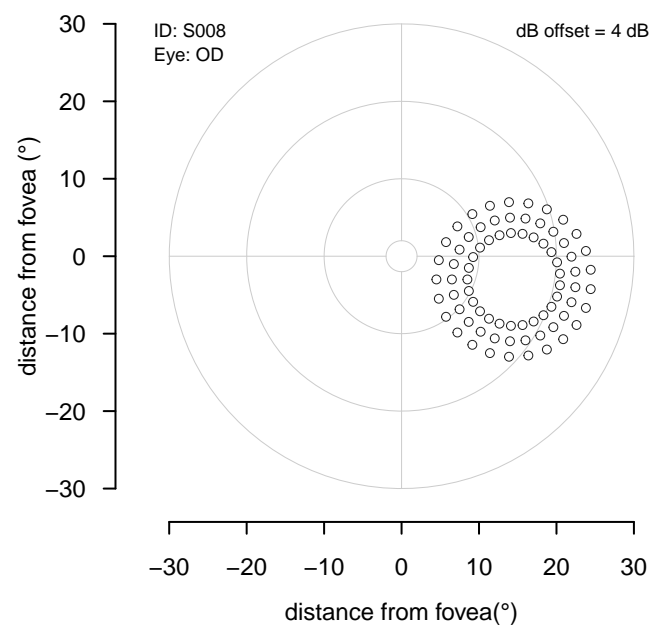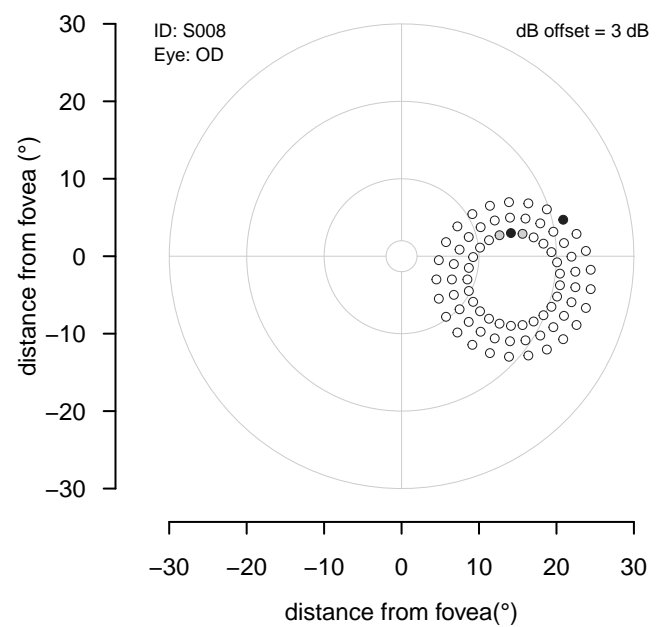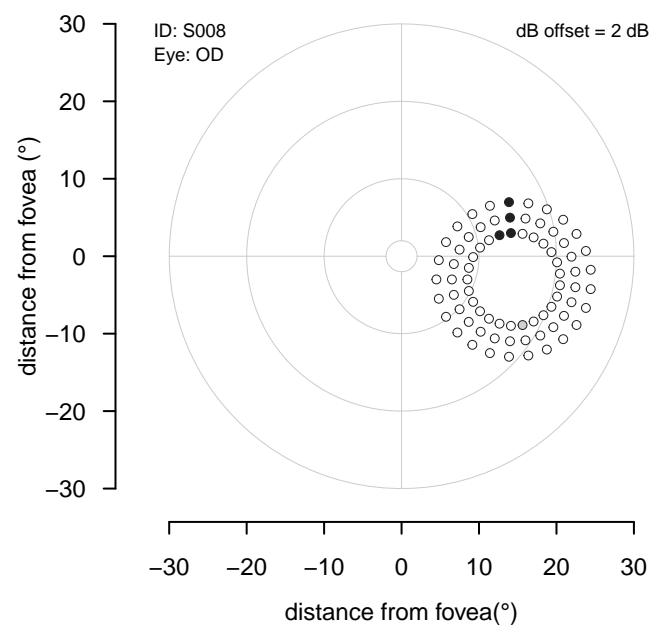

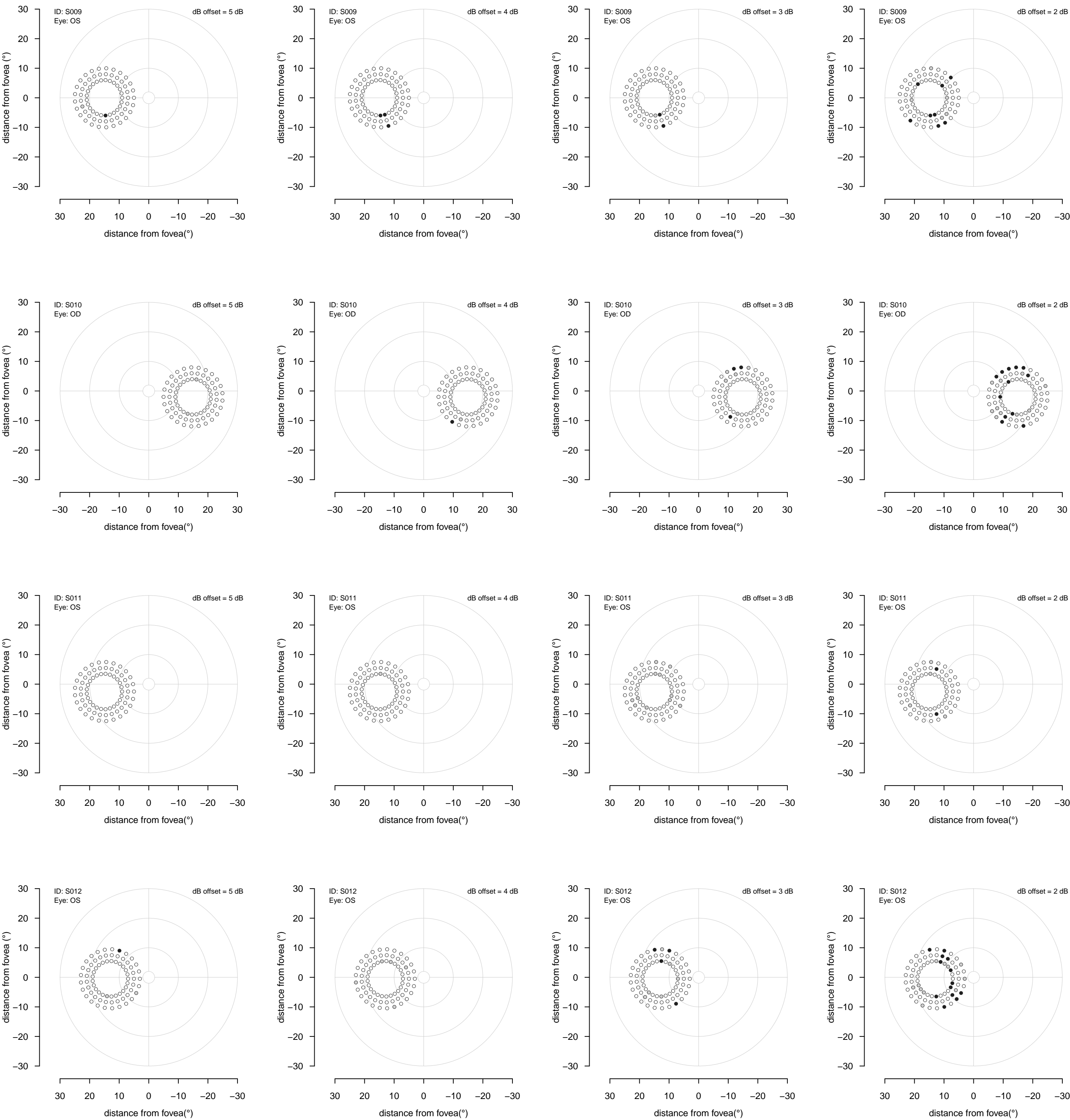

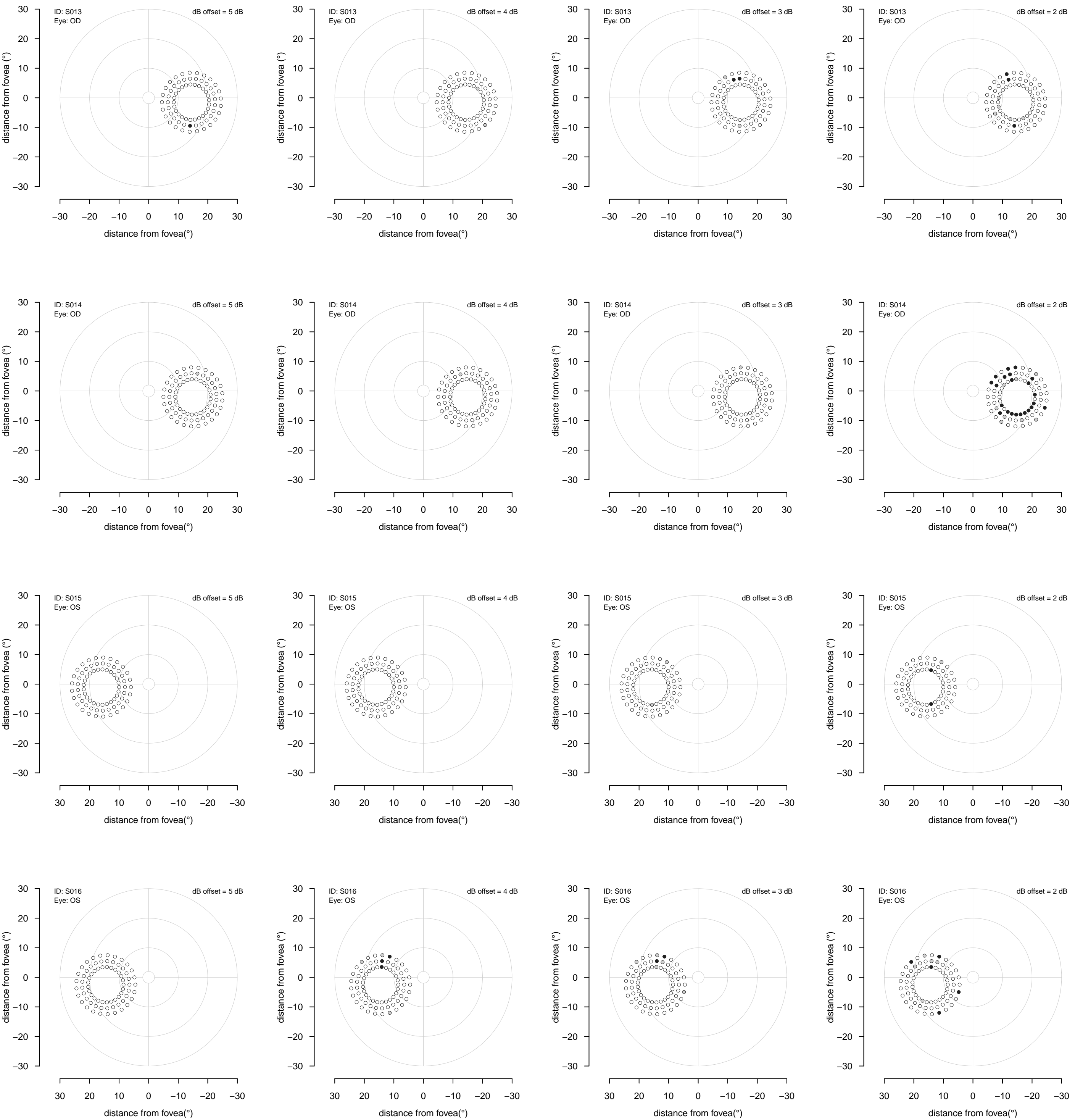

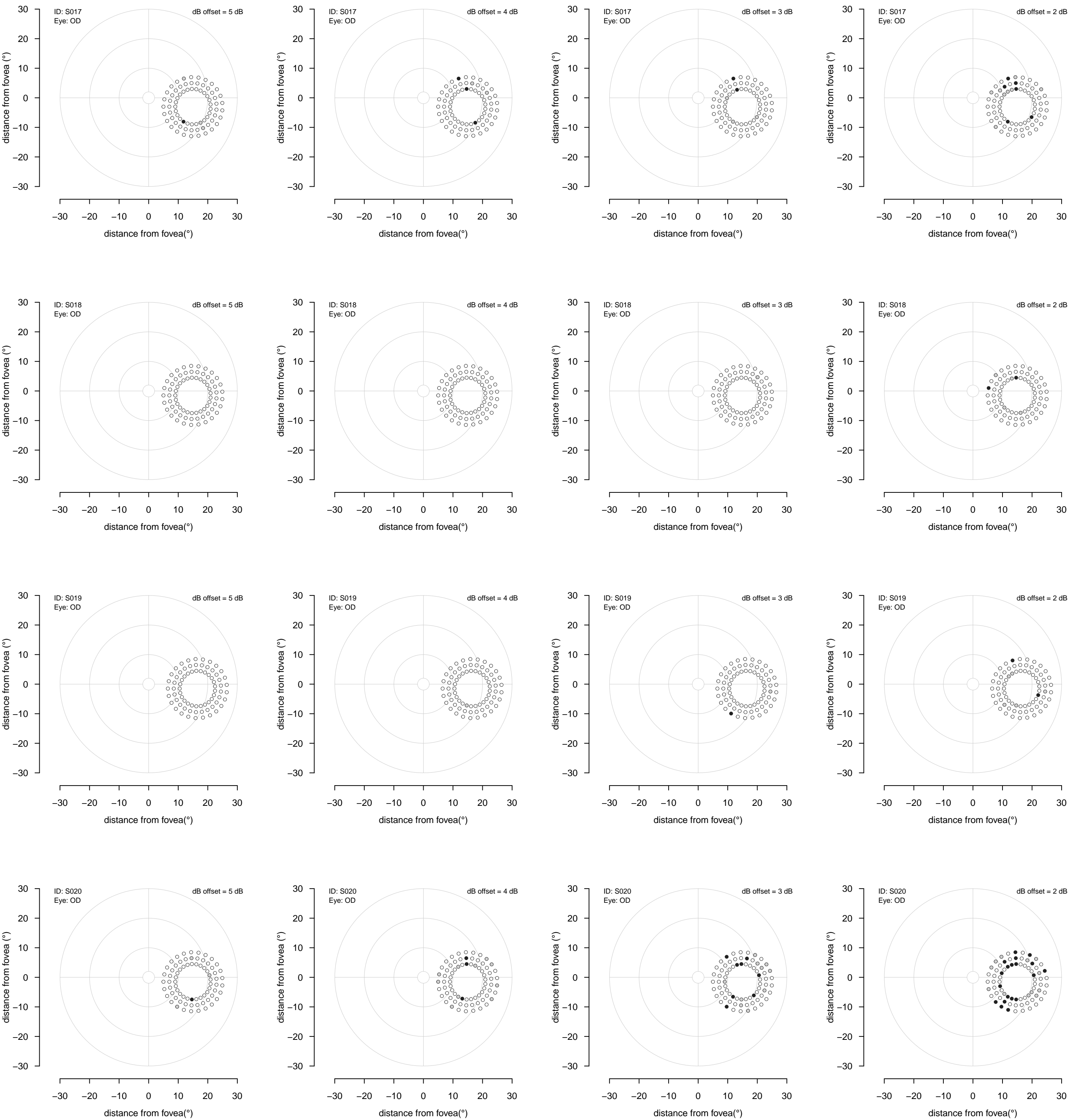

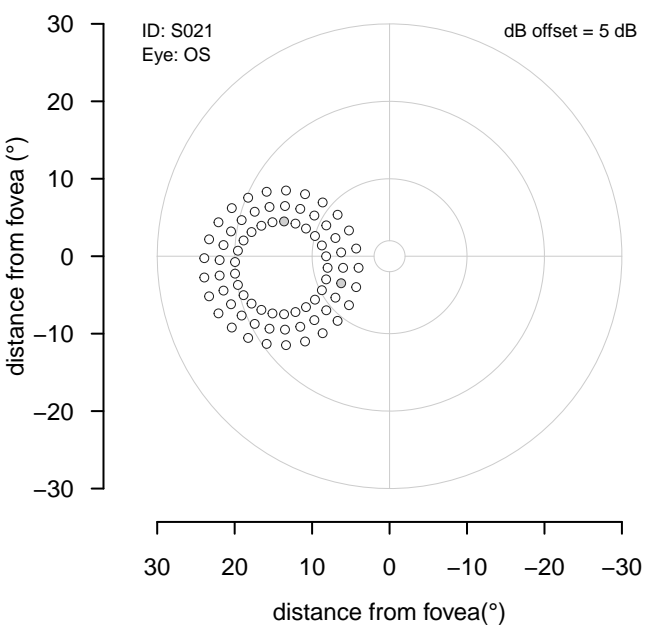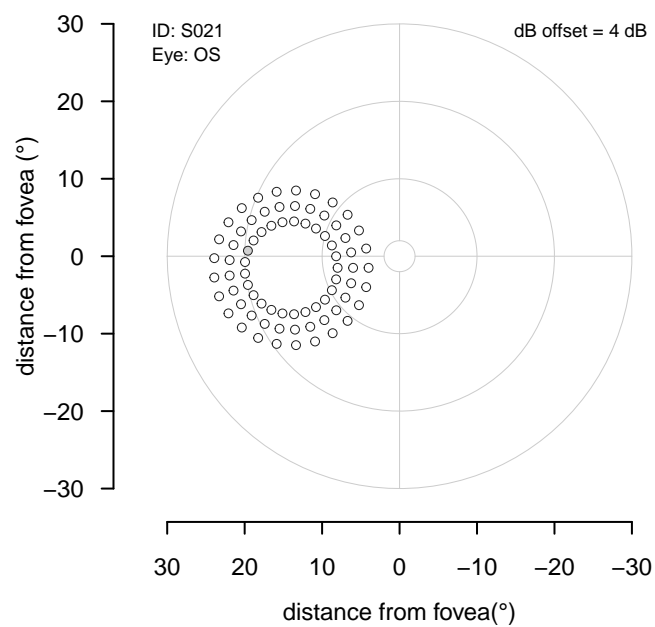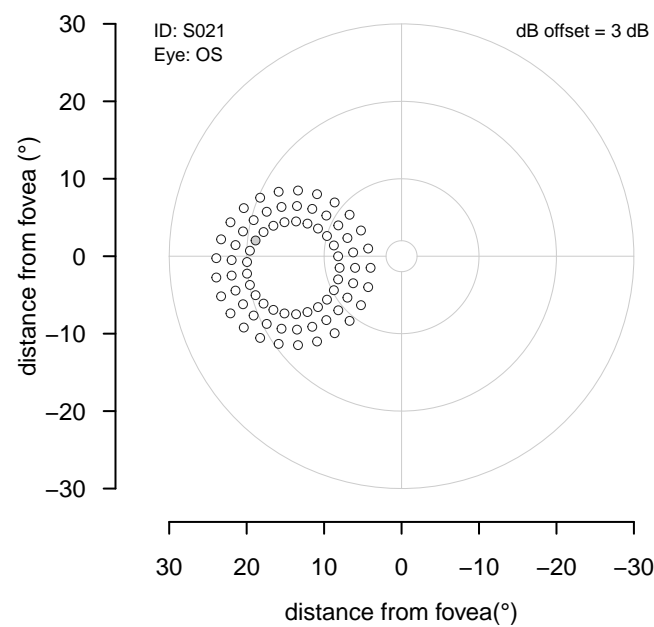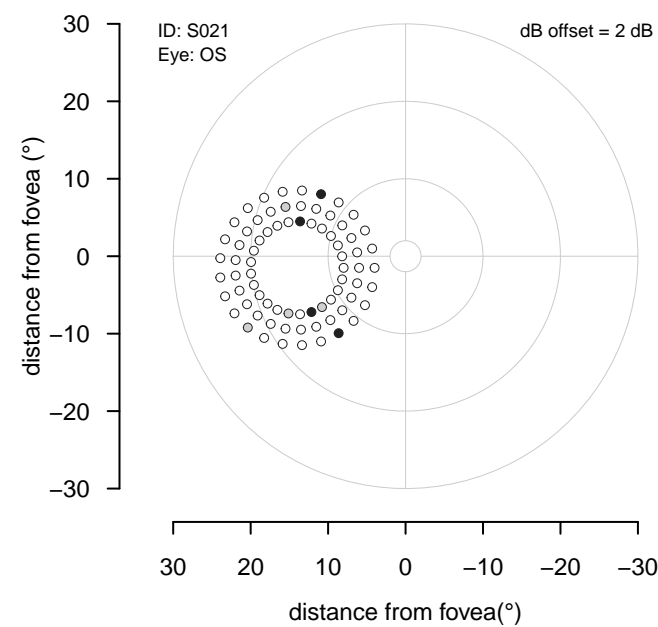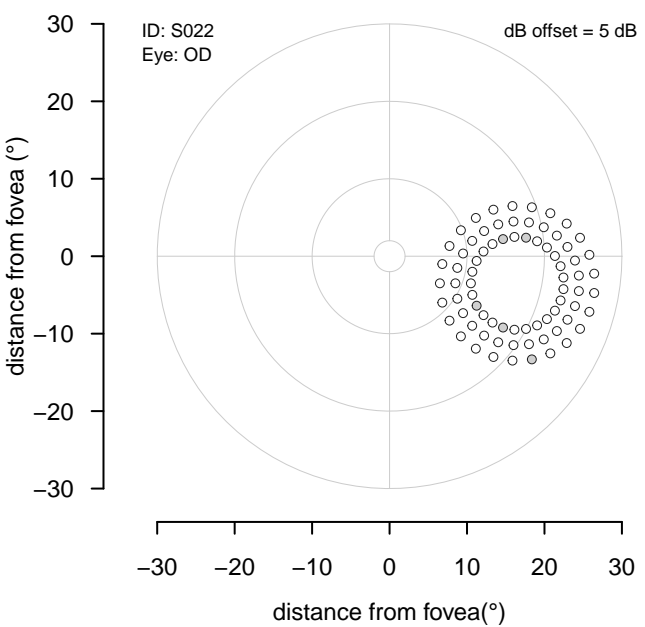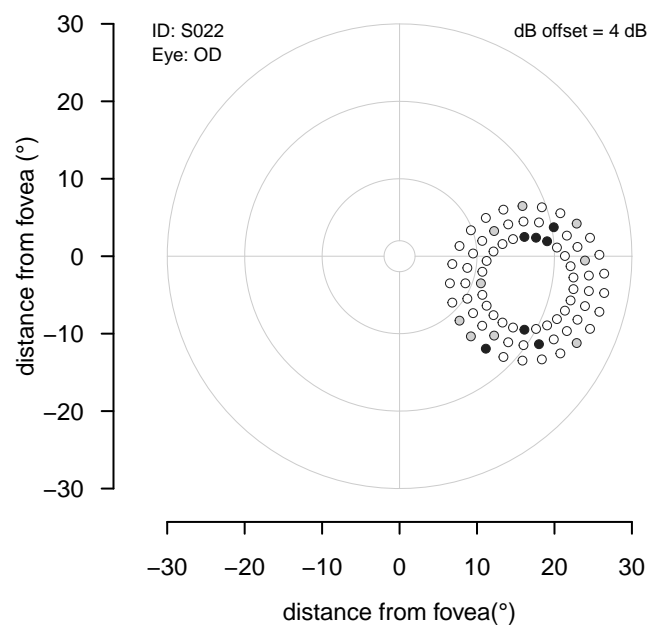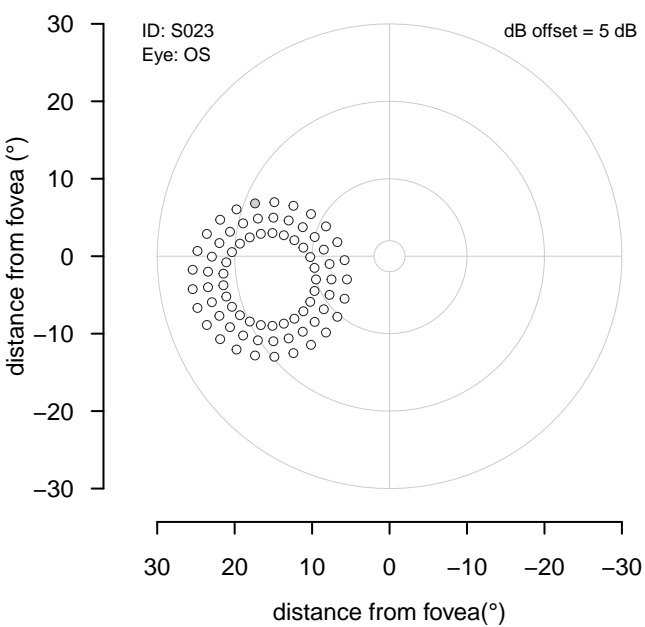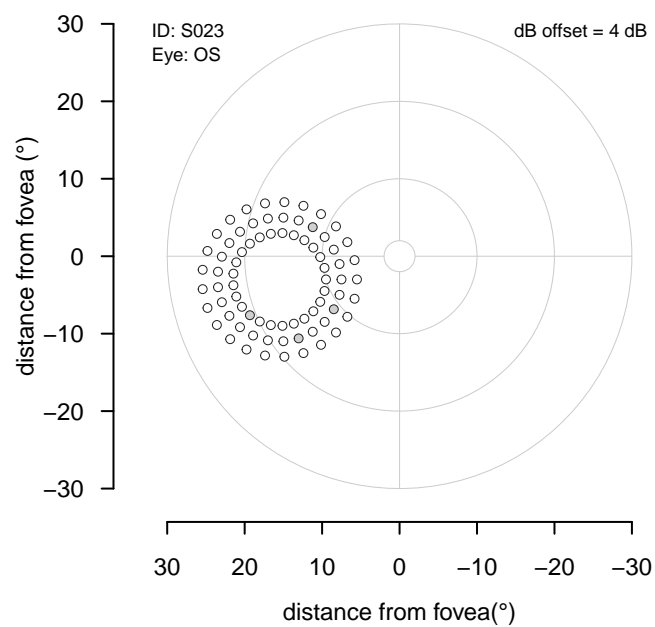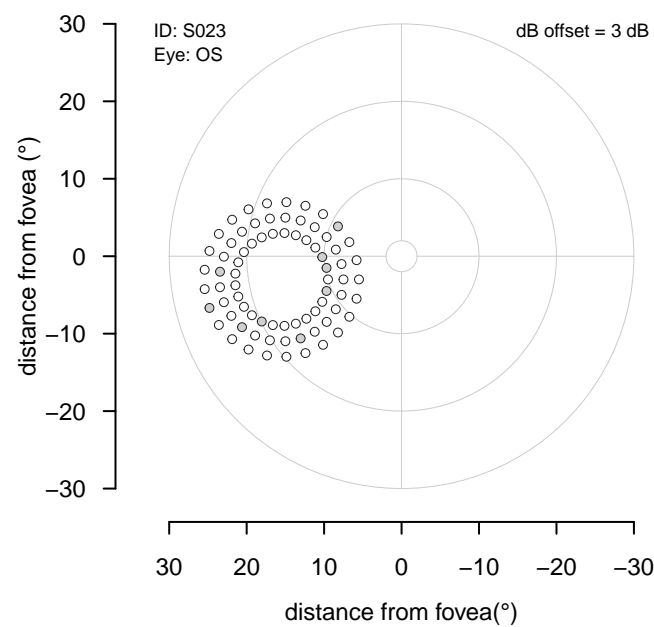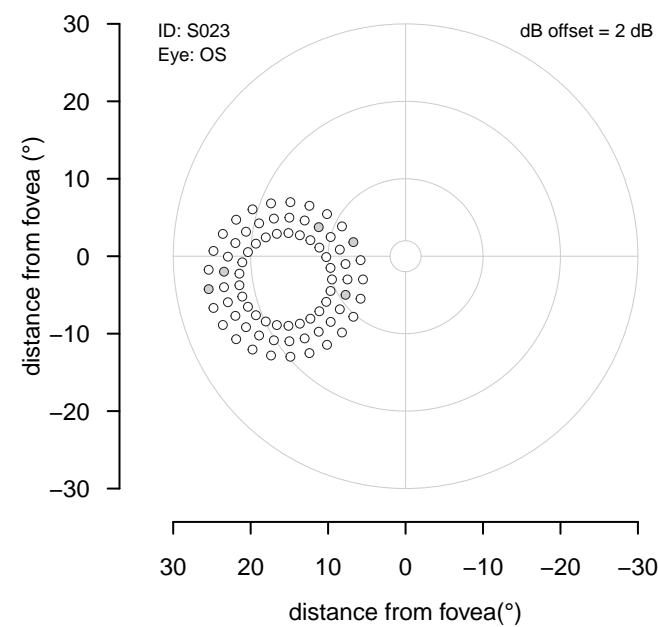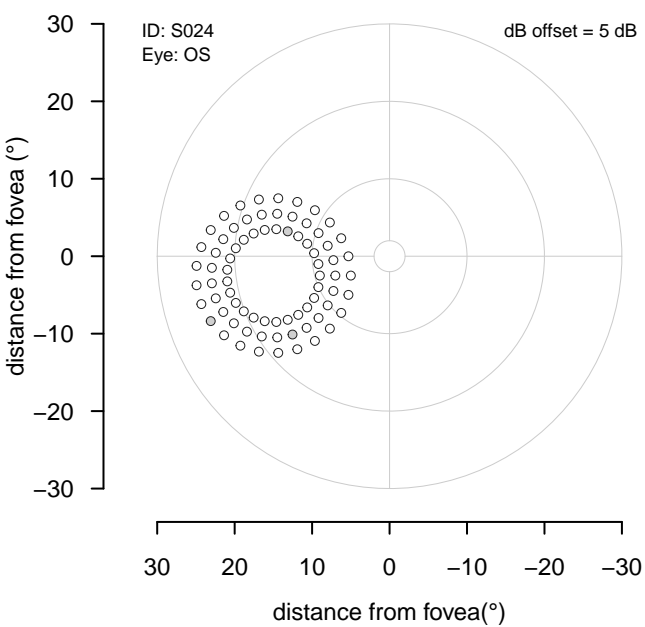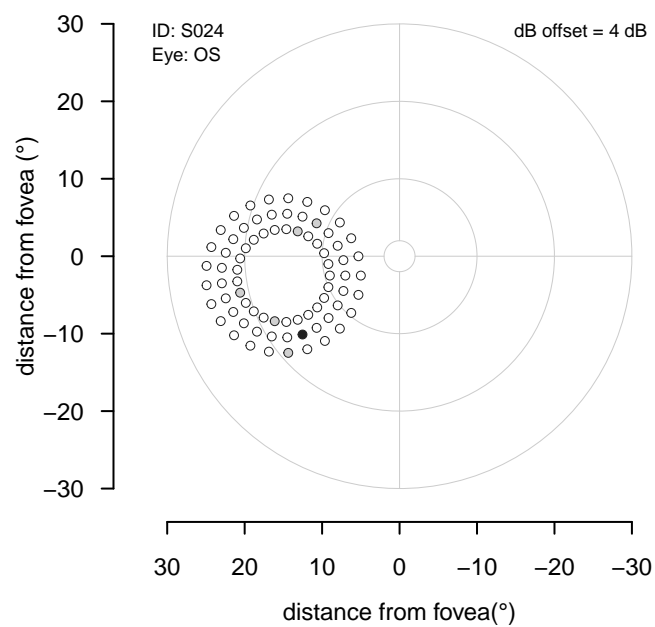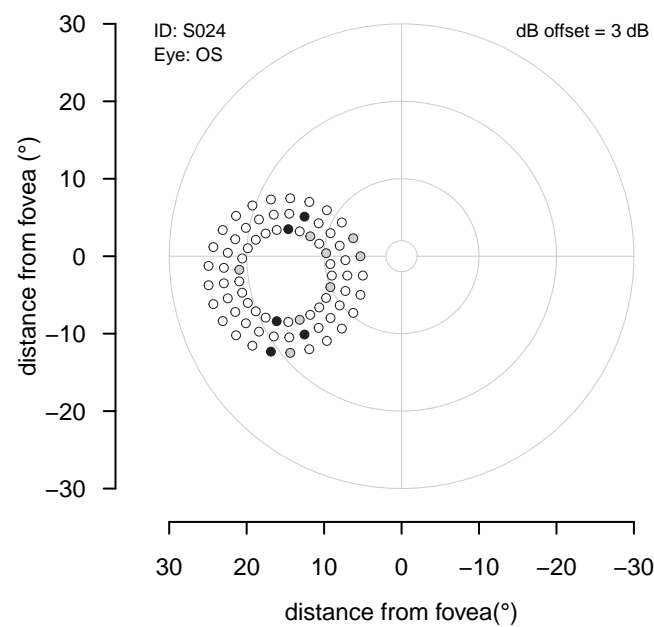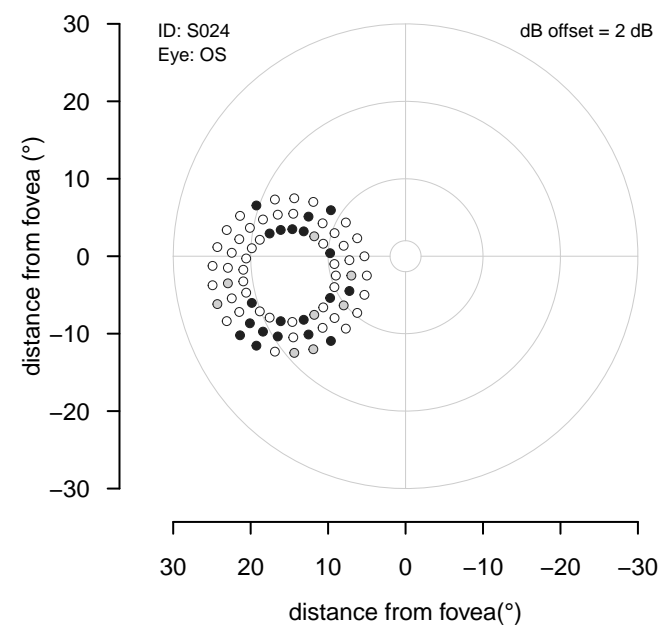

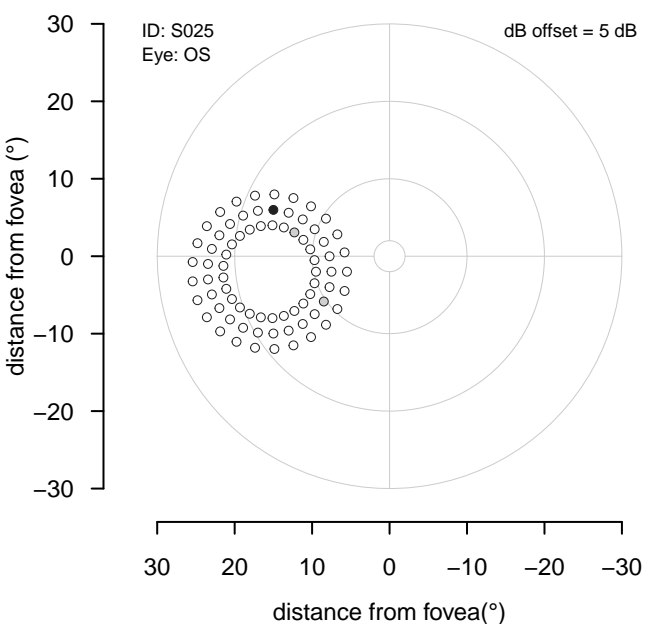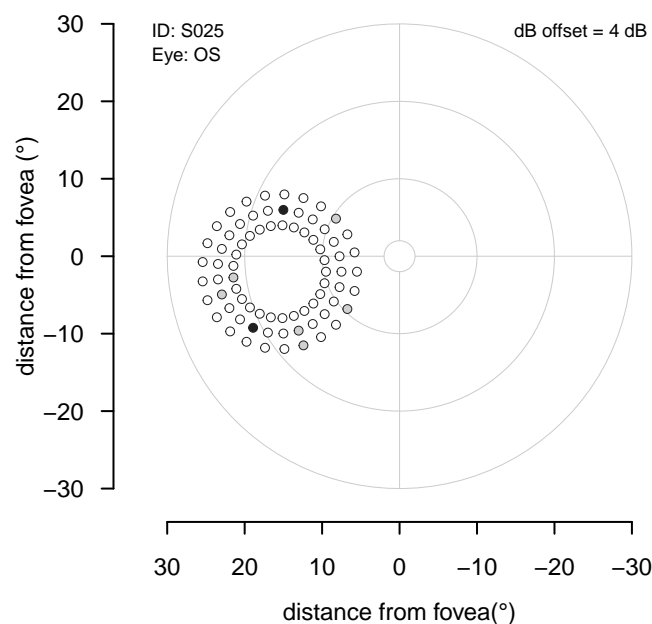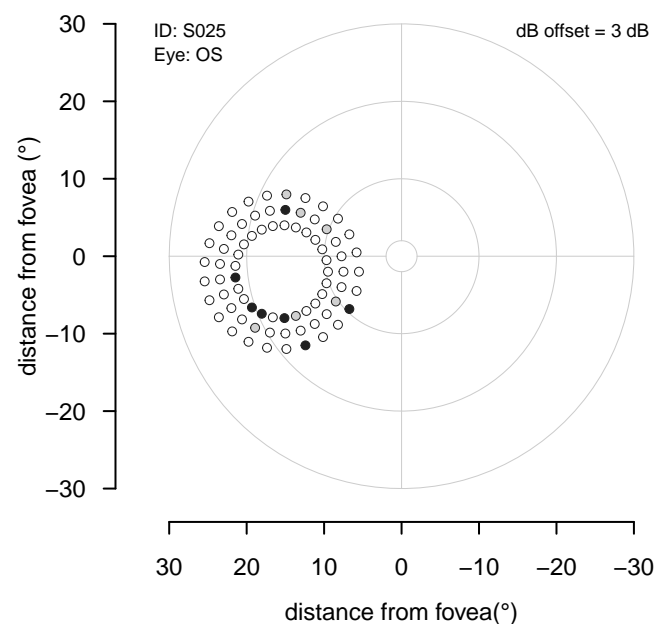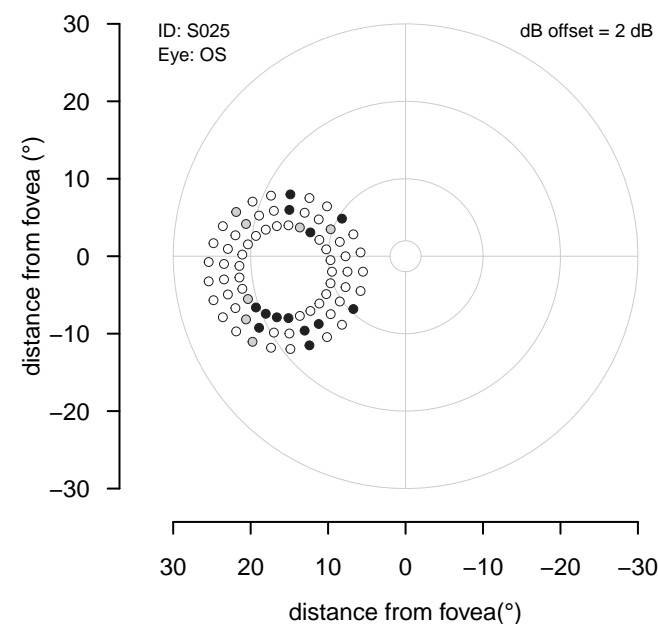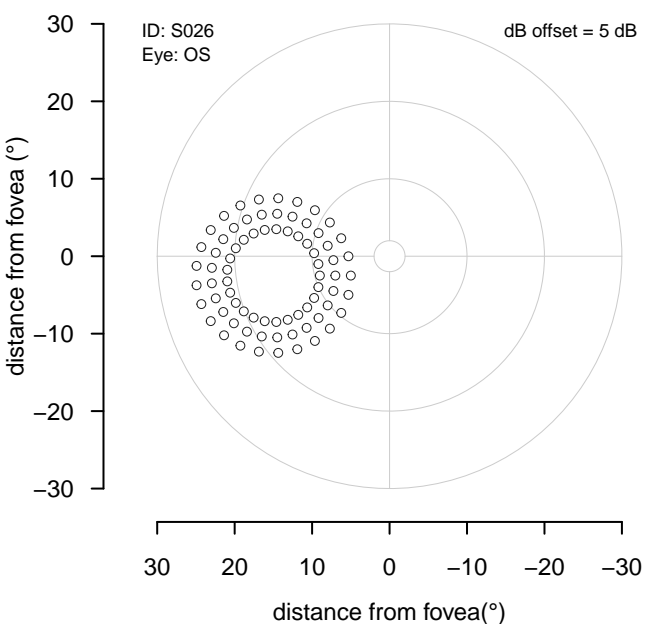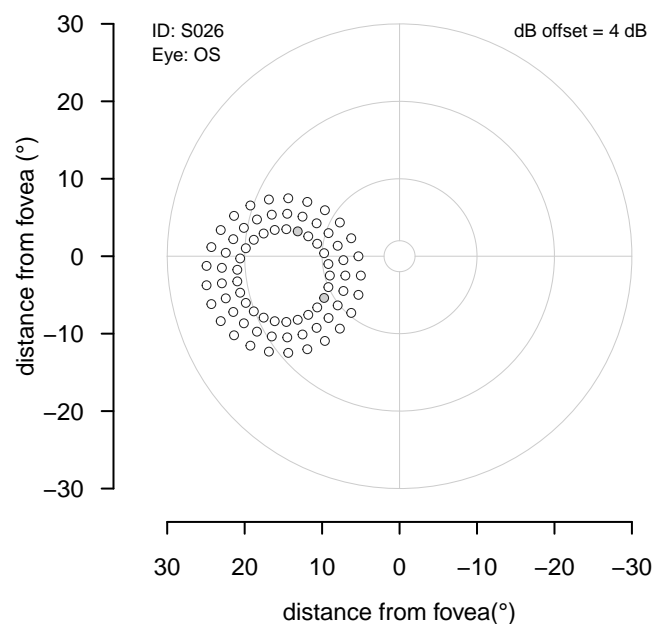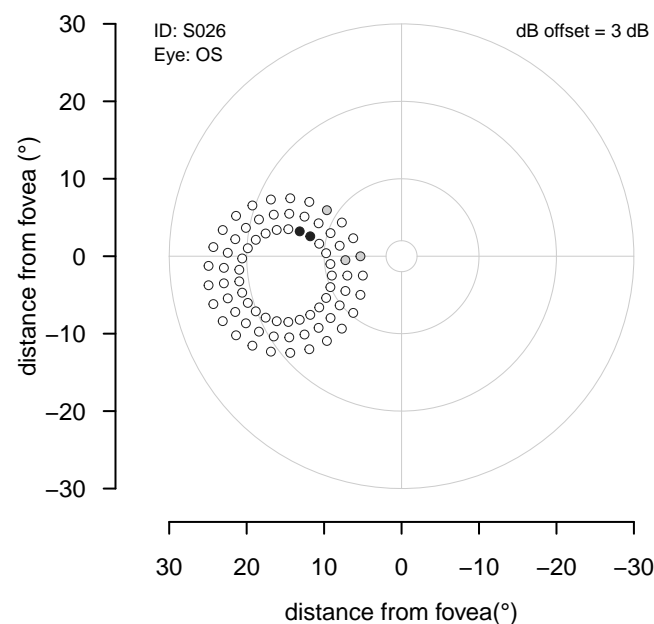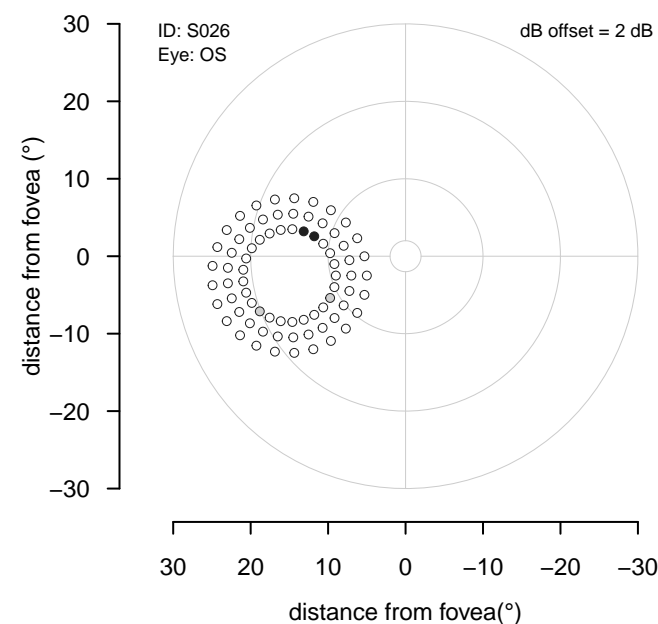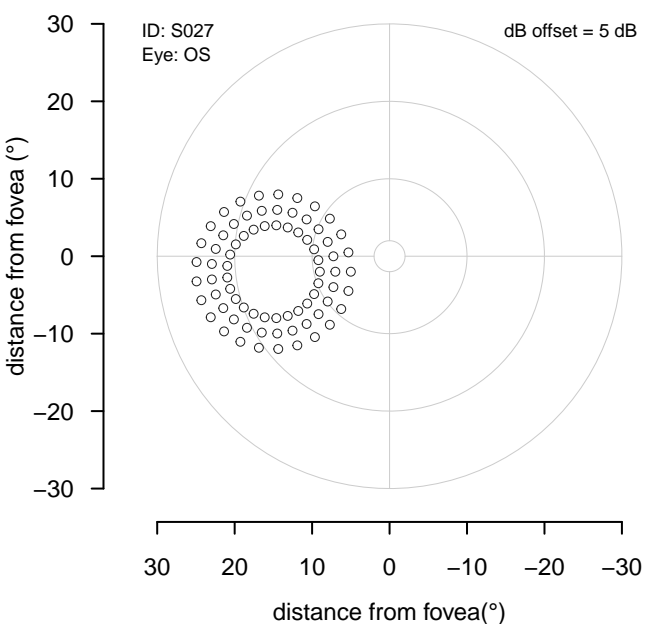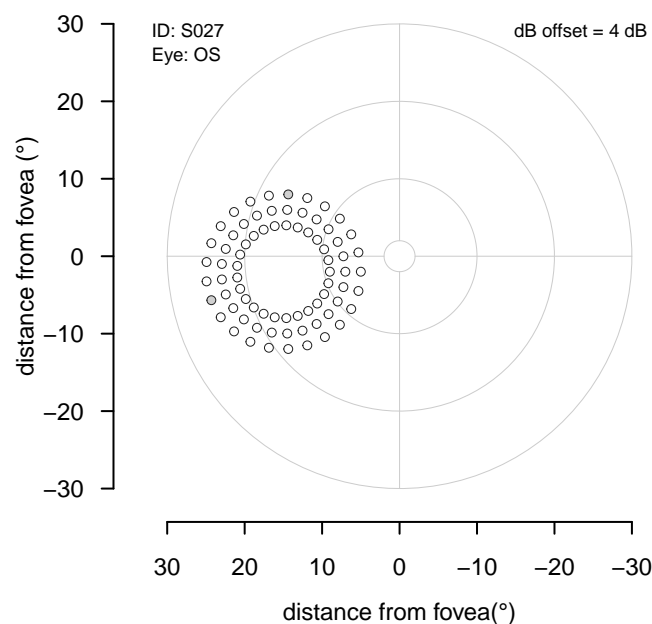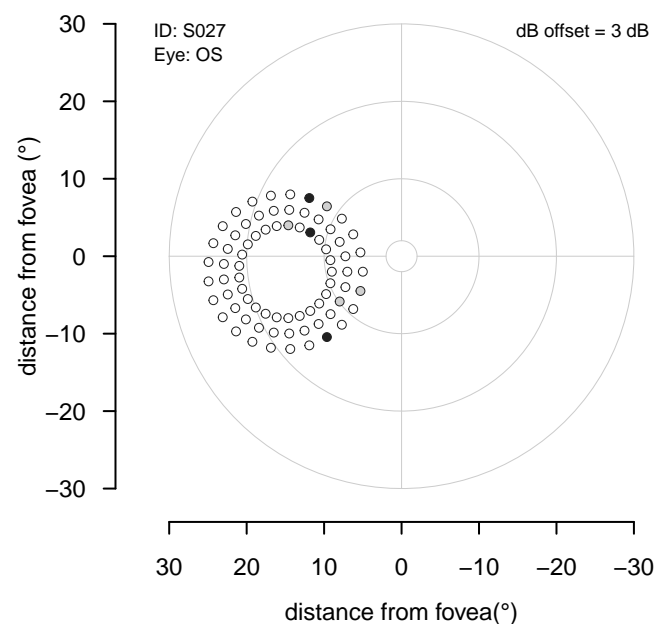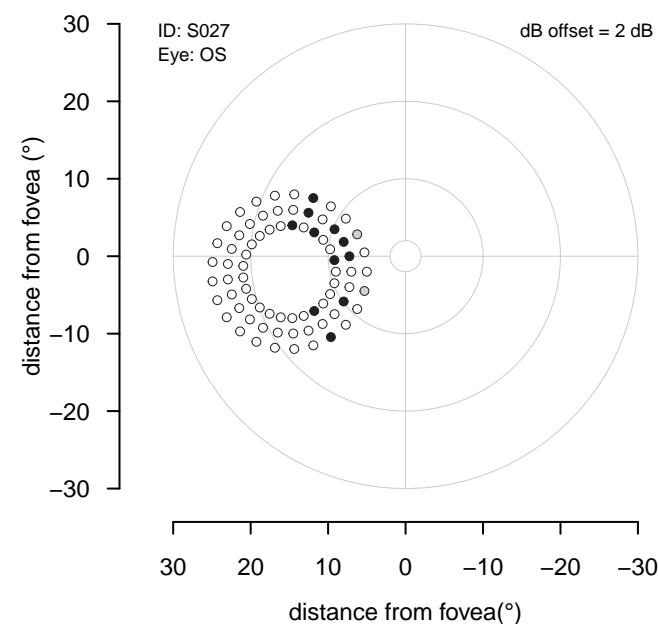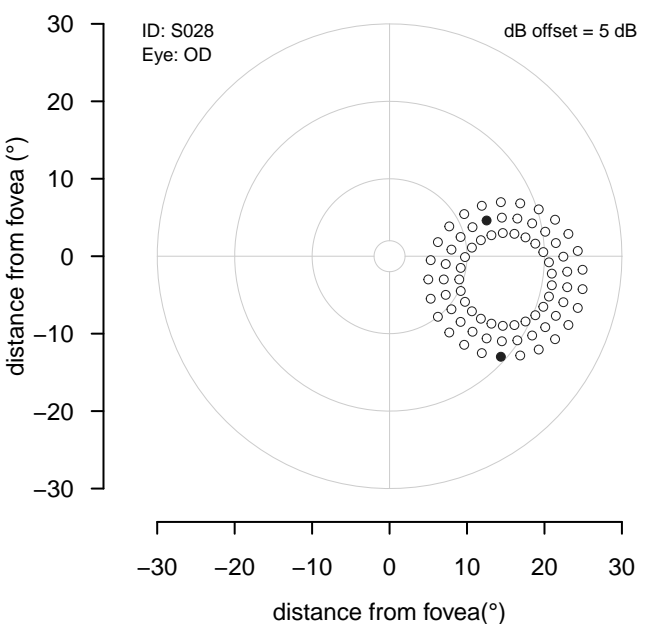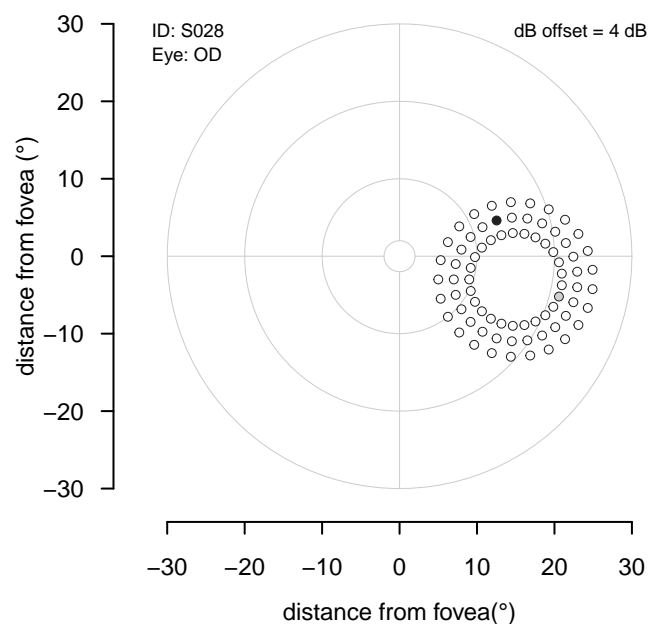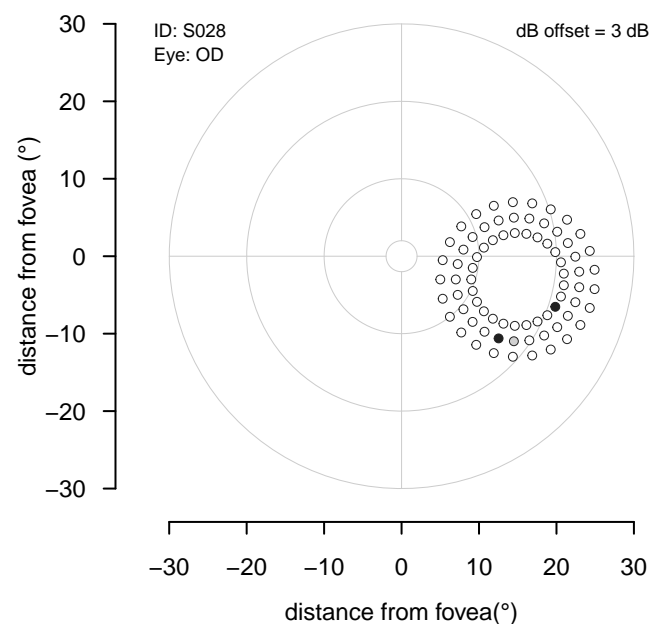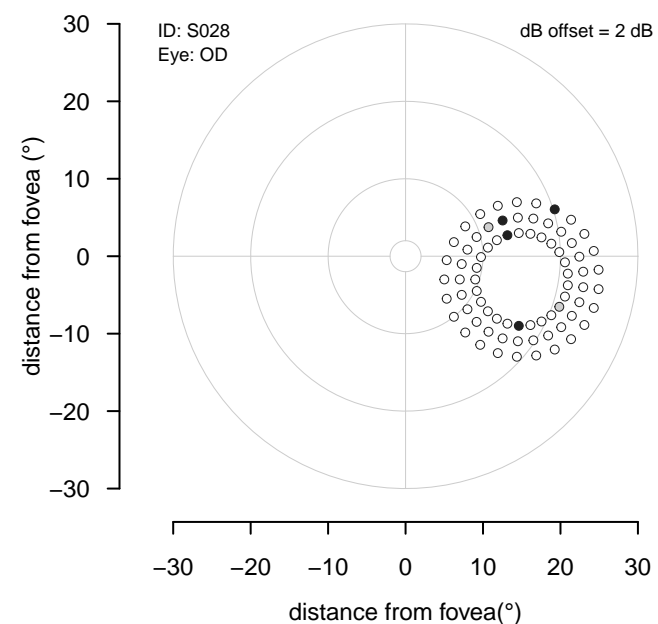

Supplement: Supplementary file 1 — Appendix S1. (ZIP 3.30 MB) [file 44402_2025_4507042_MOESM1_ESM.zip › opo70014-sup-0003-Supinfo3.pdf]

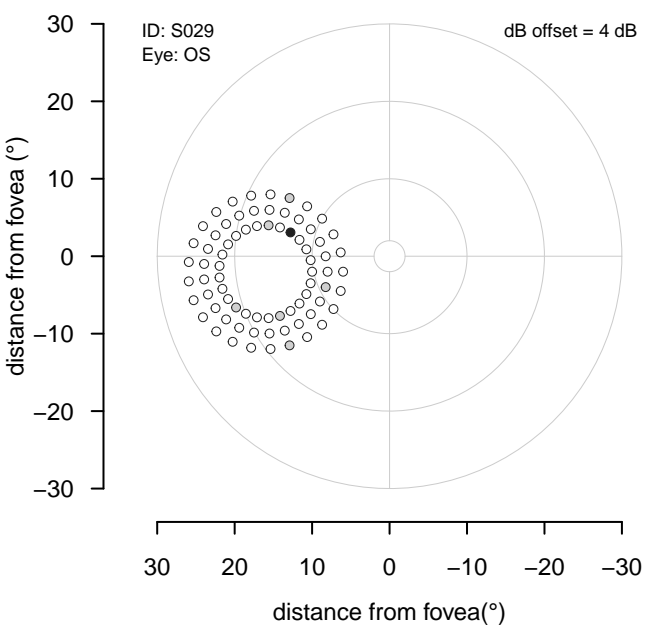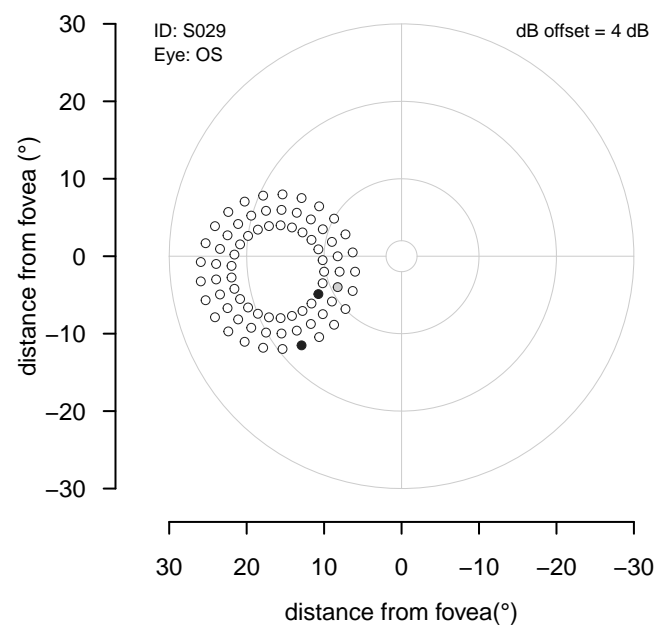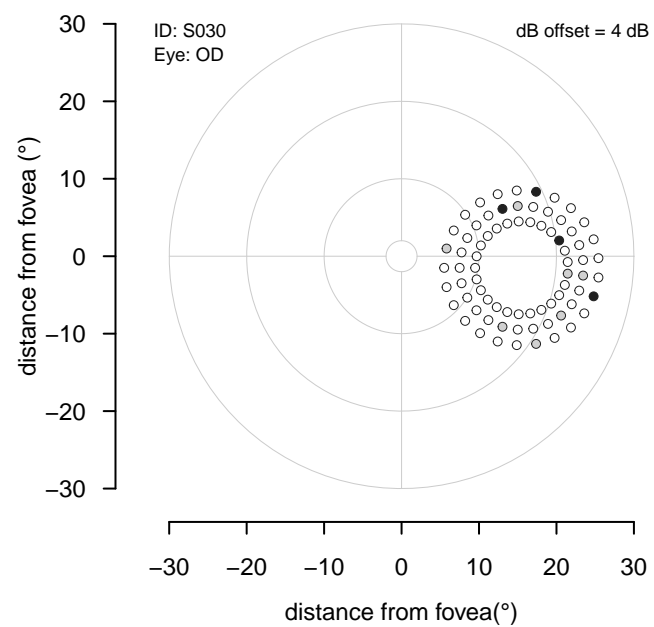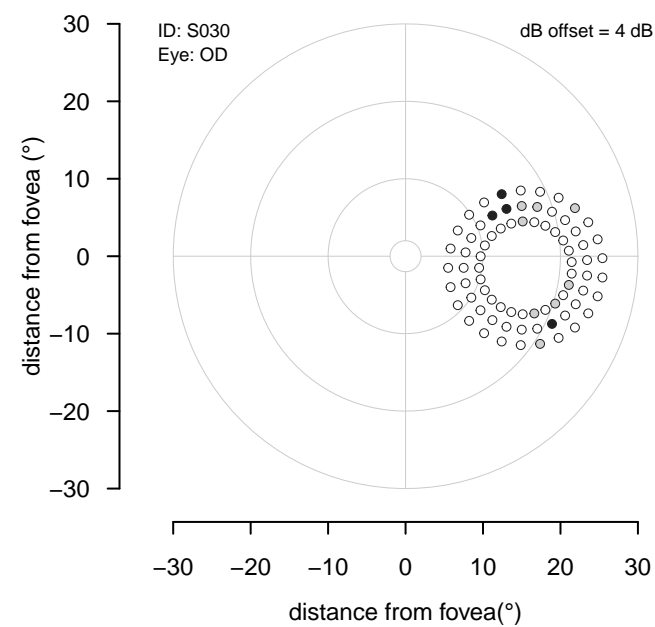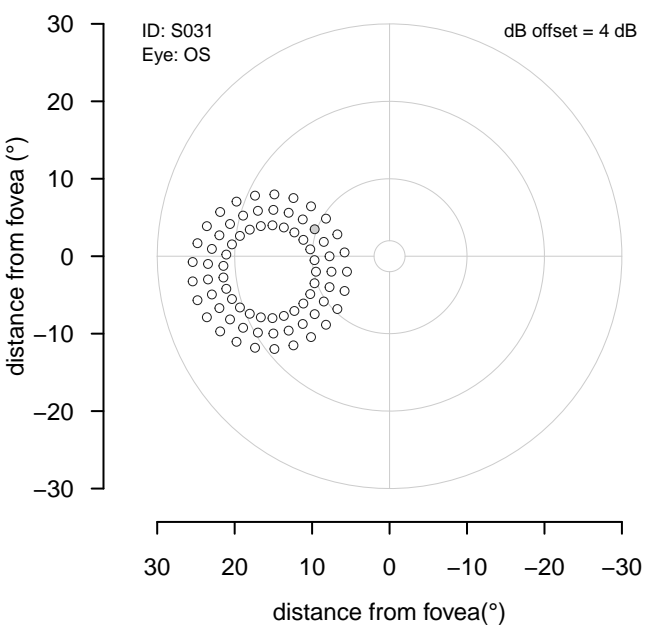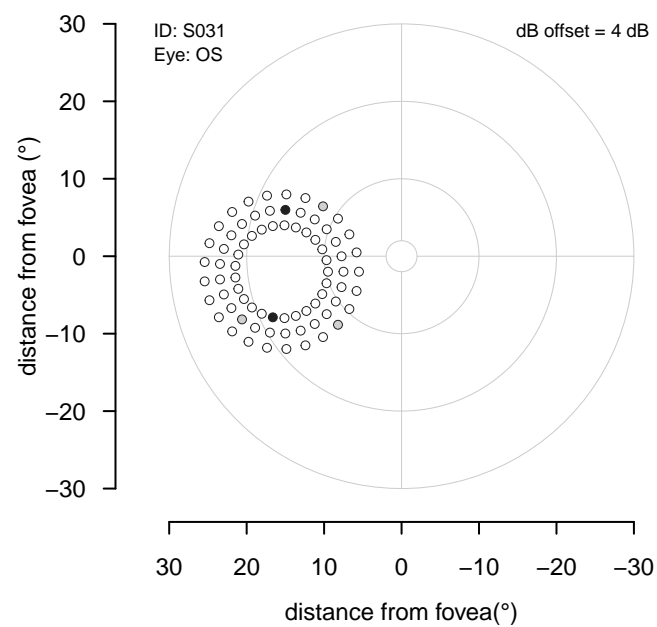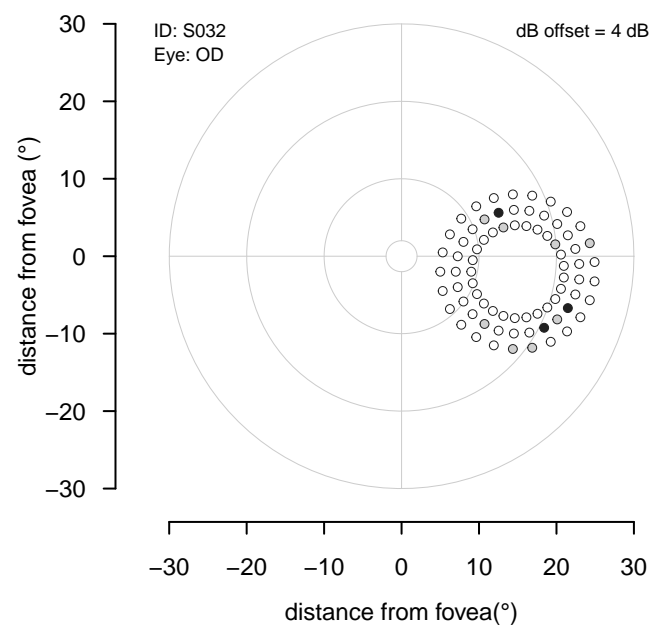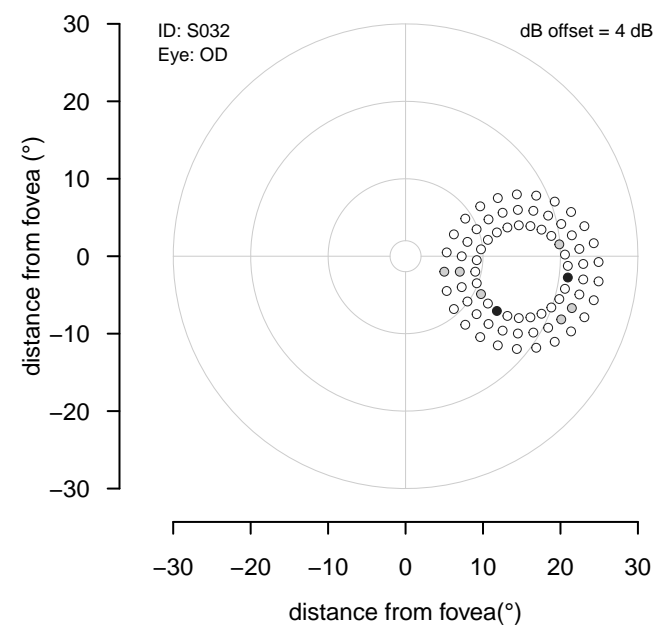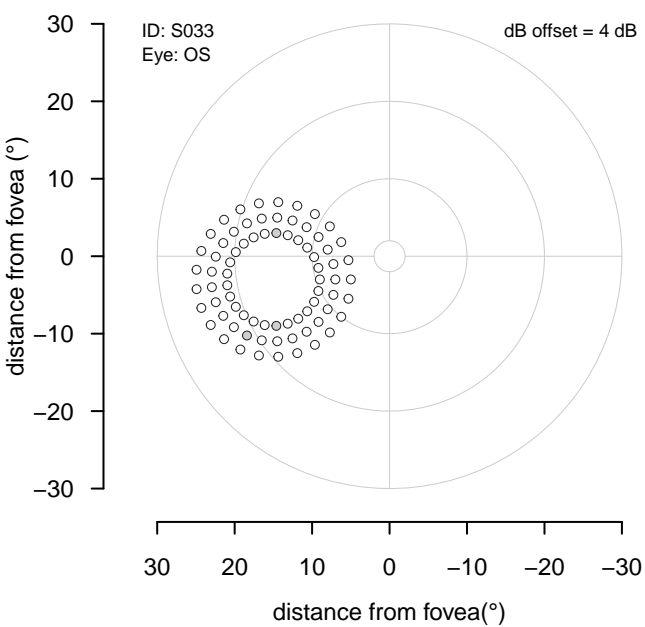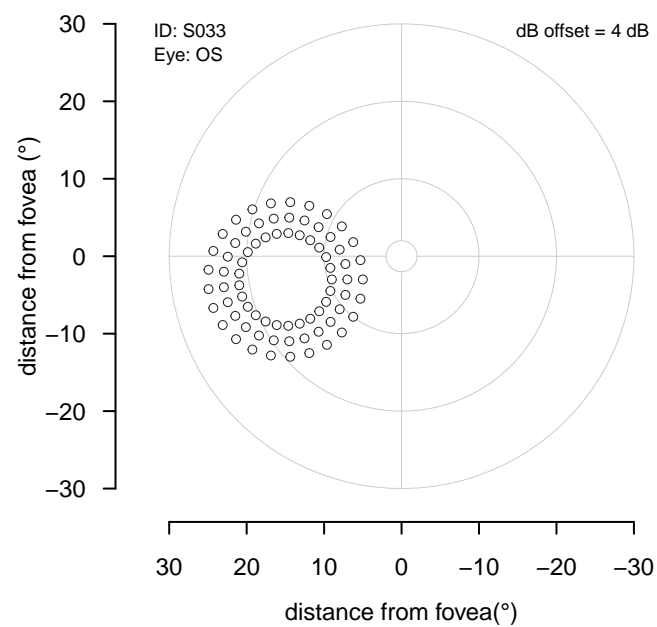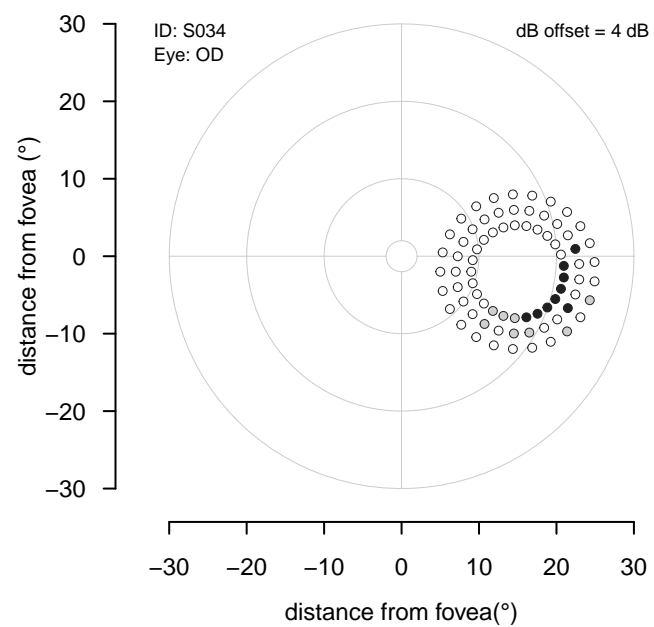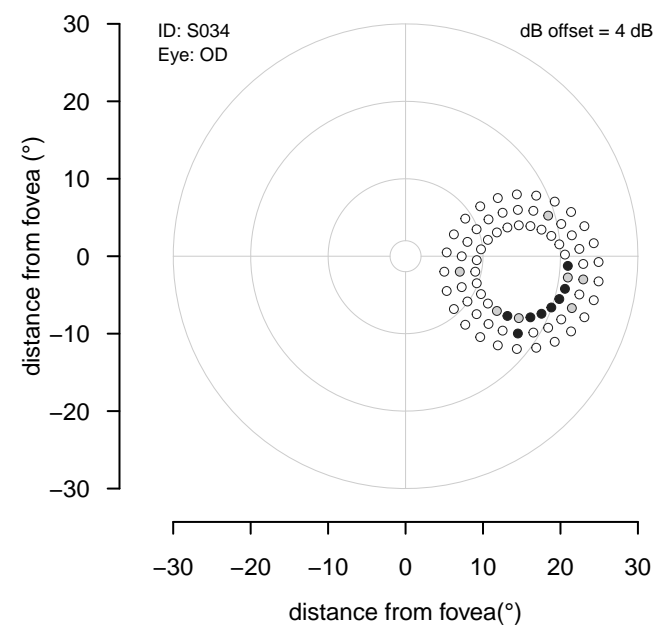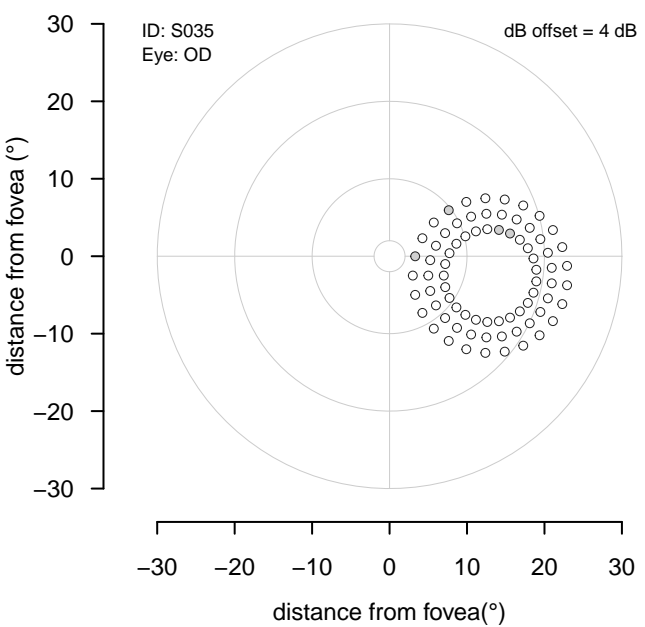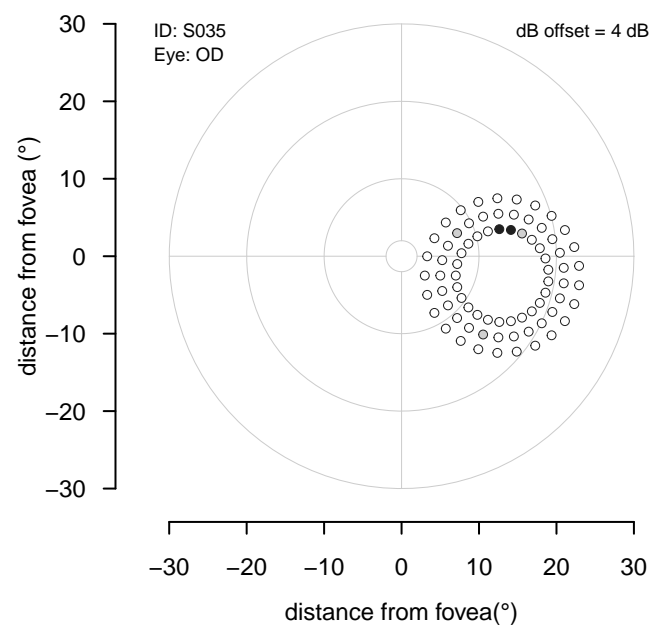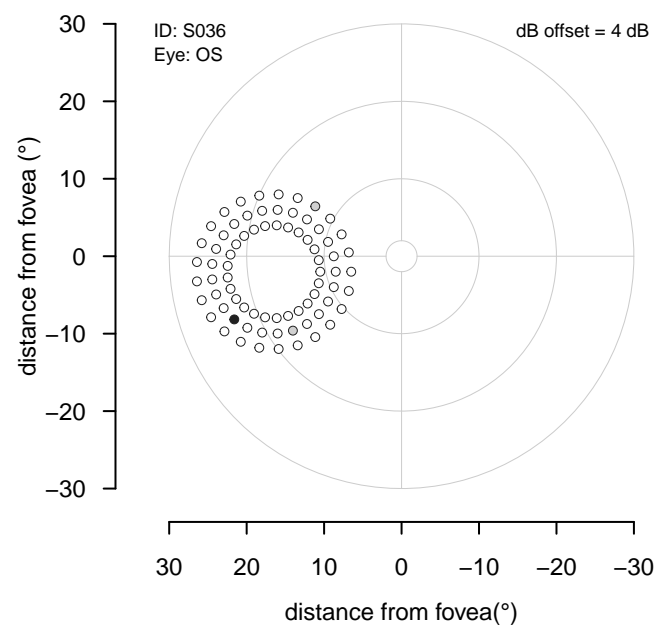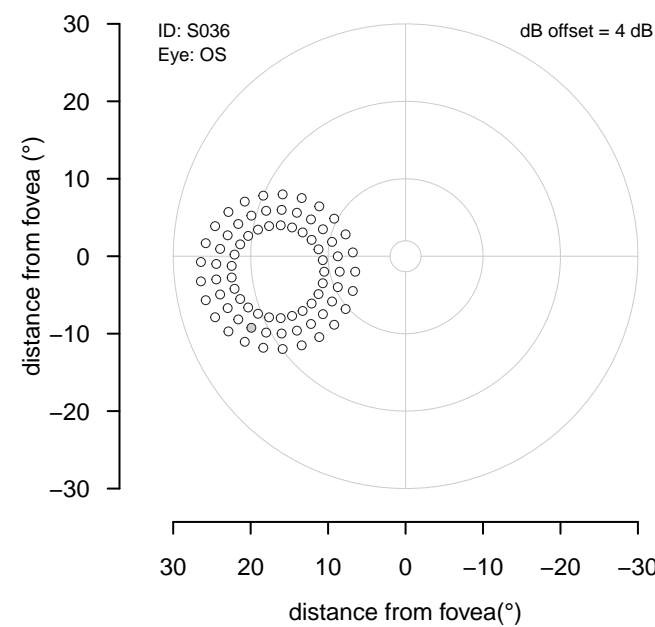

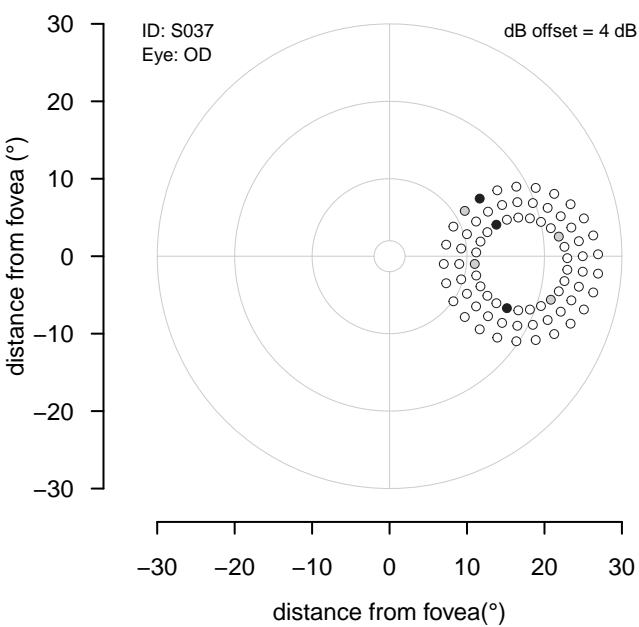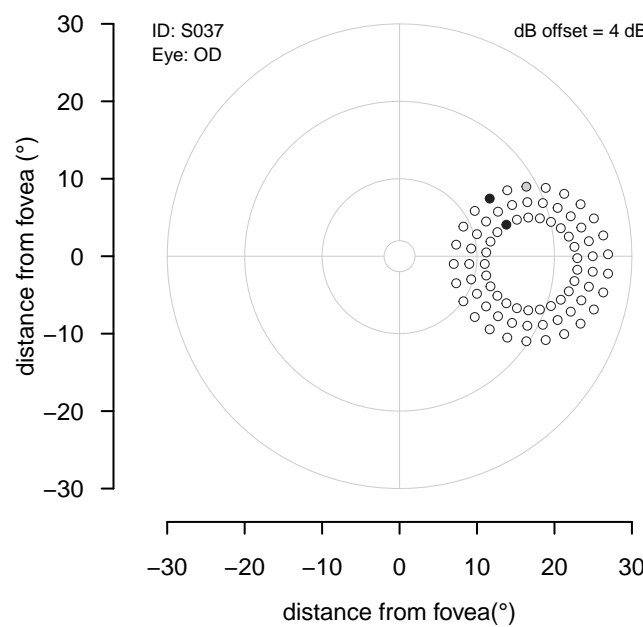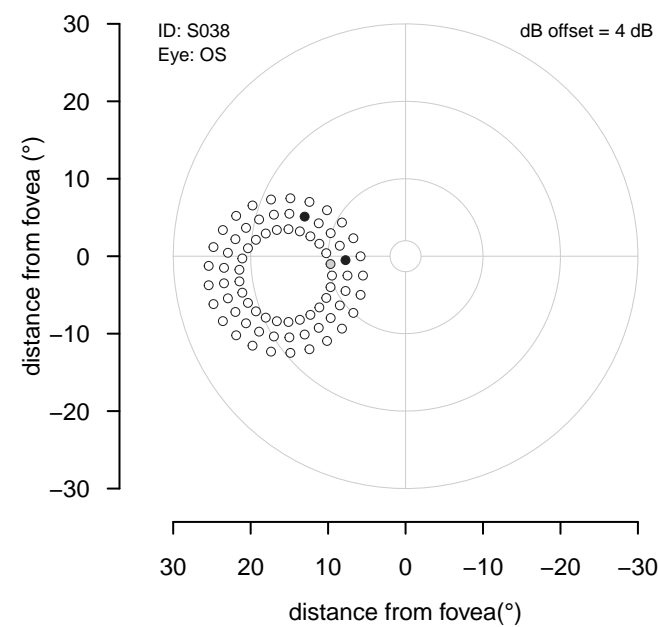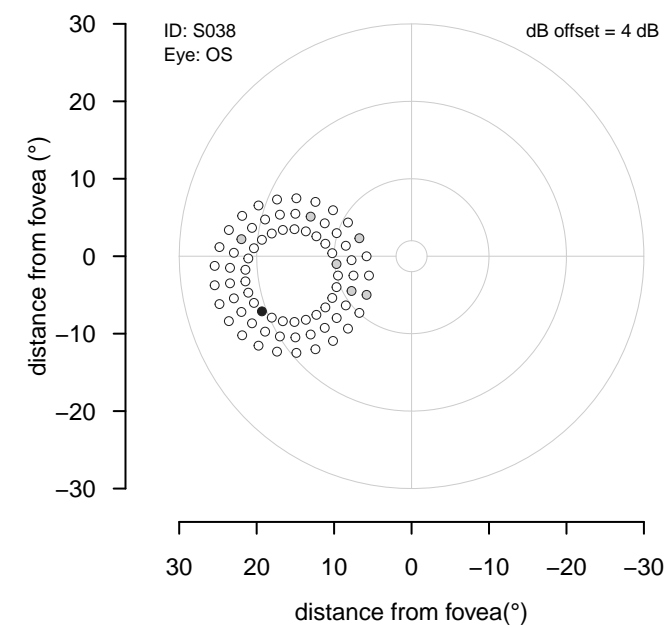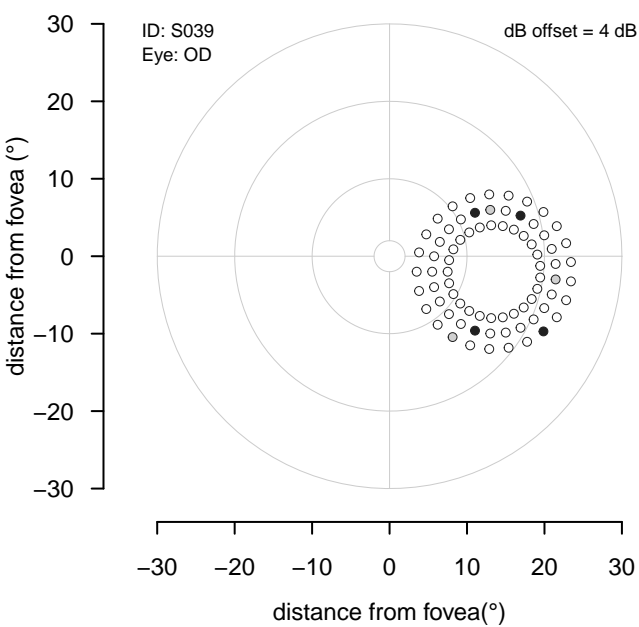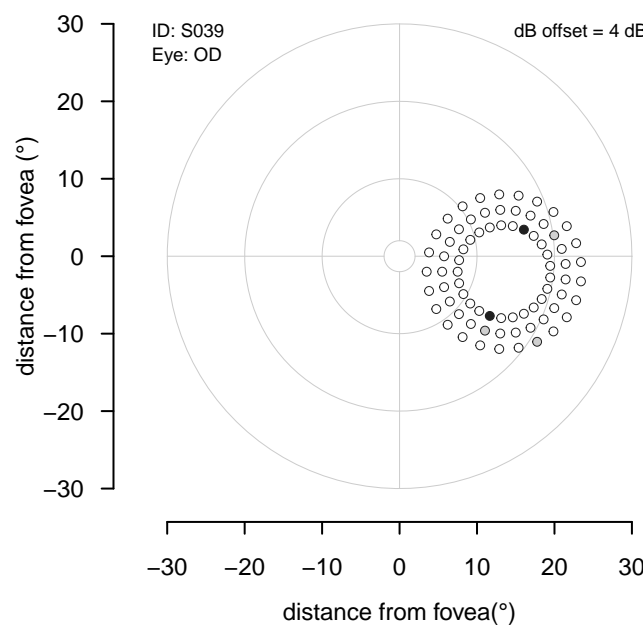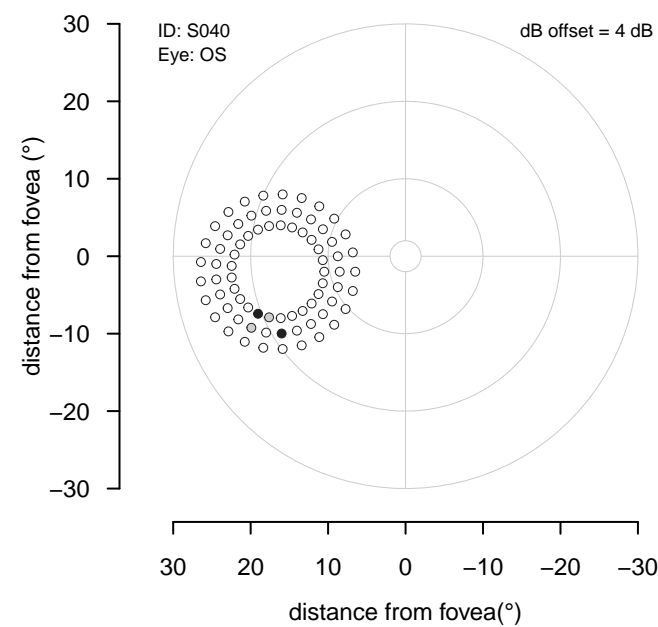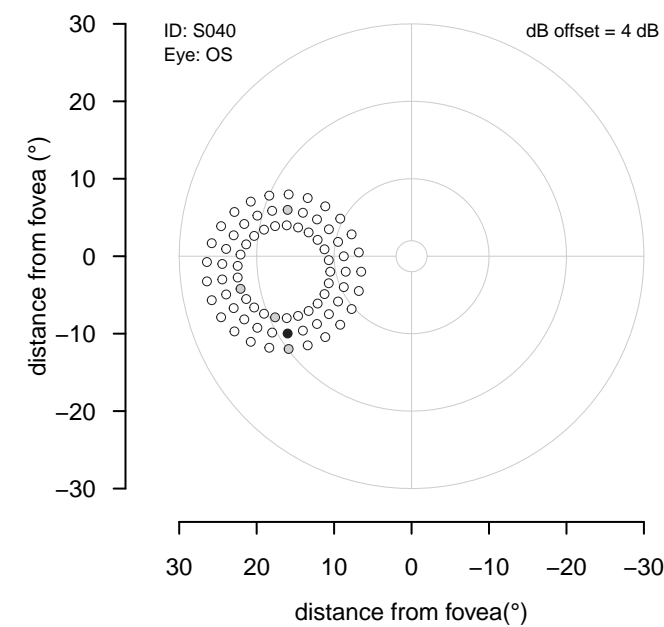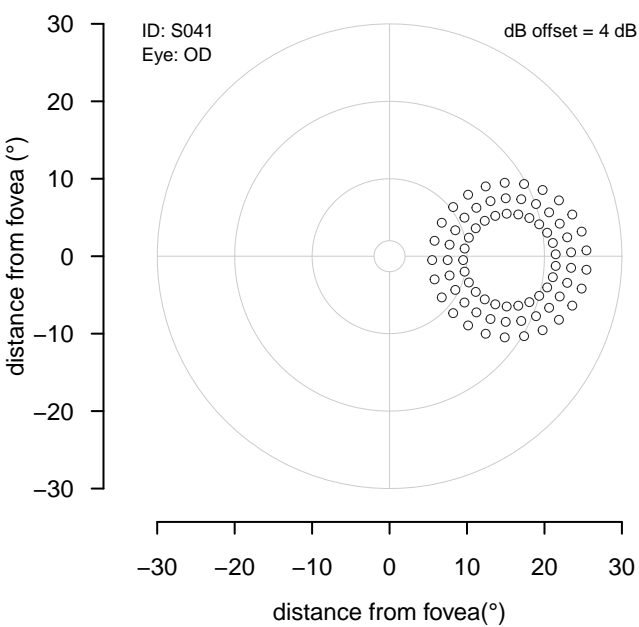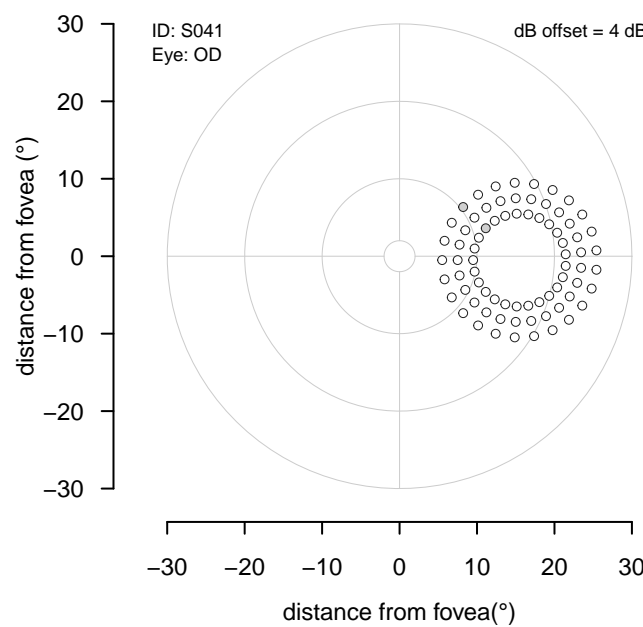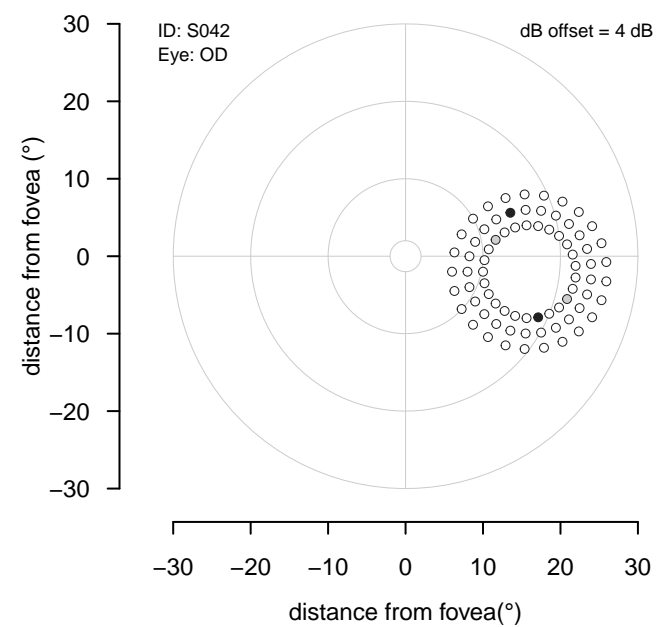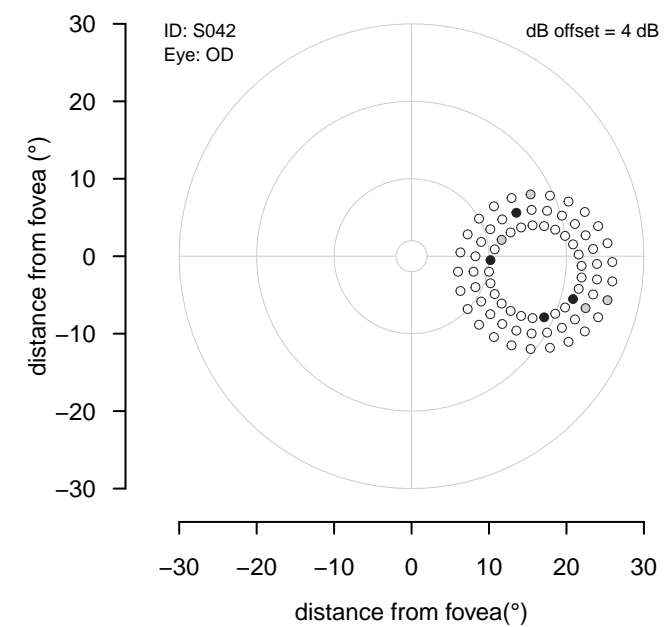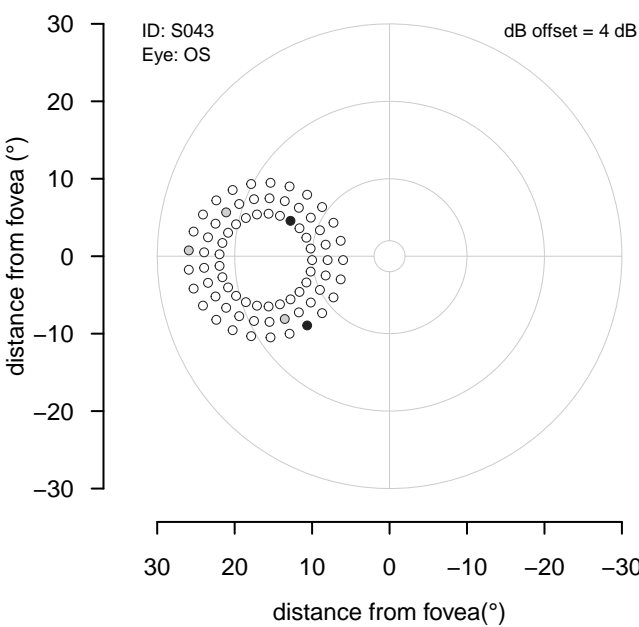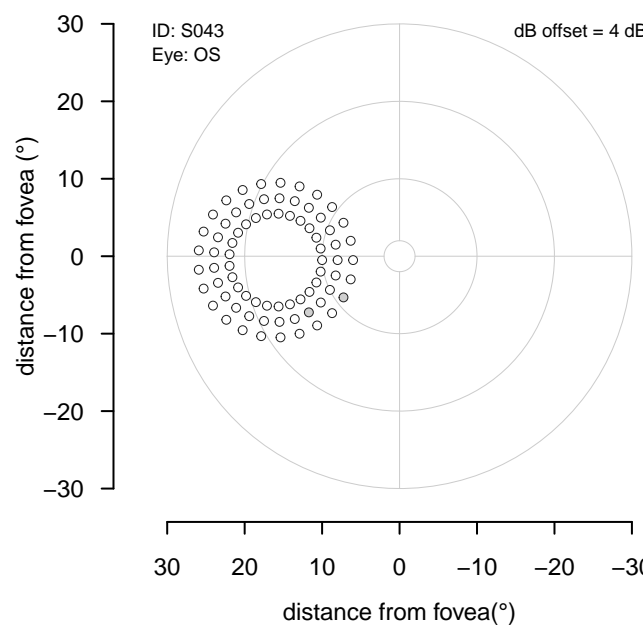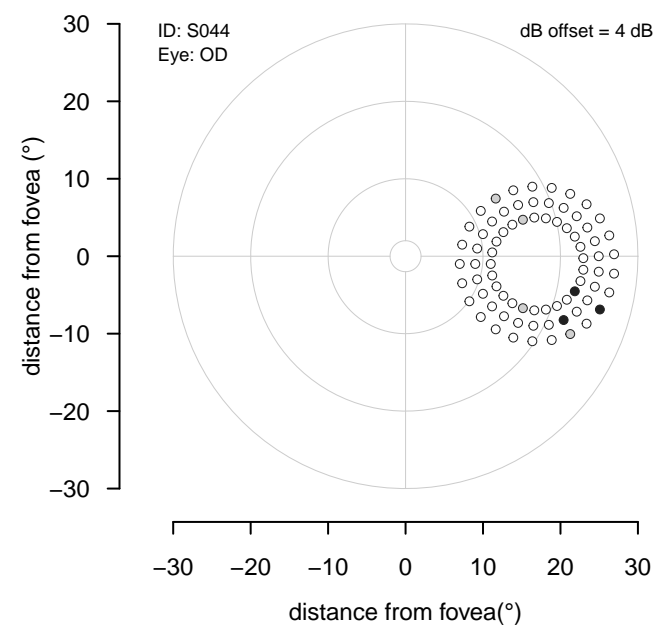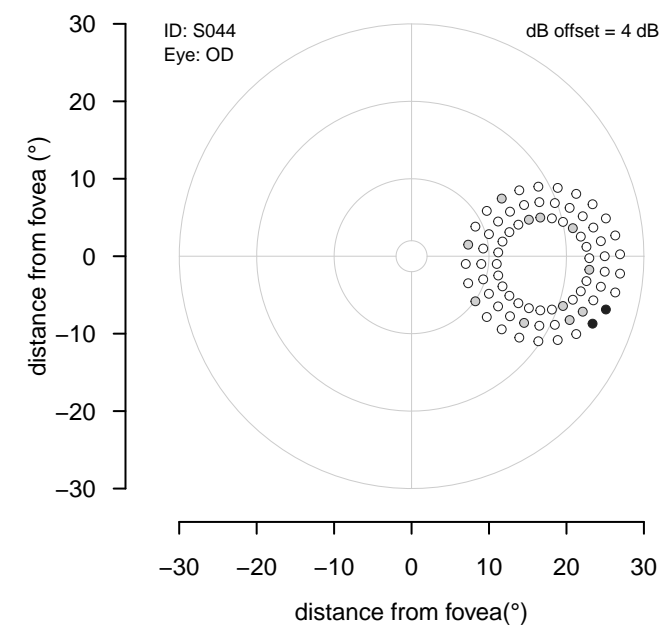

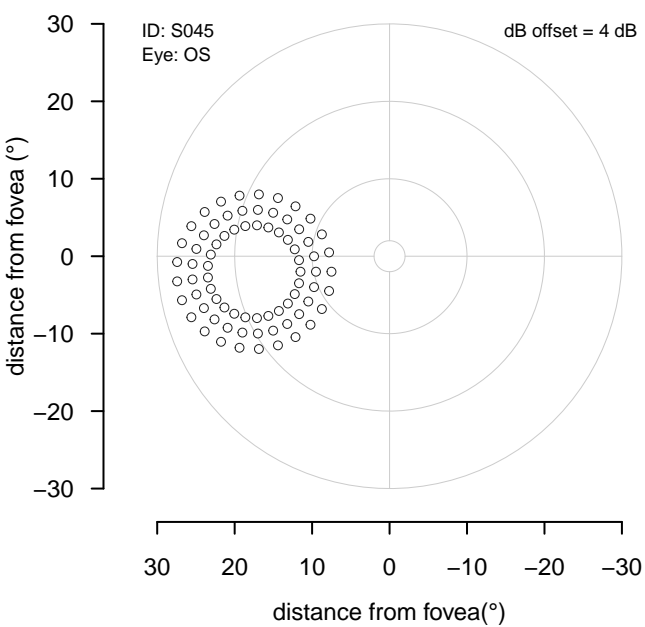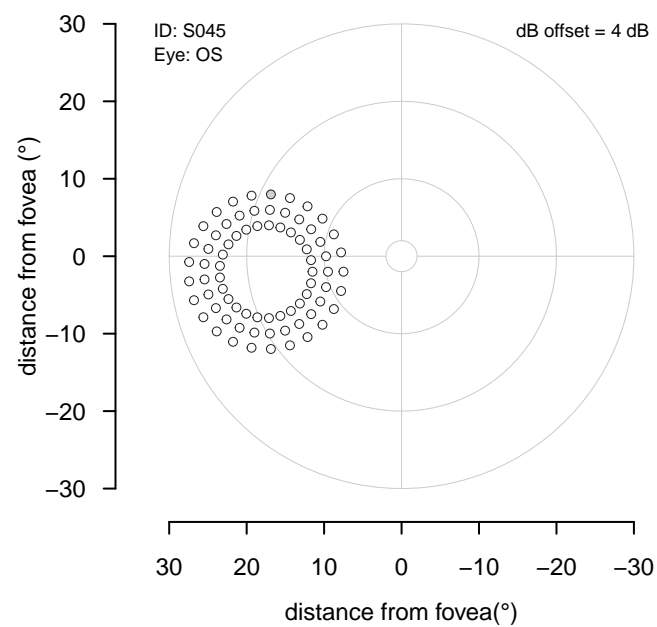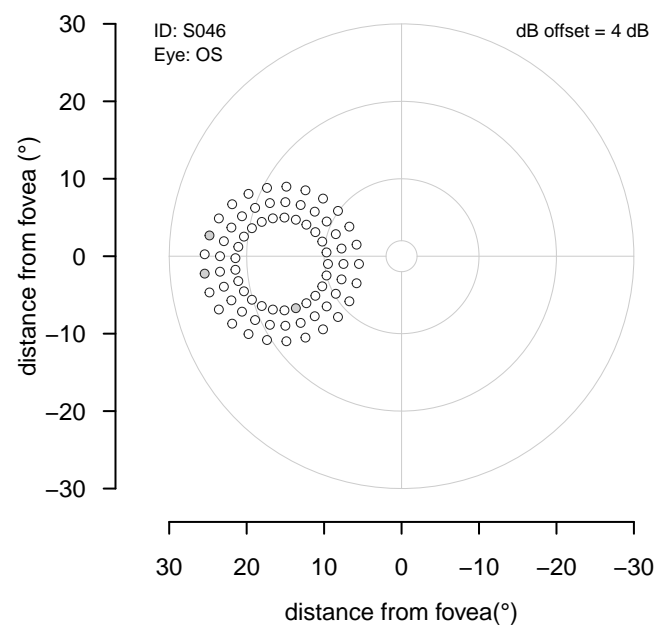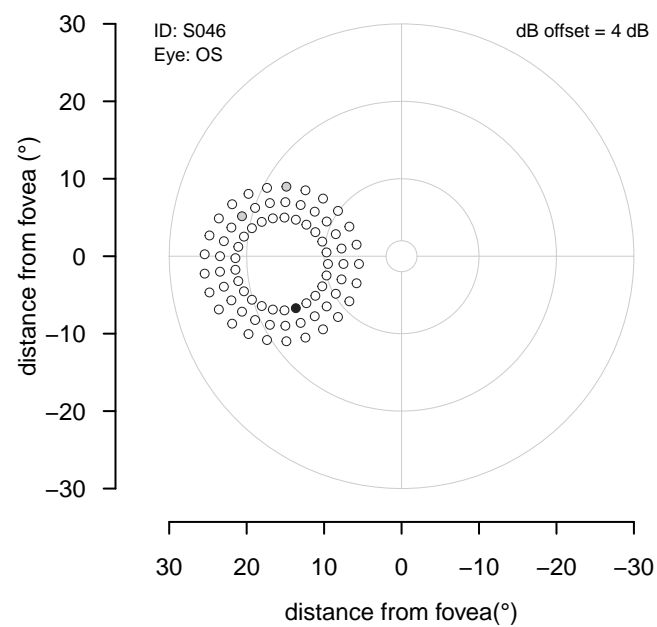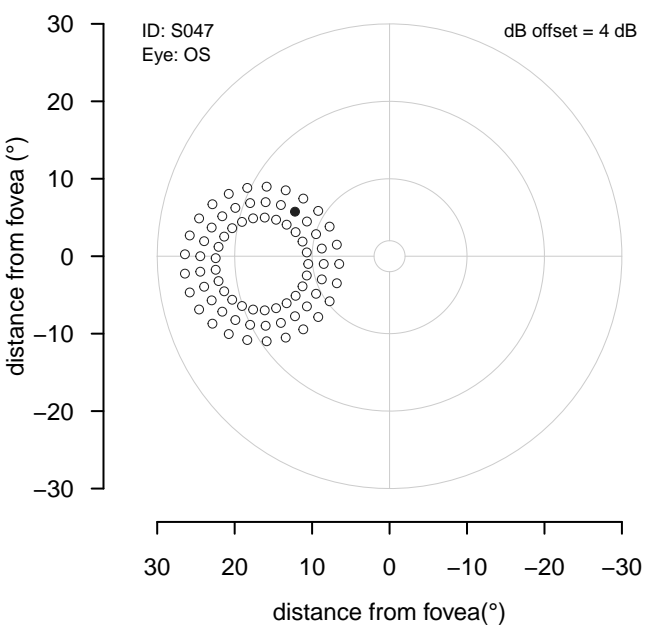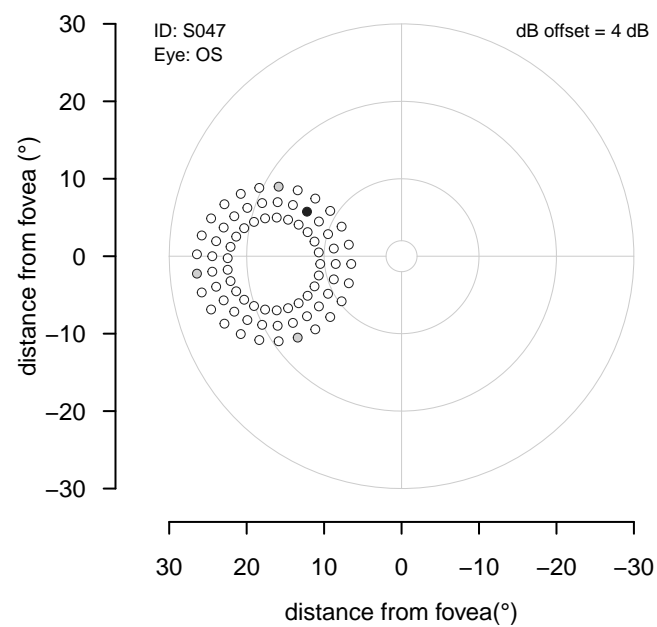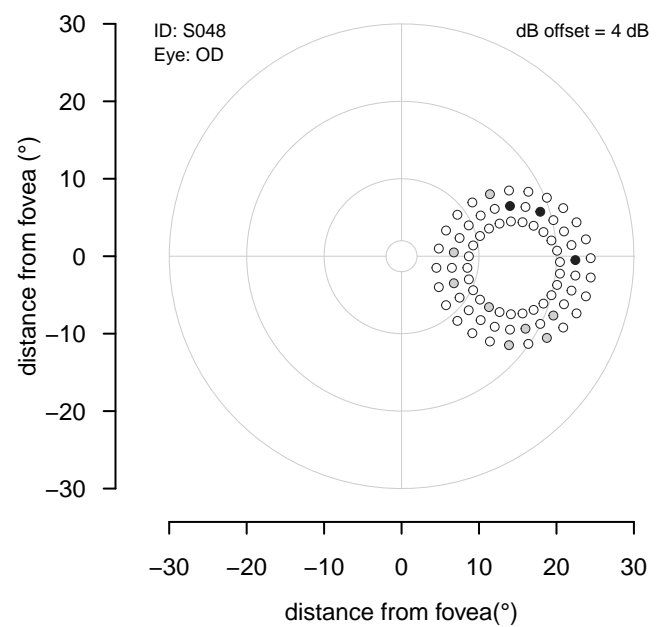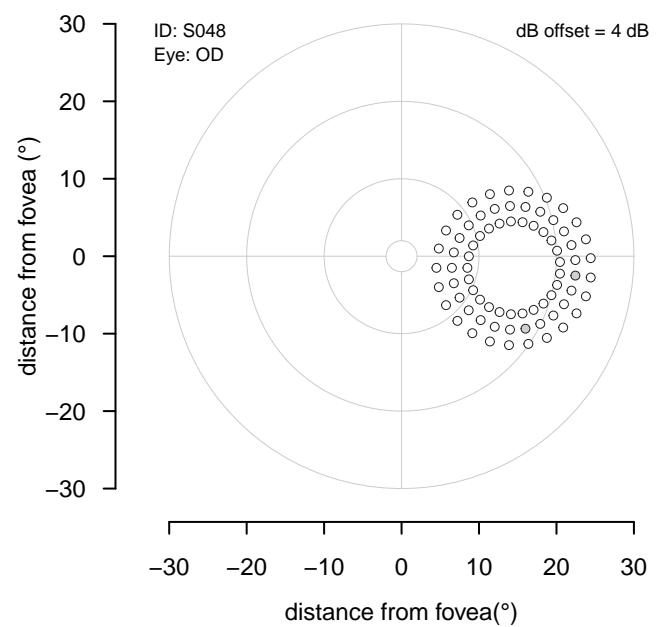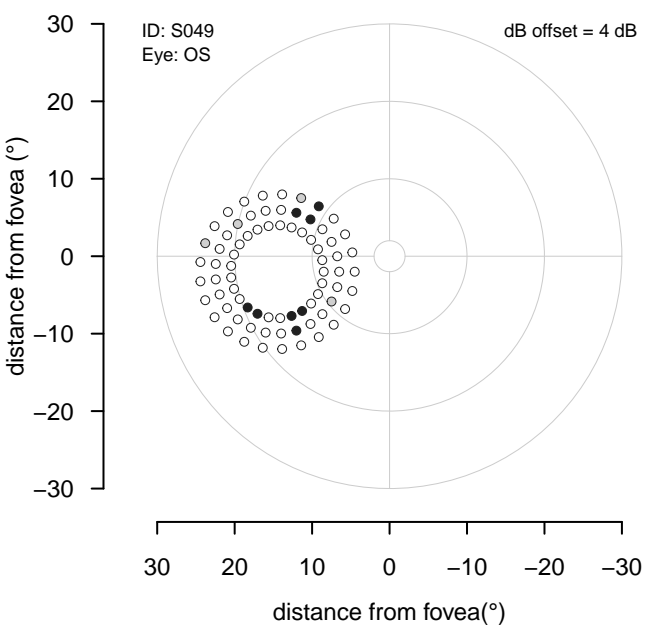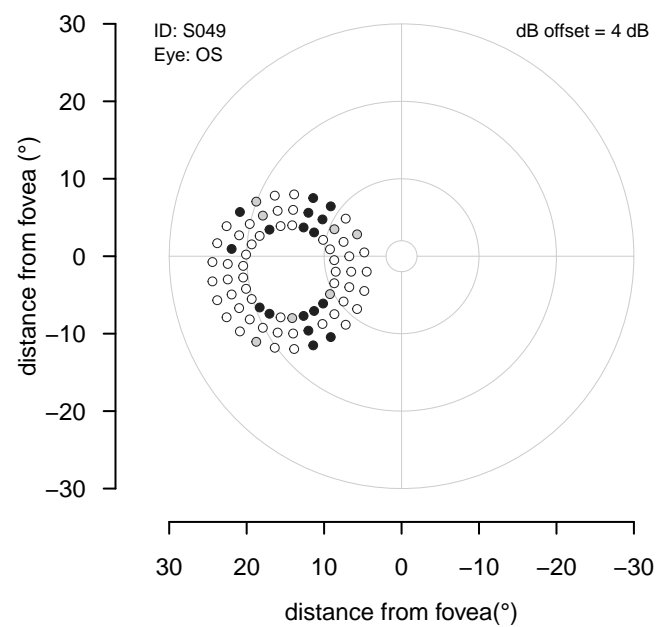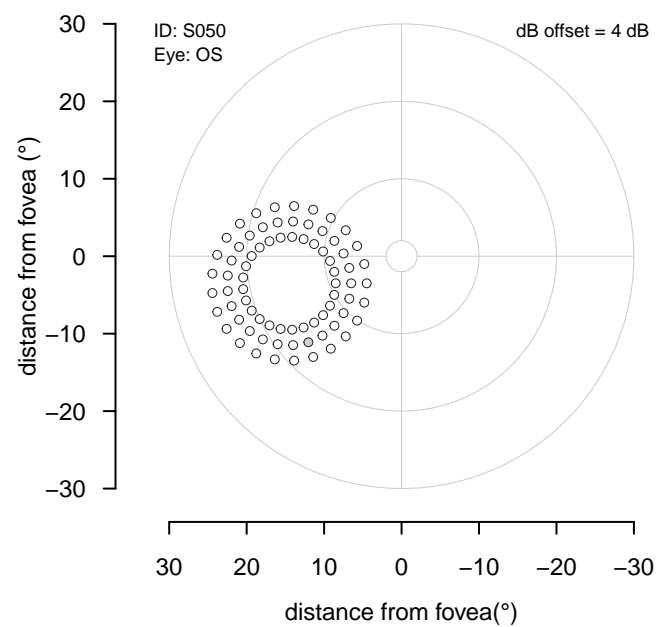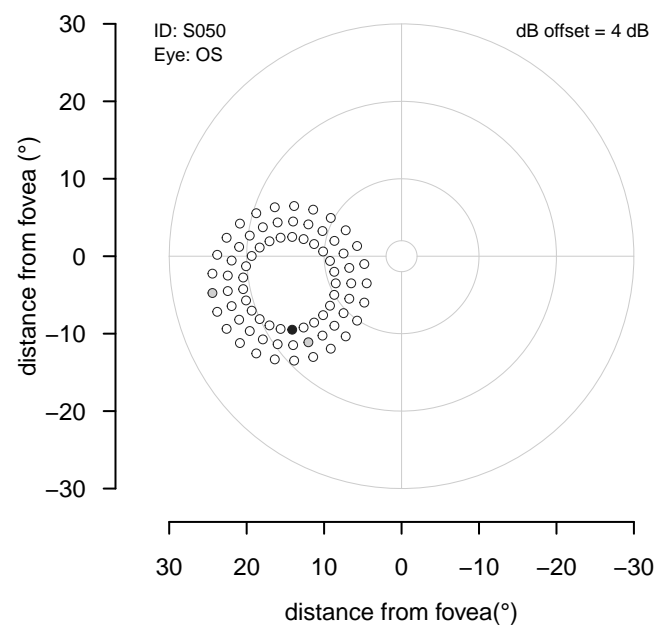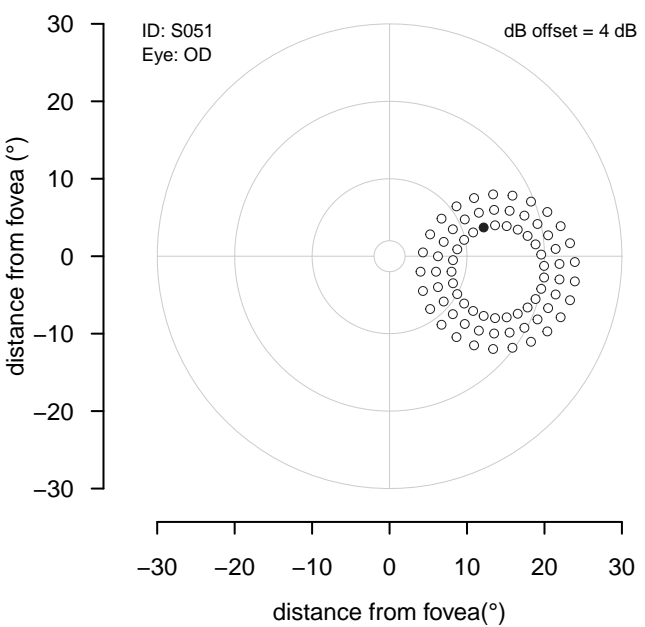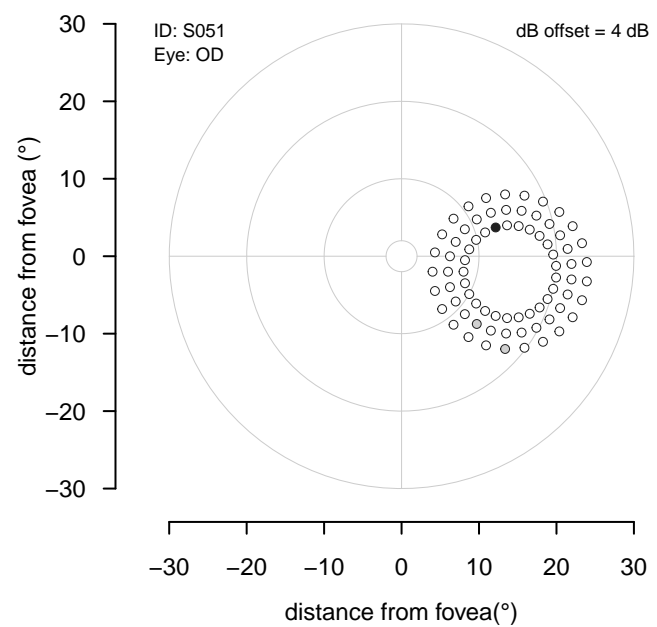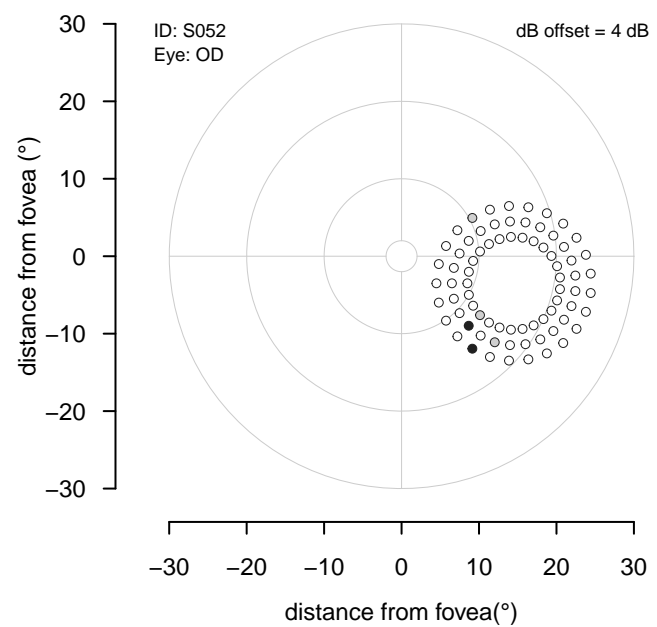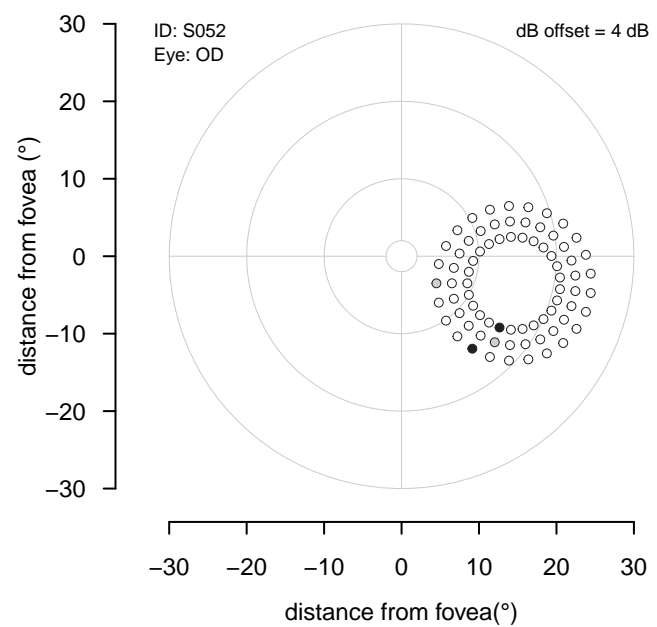

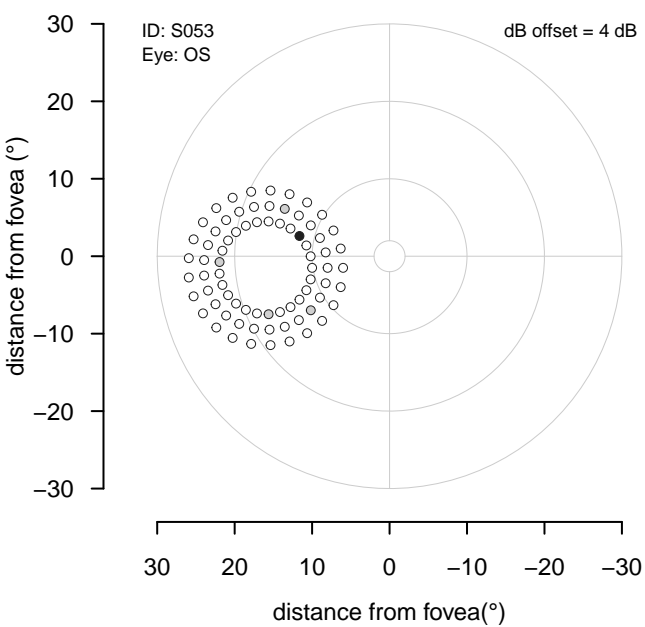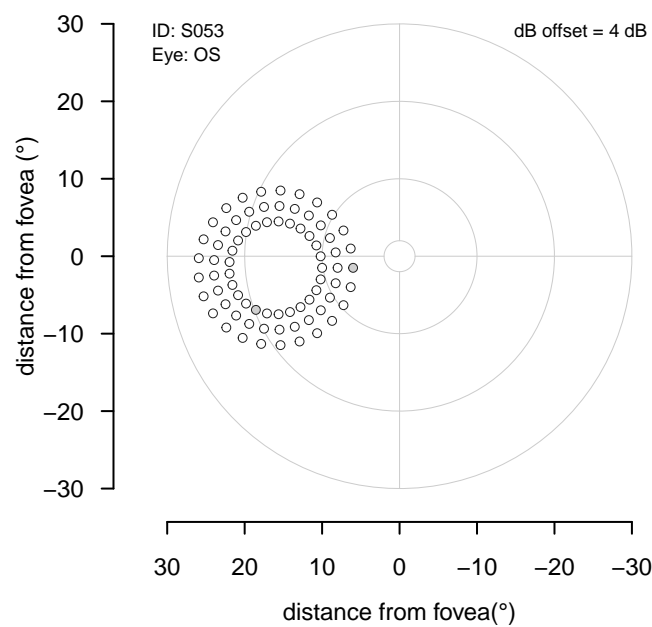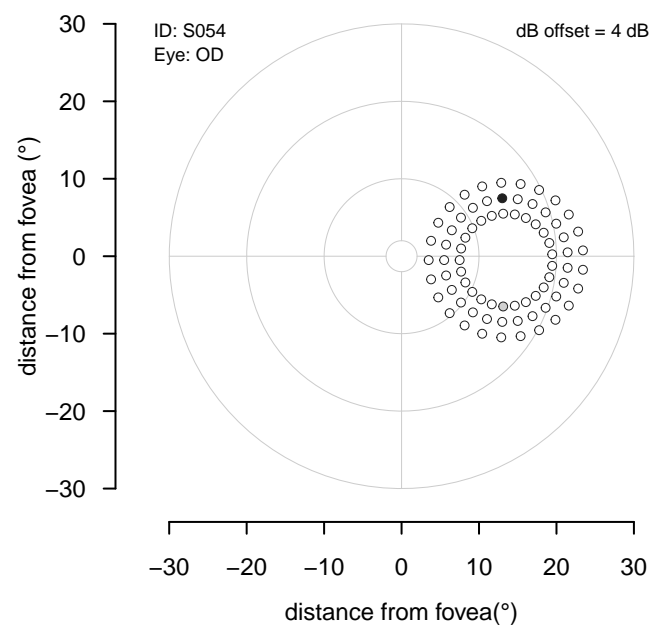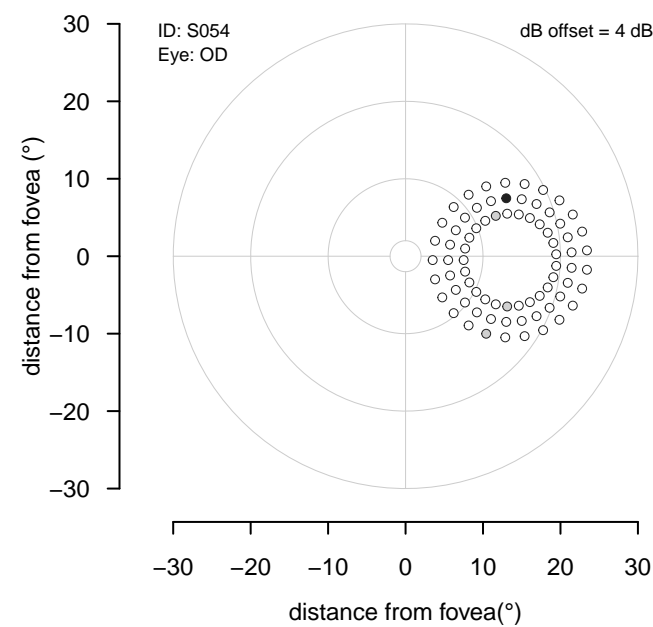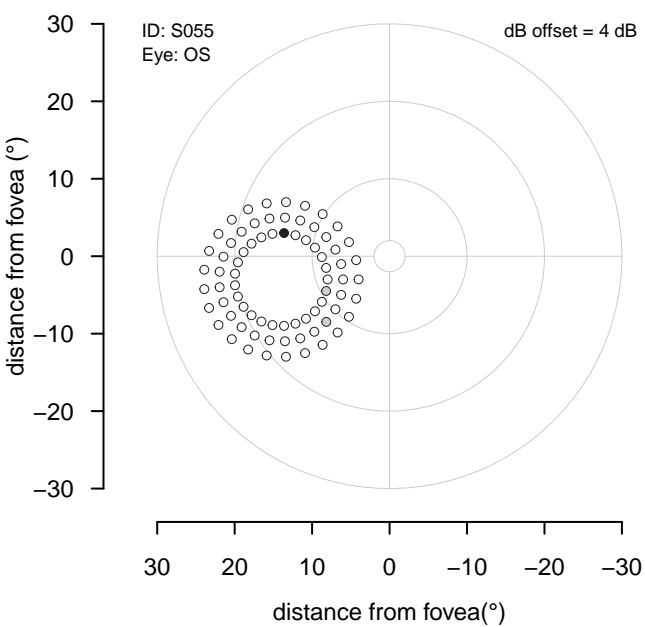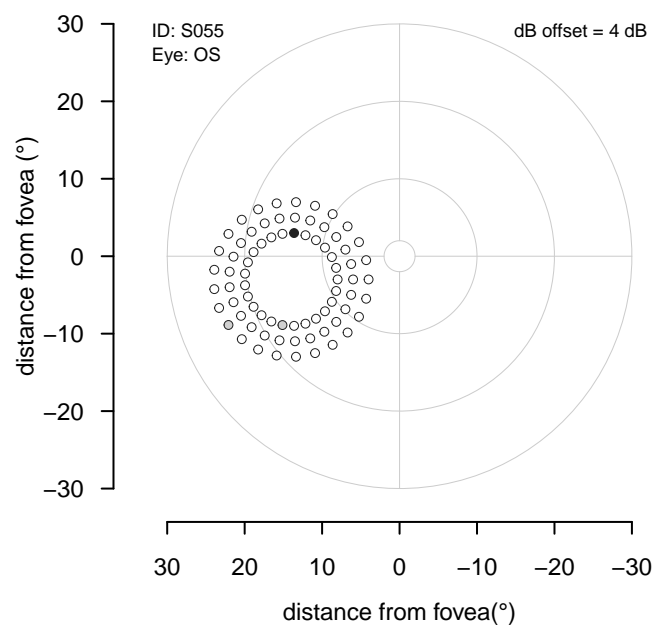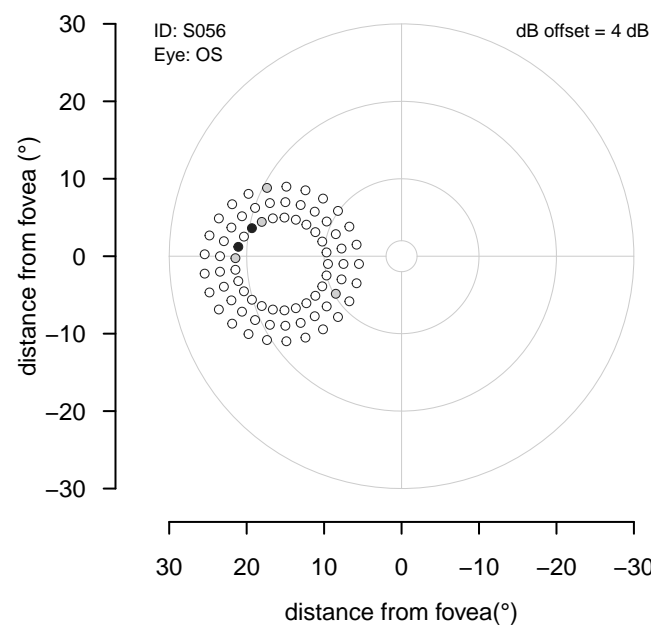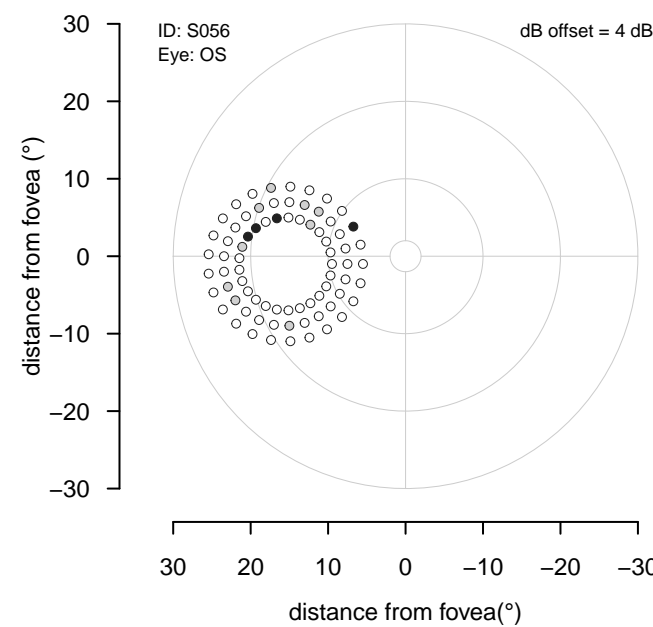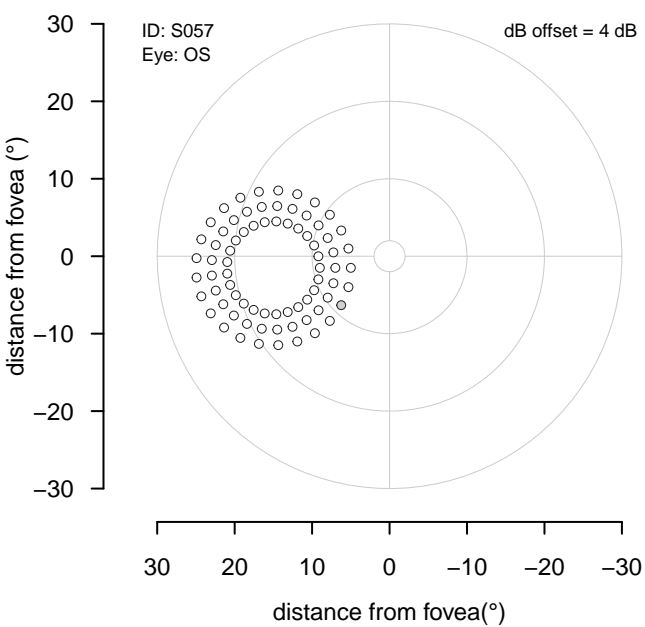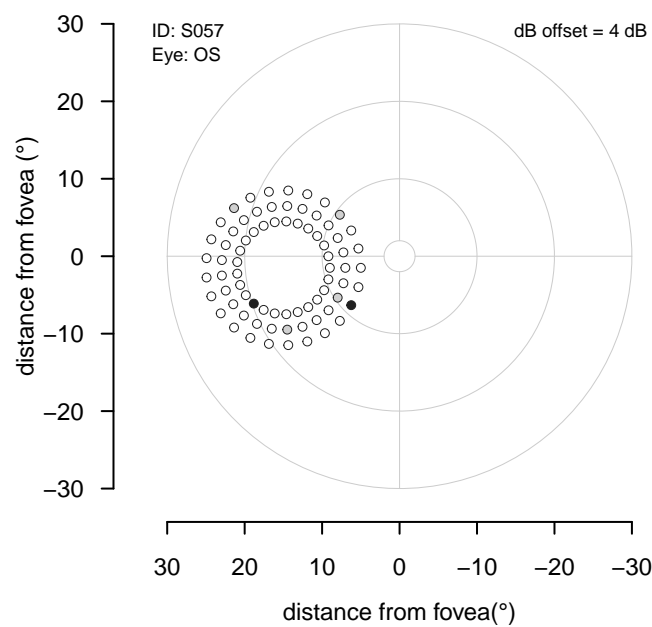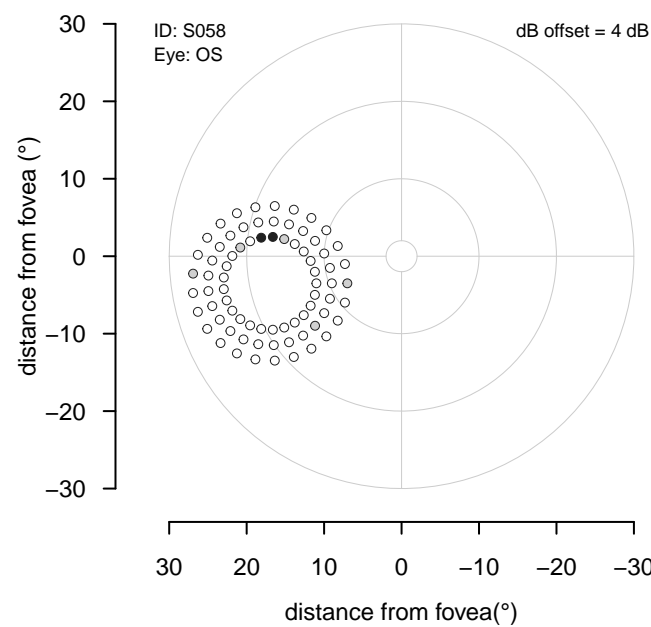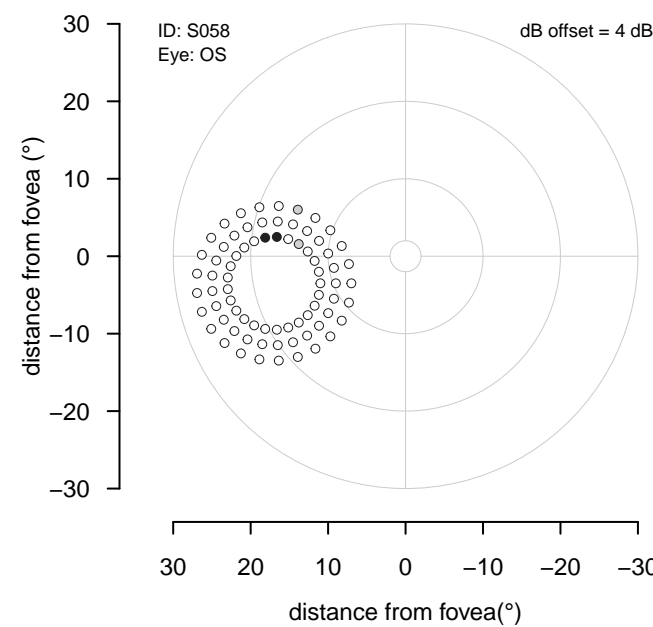

Supplement: Supplementary file 1 — Appendix S1. (ZIP 3.30 MB) [file 44402_2025_4507042_MOESM1_ESM.zip › opo70014-sup-0004-Supinfo4.pdf]

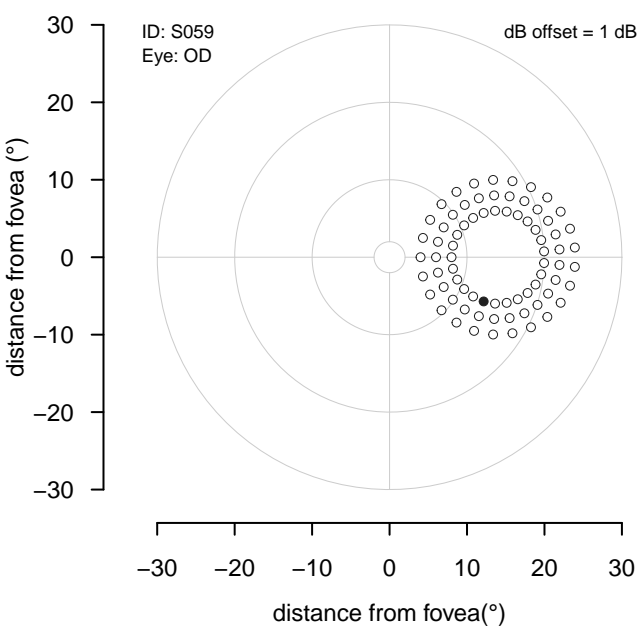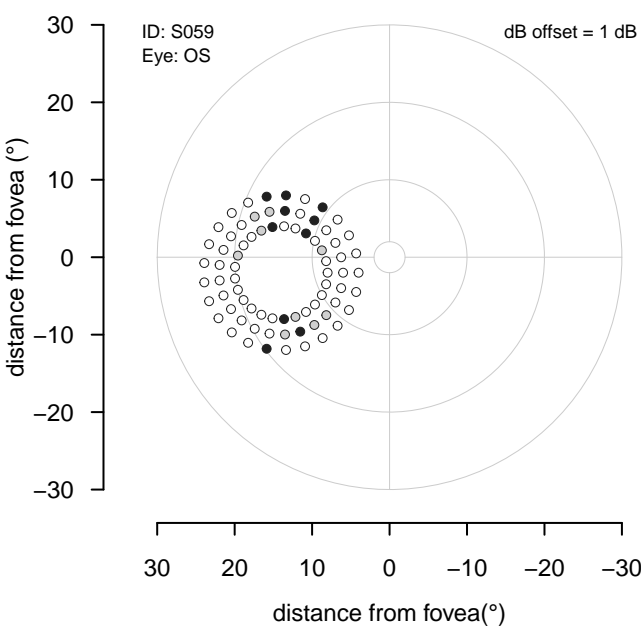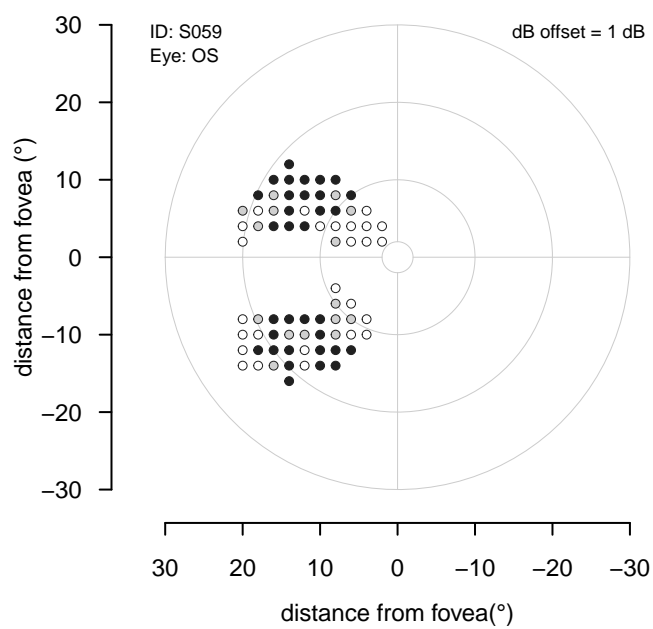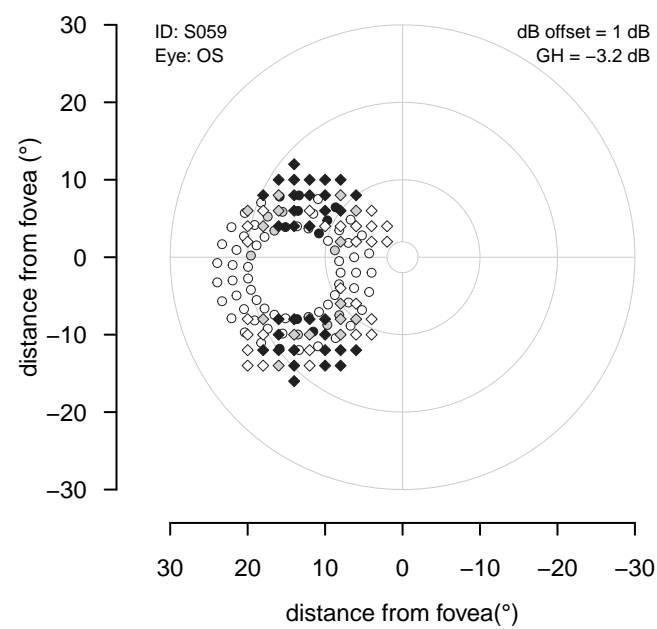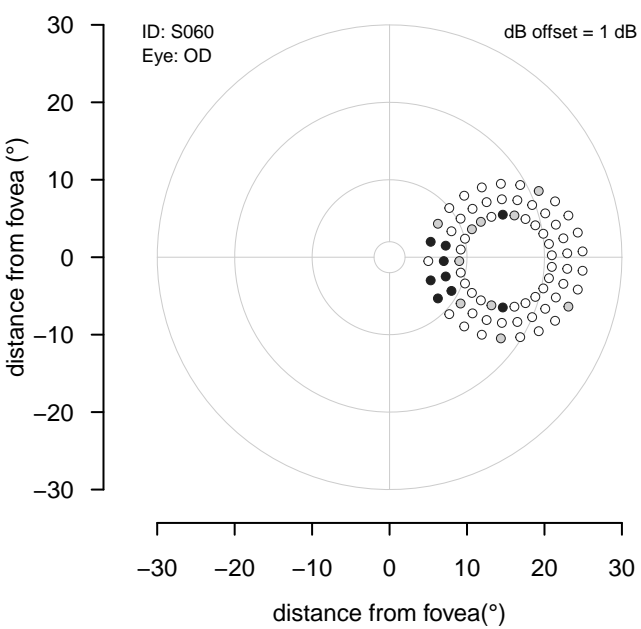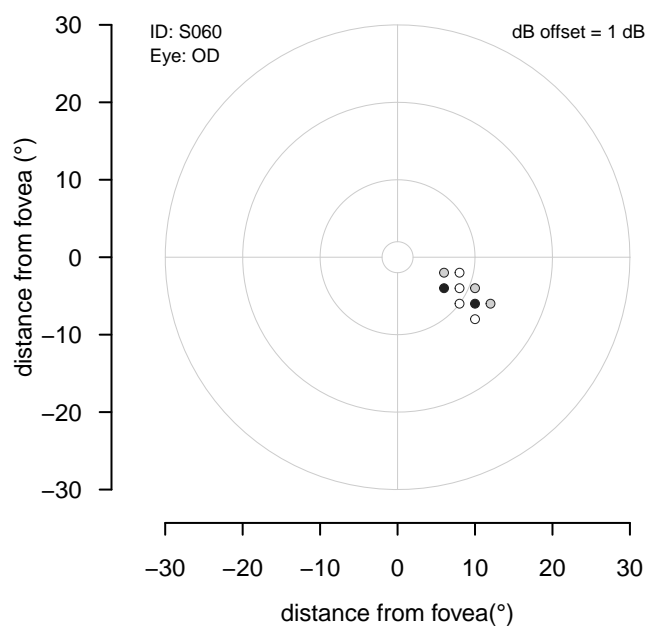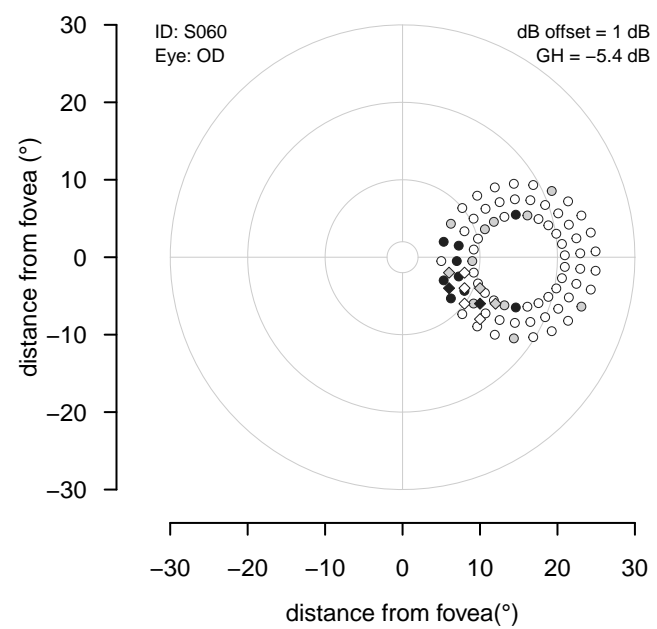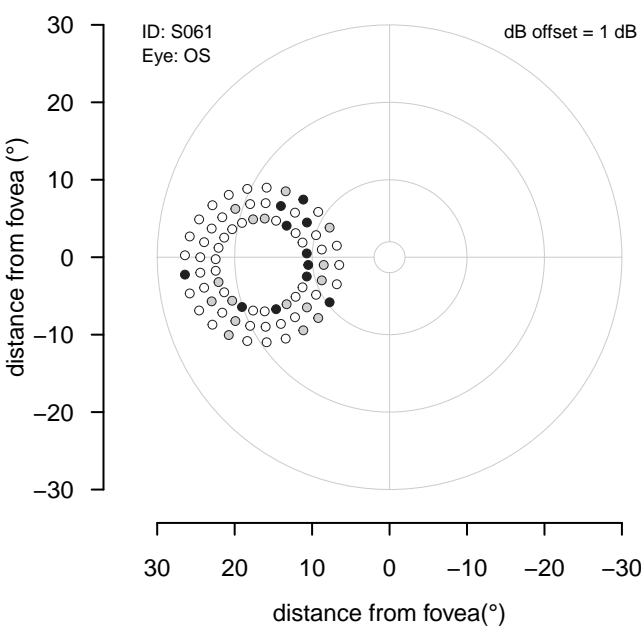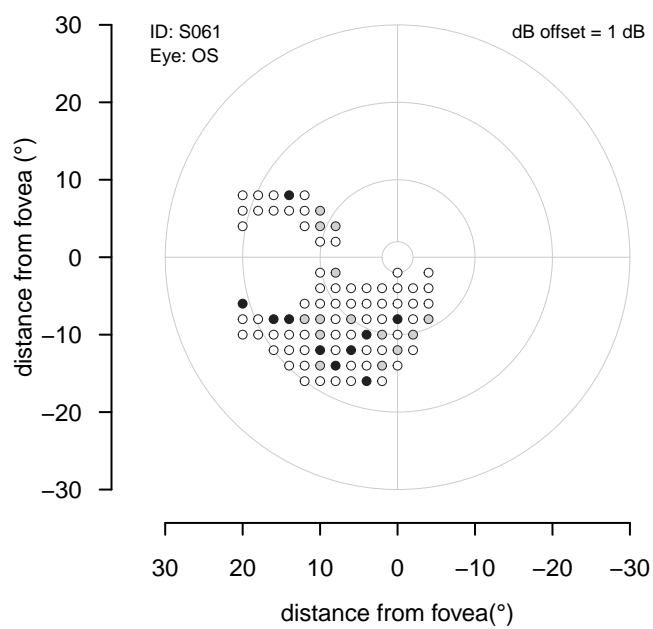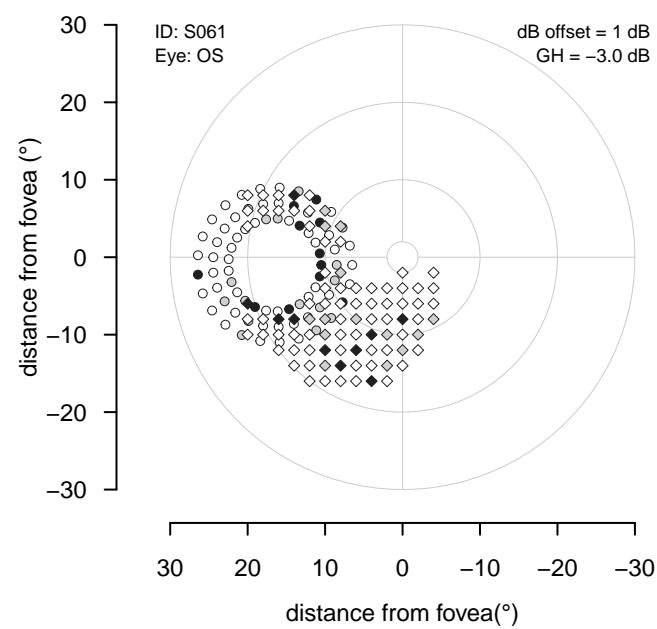

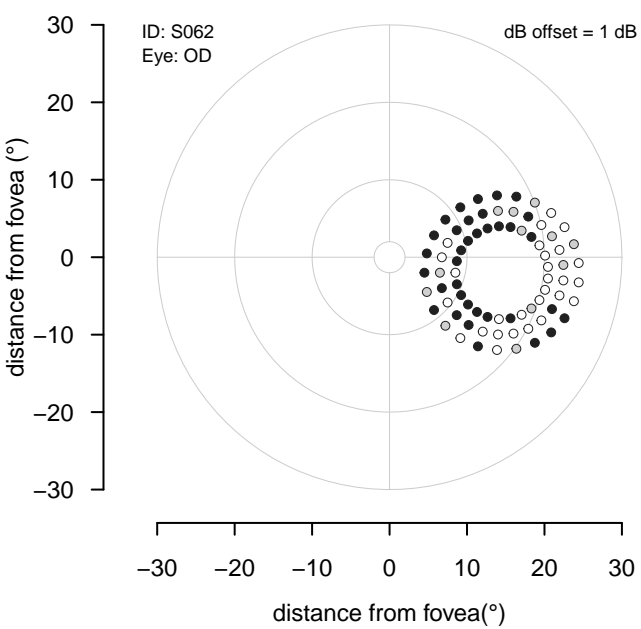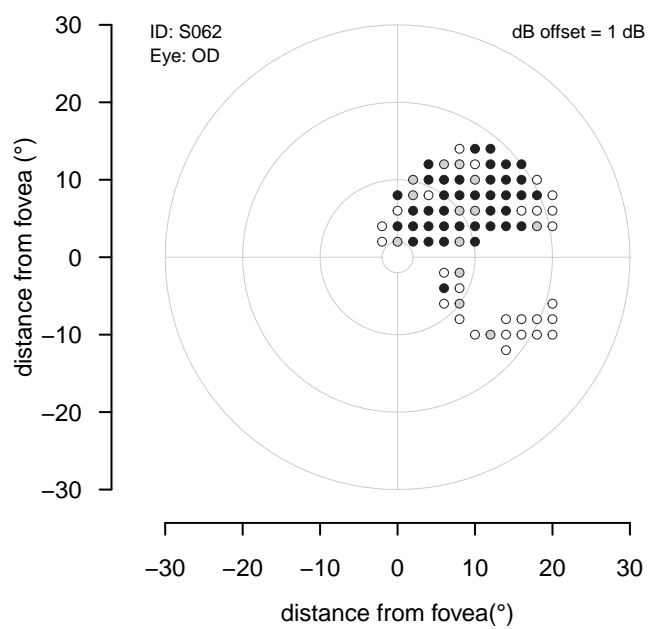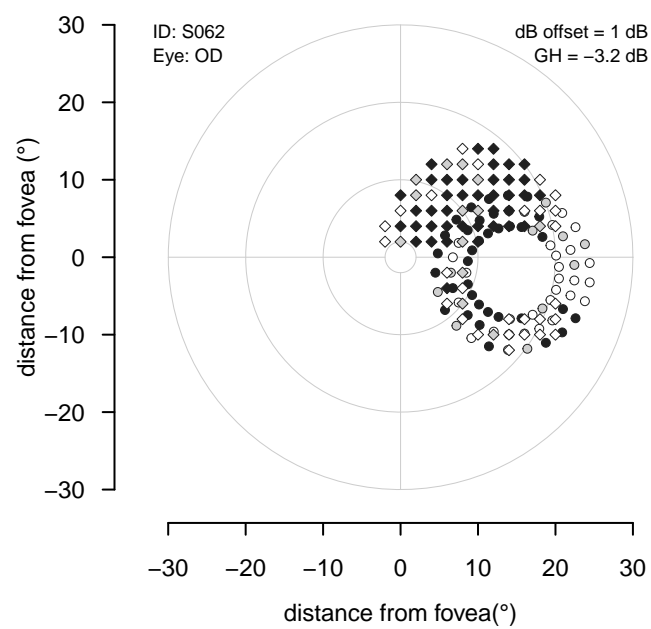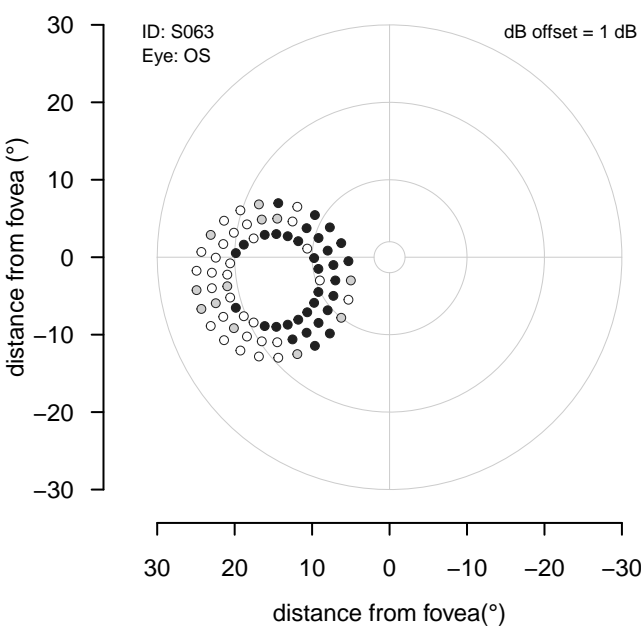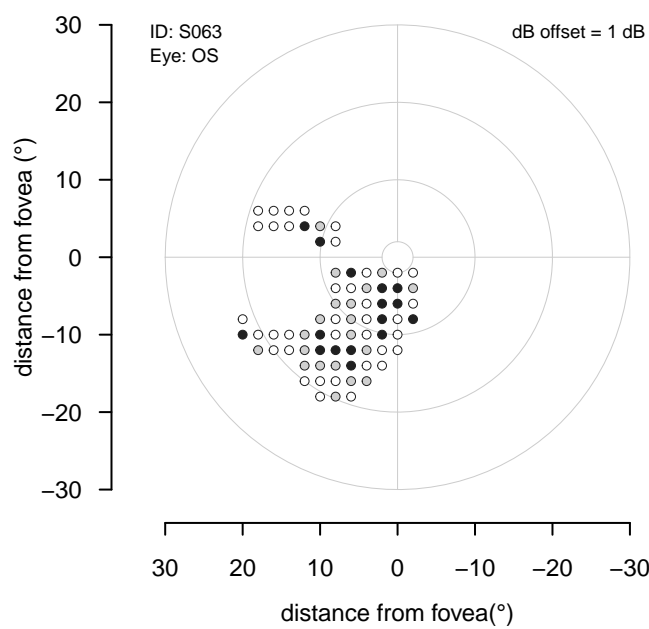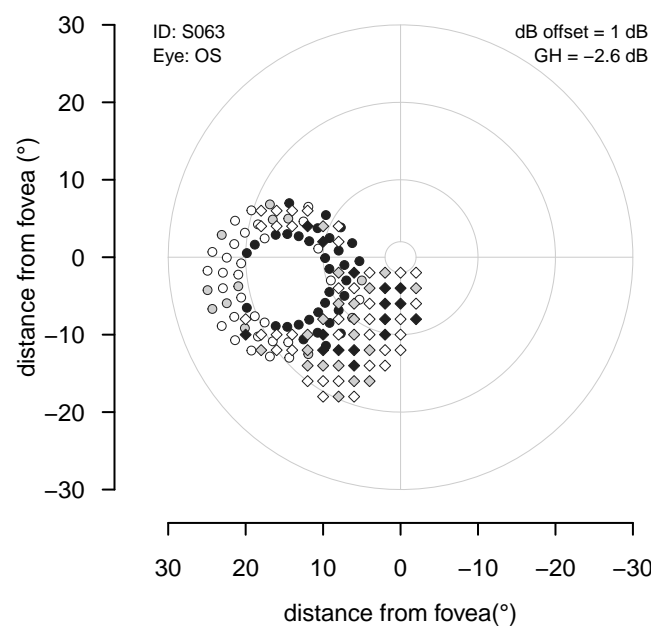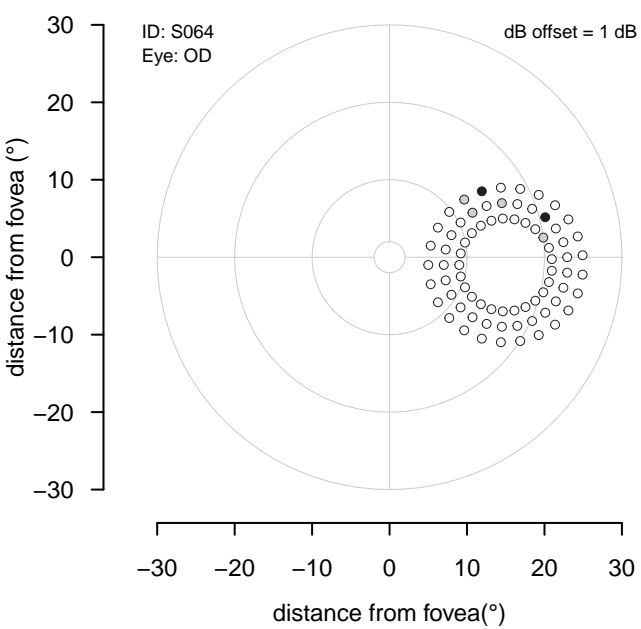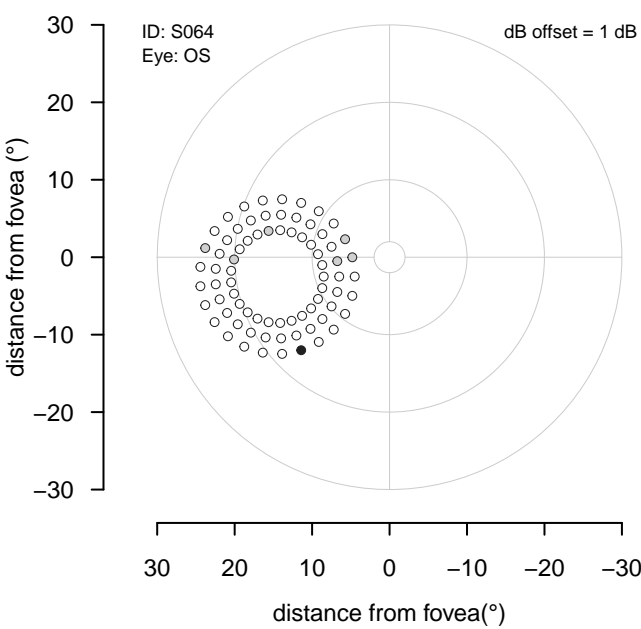

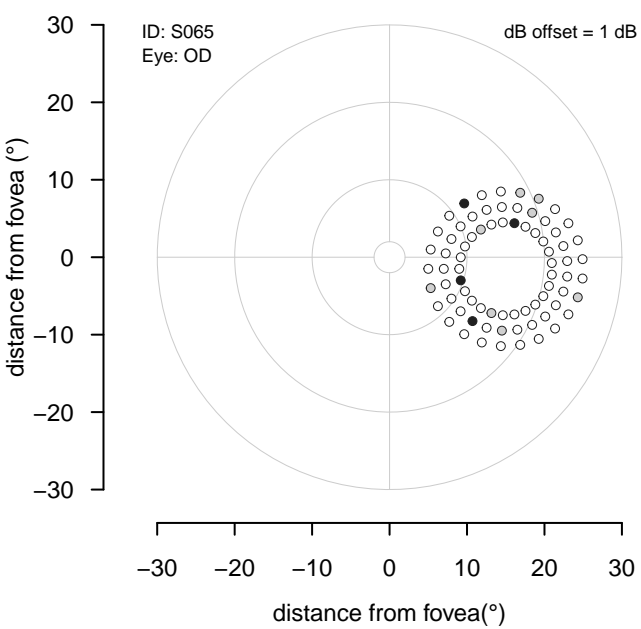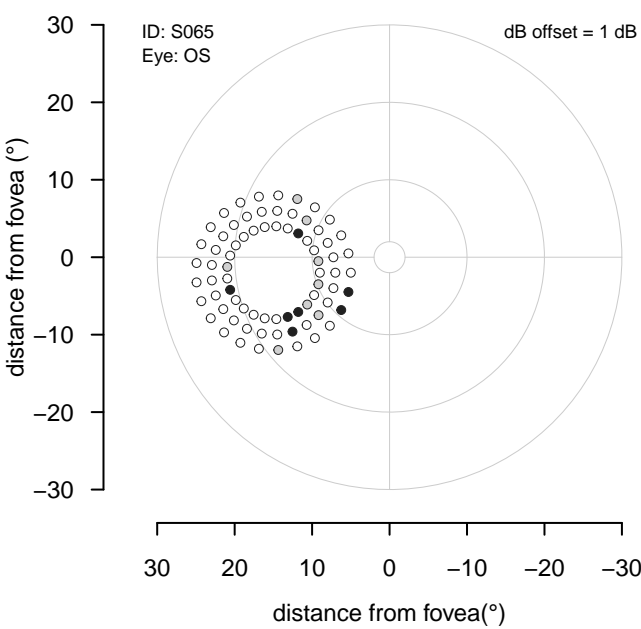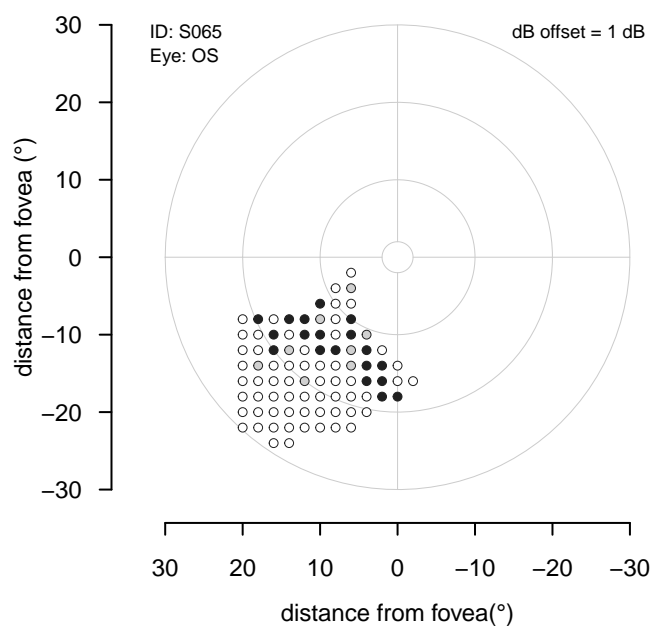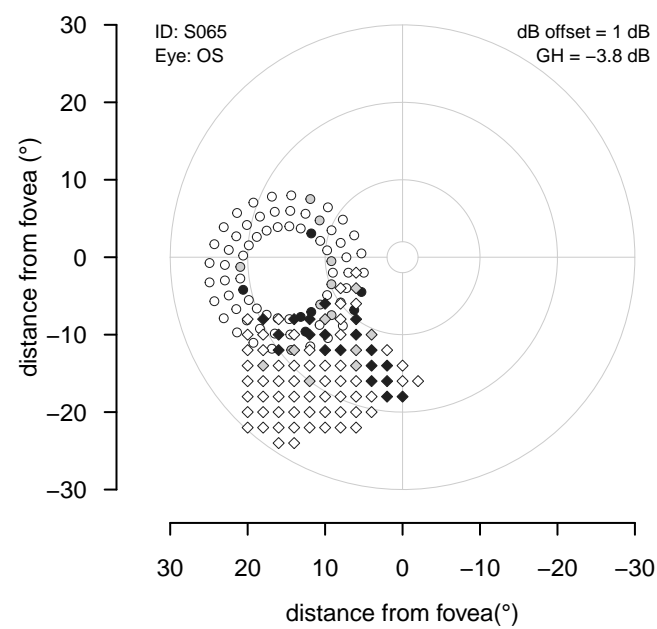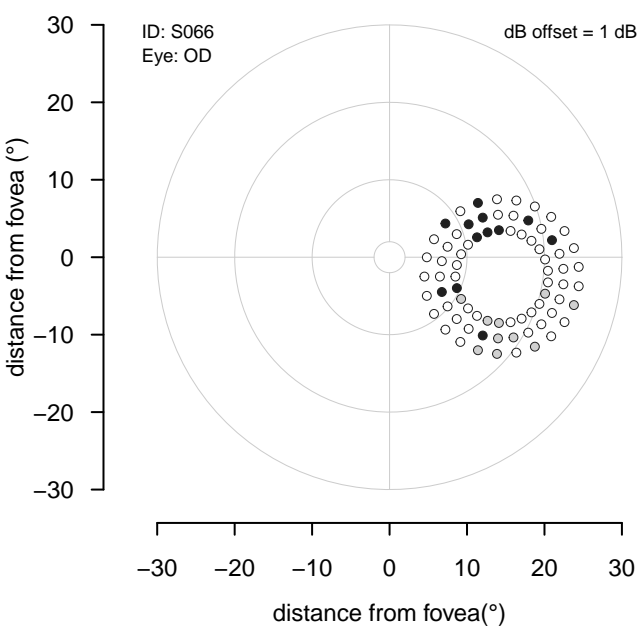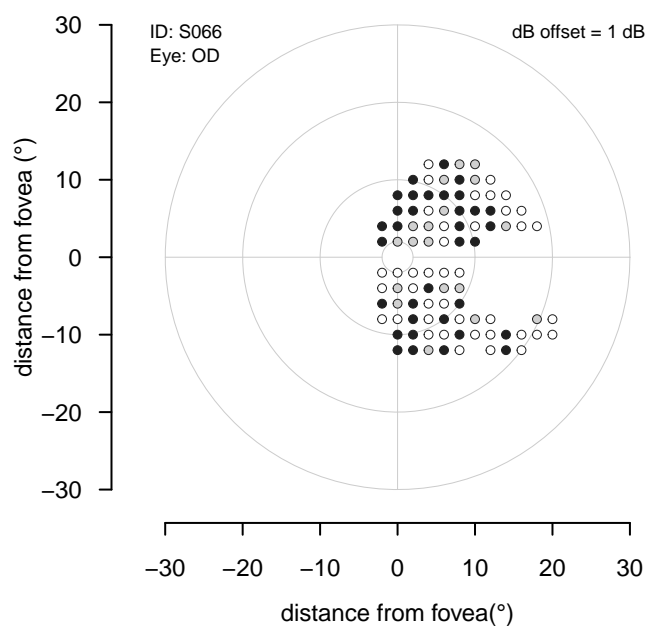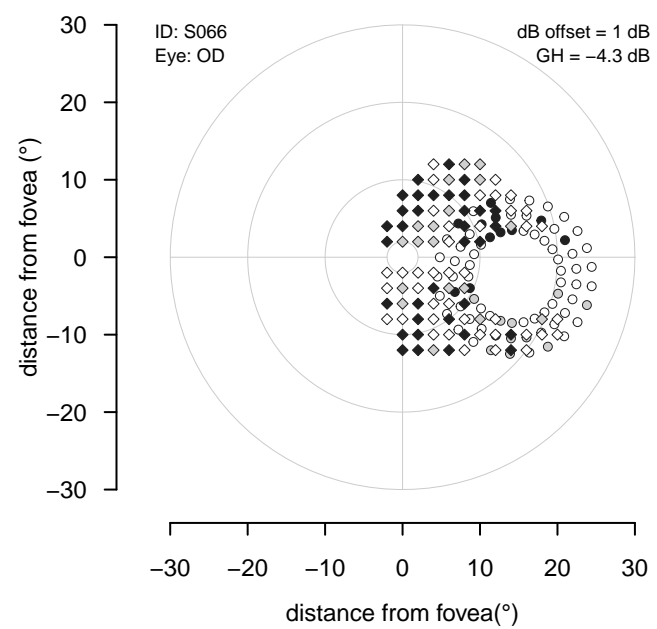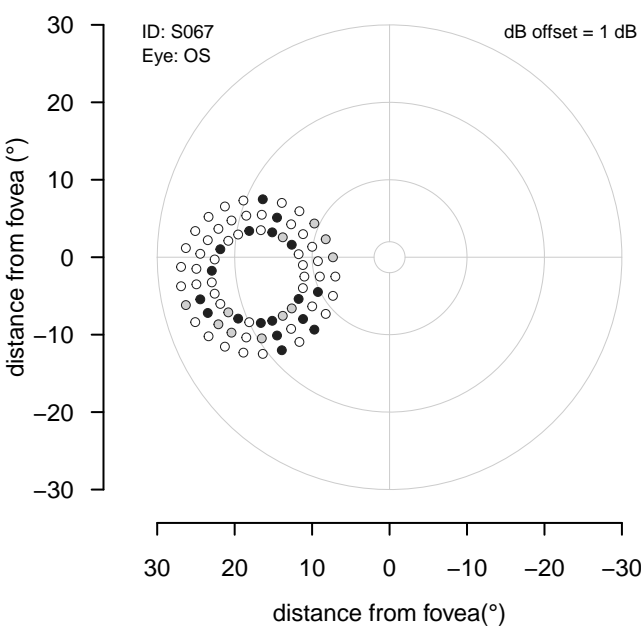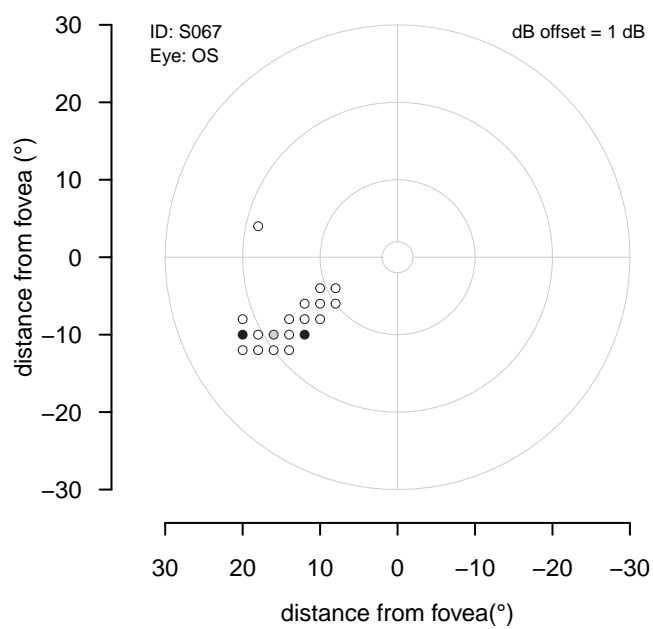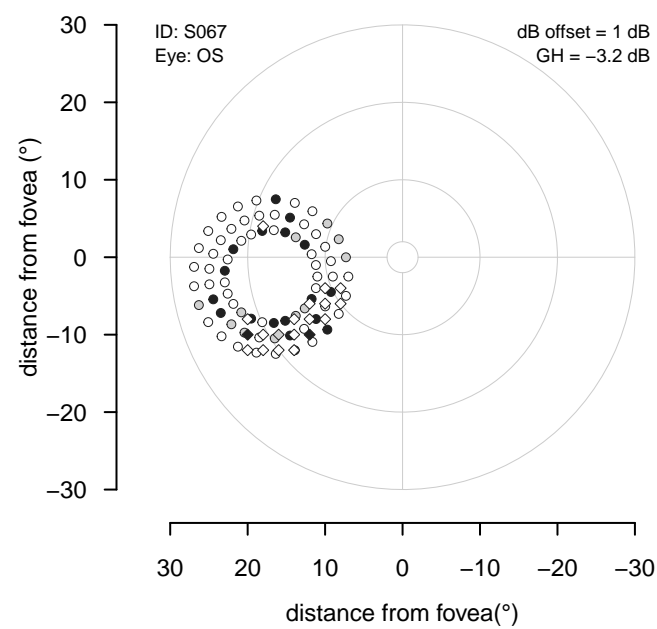

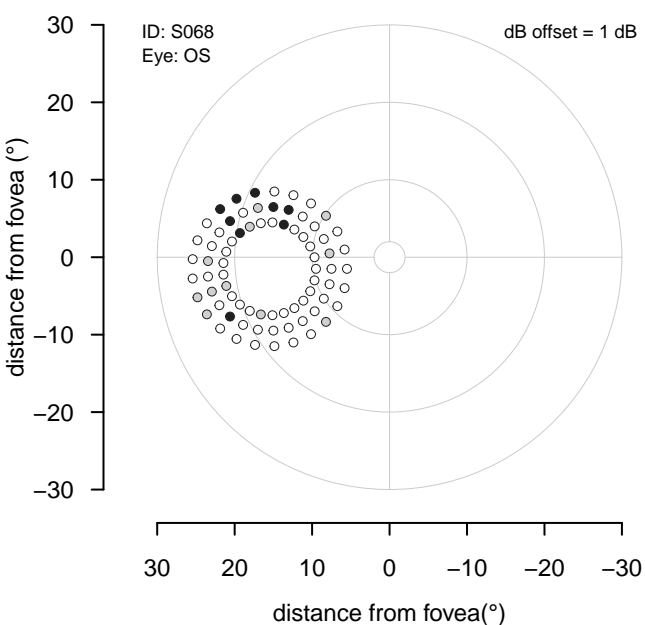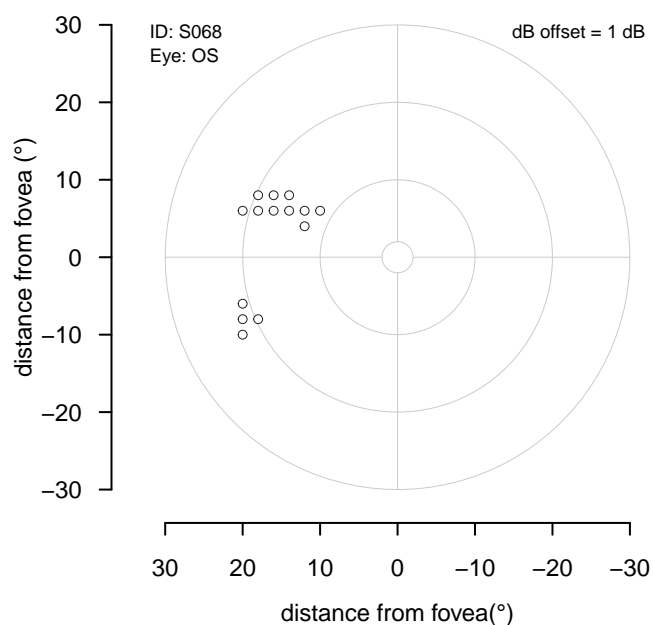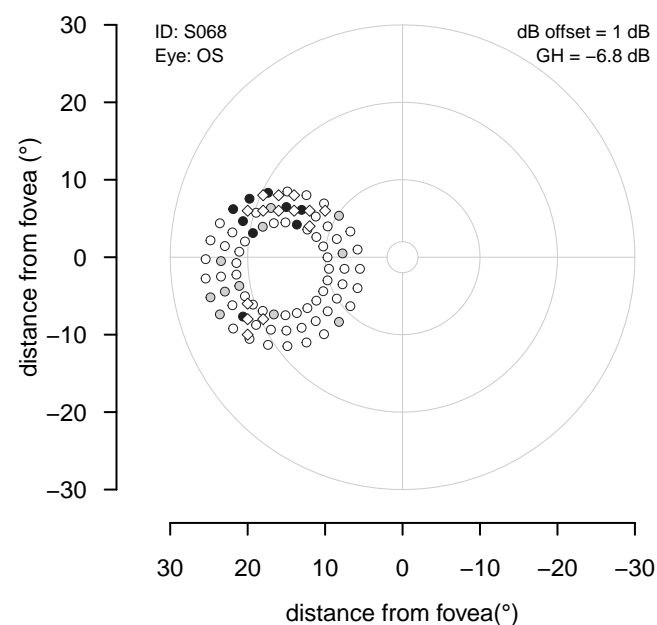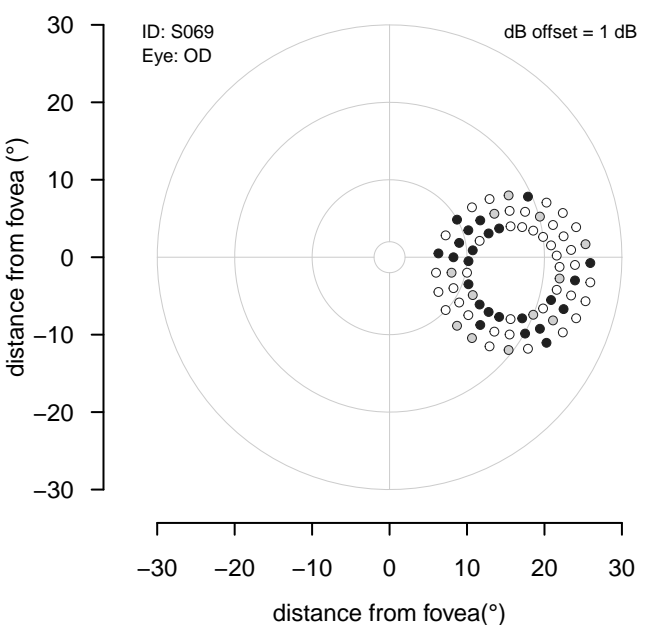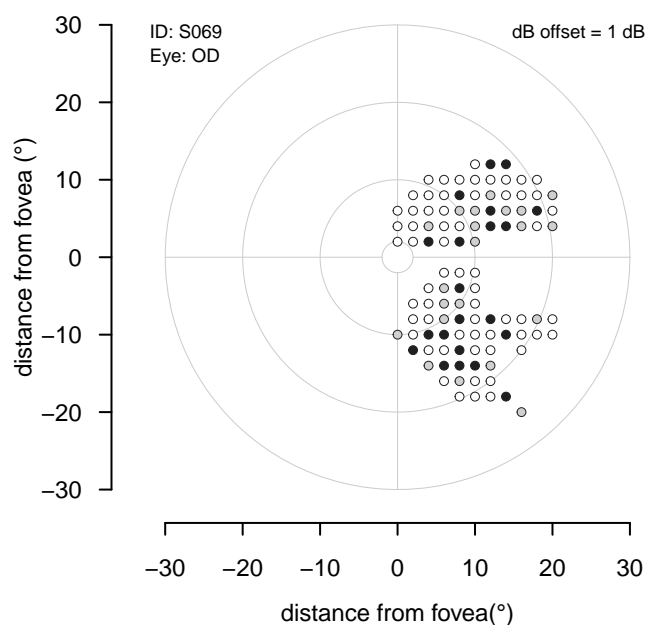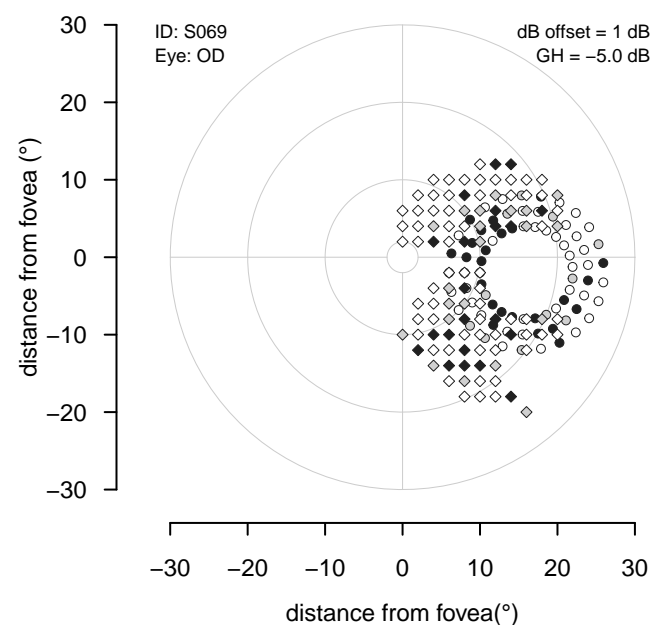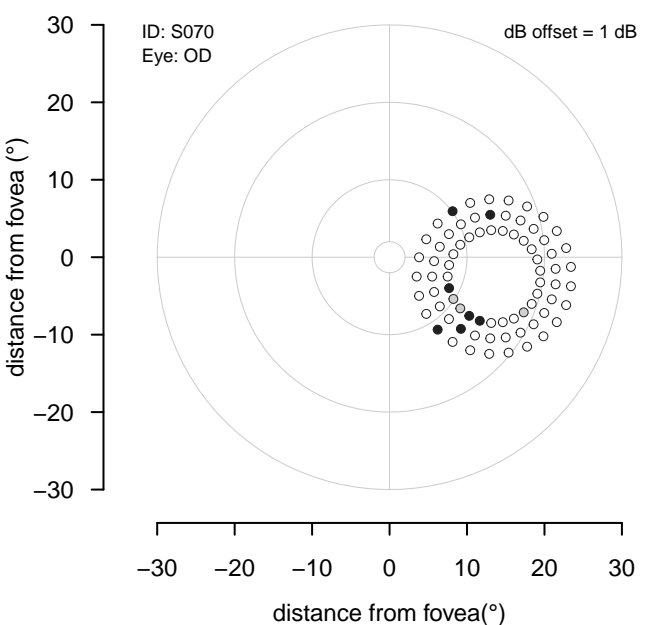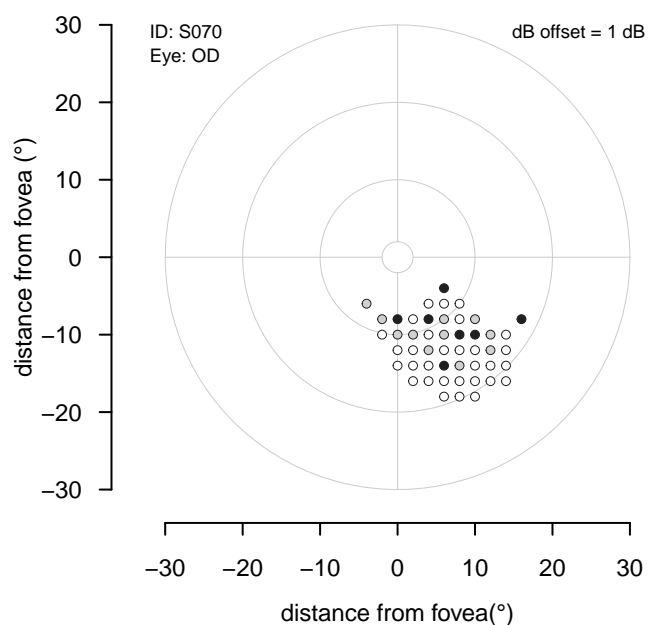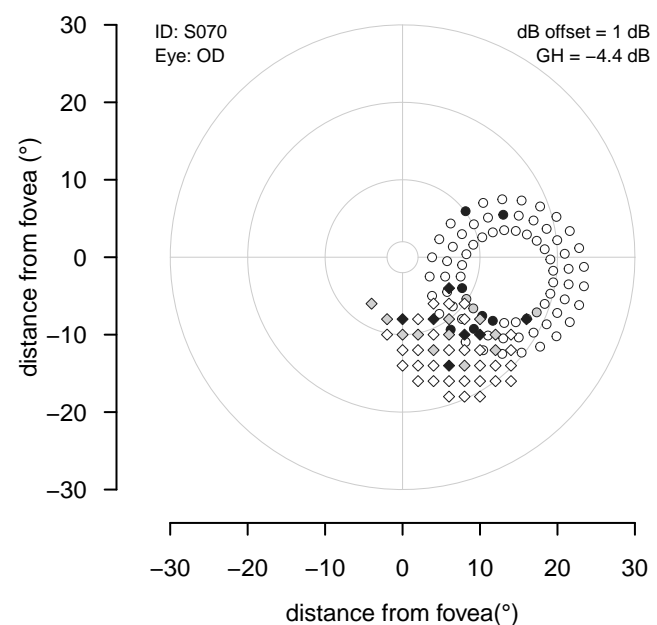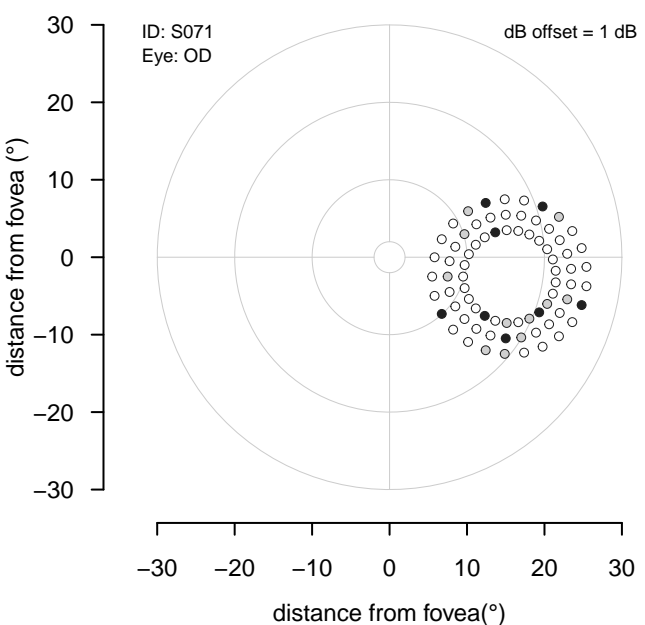

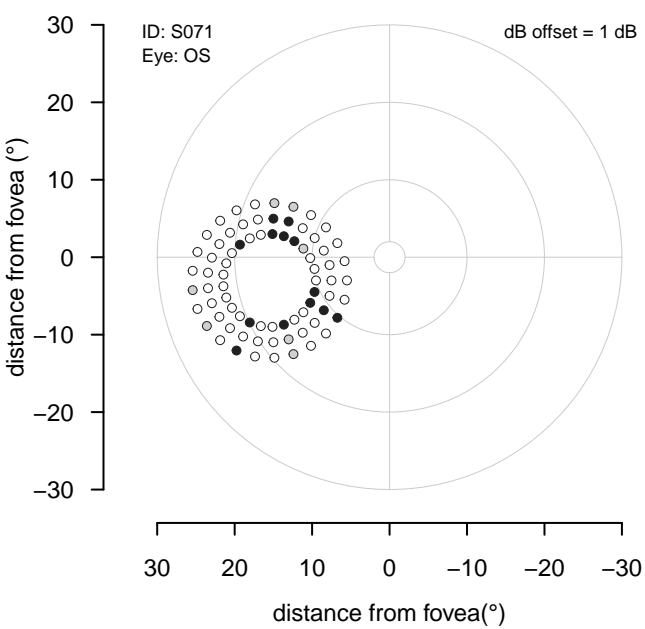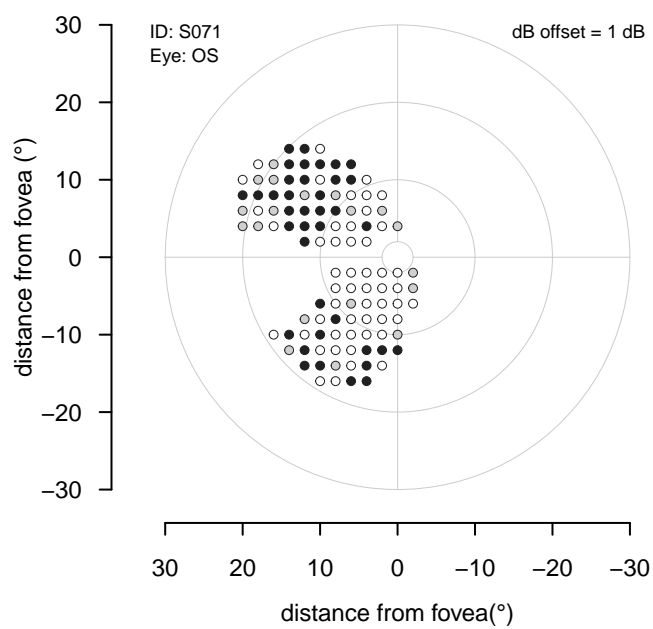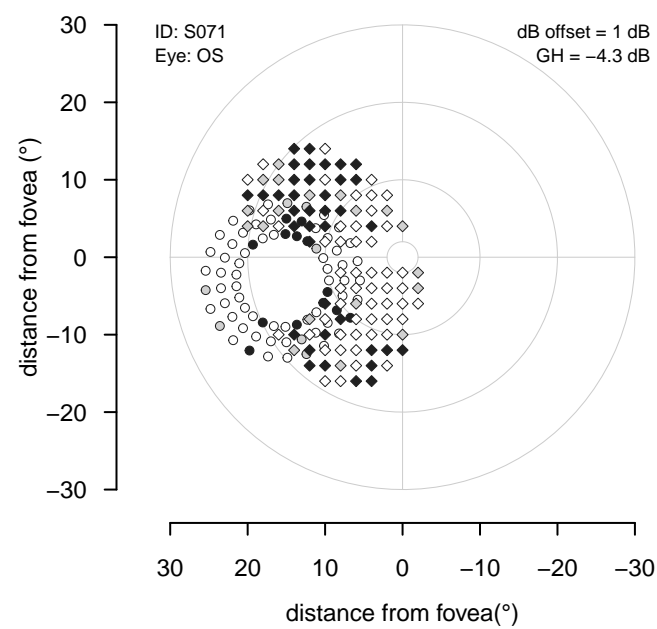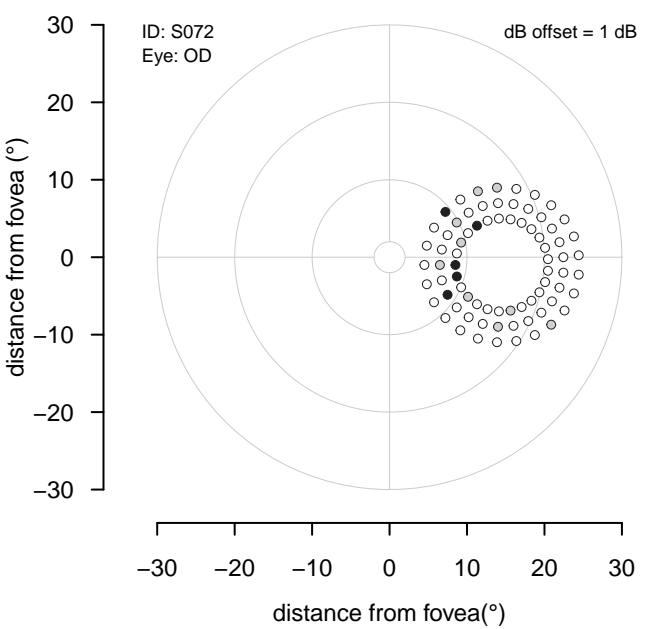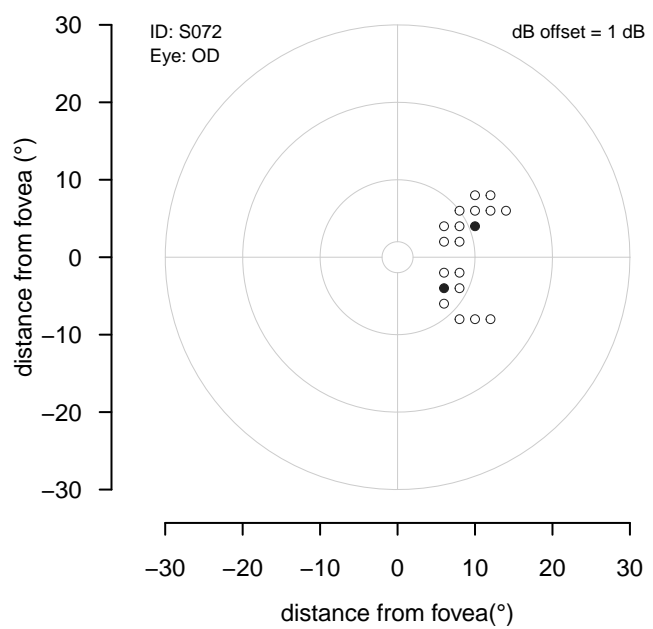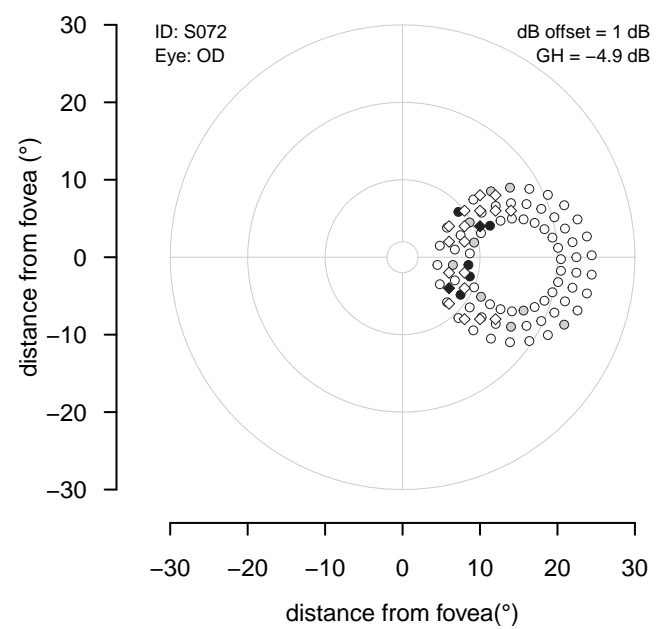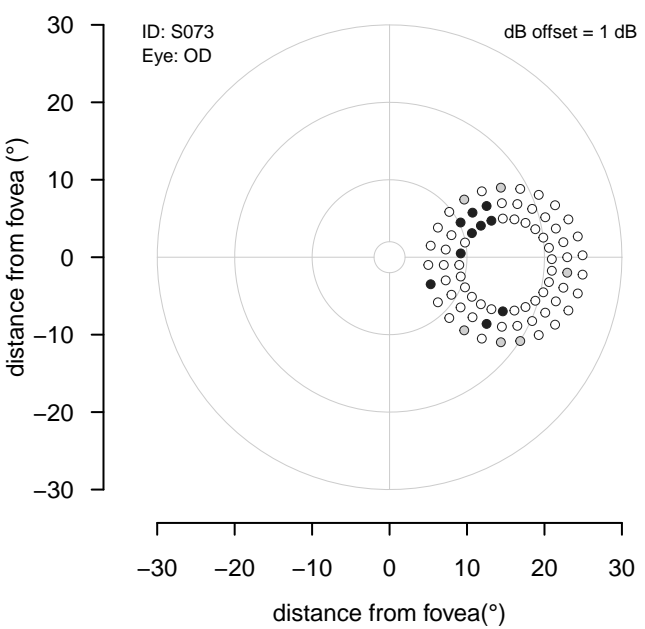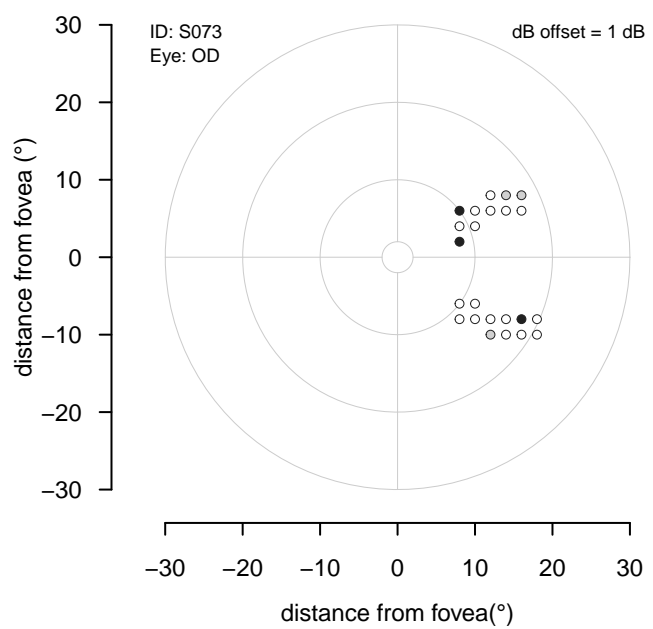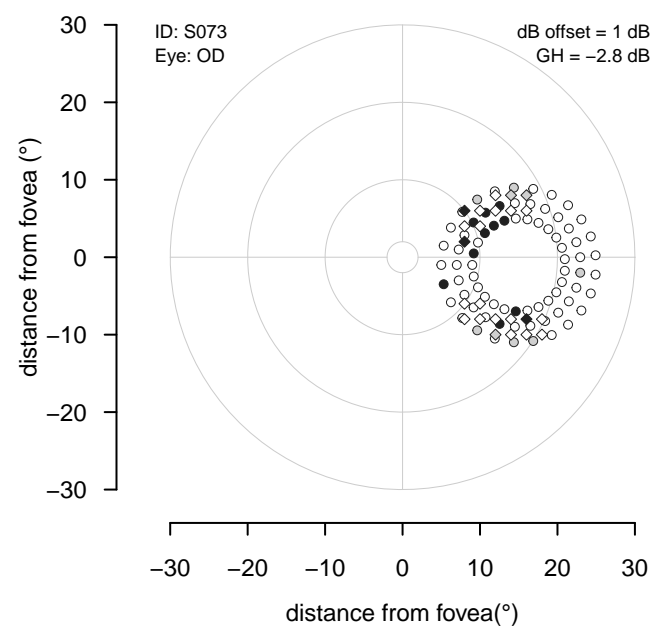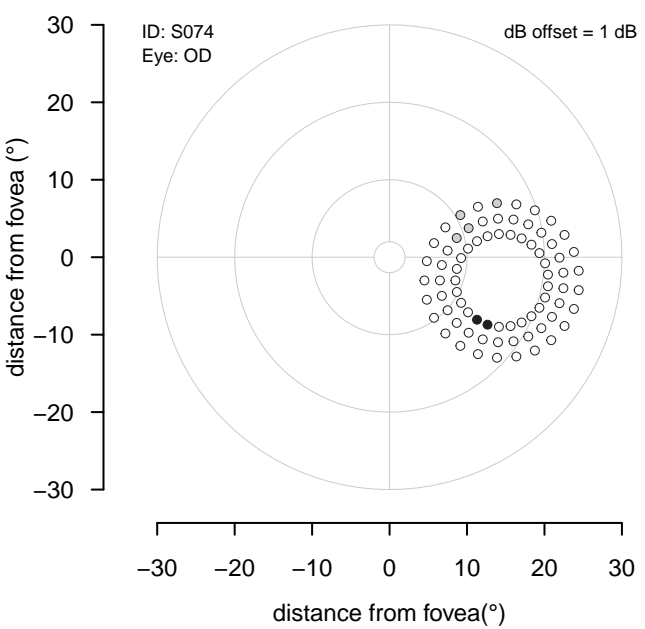

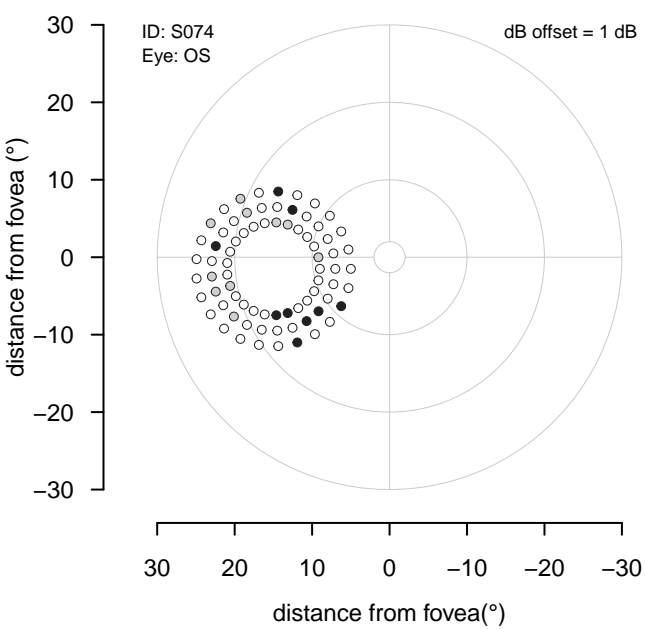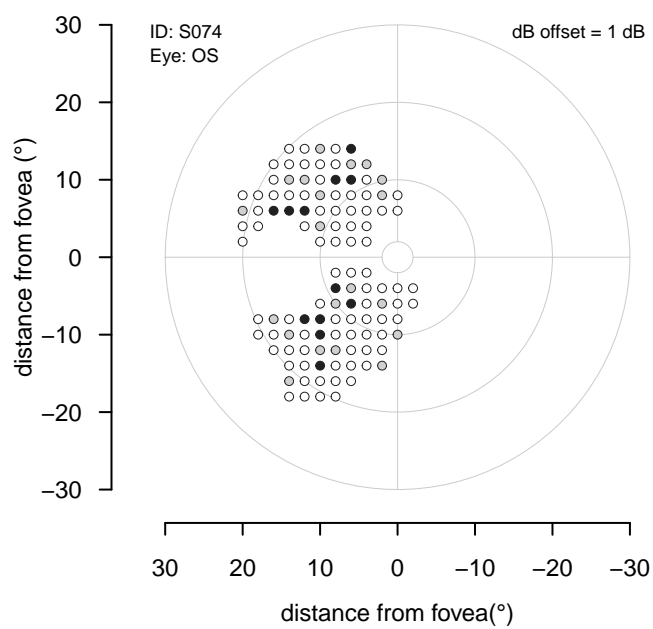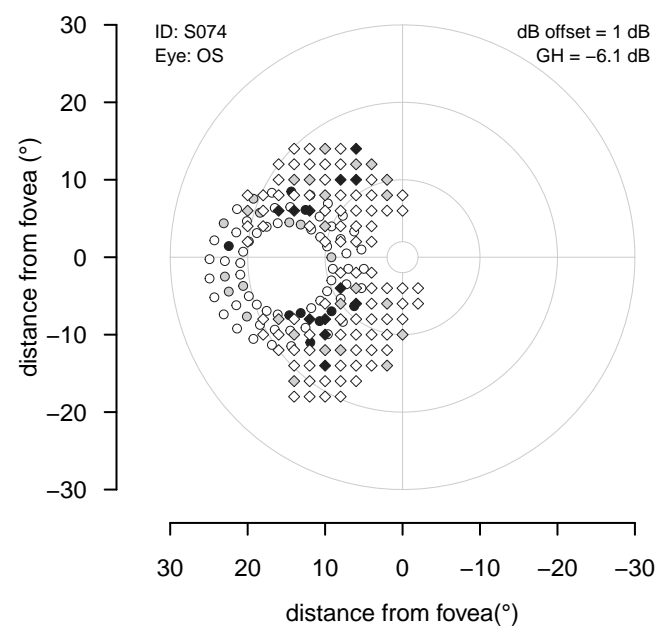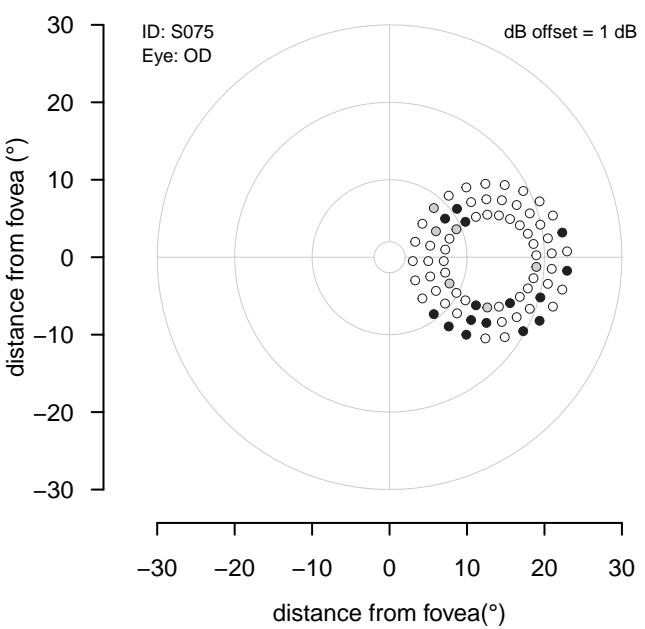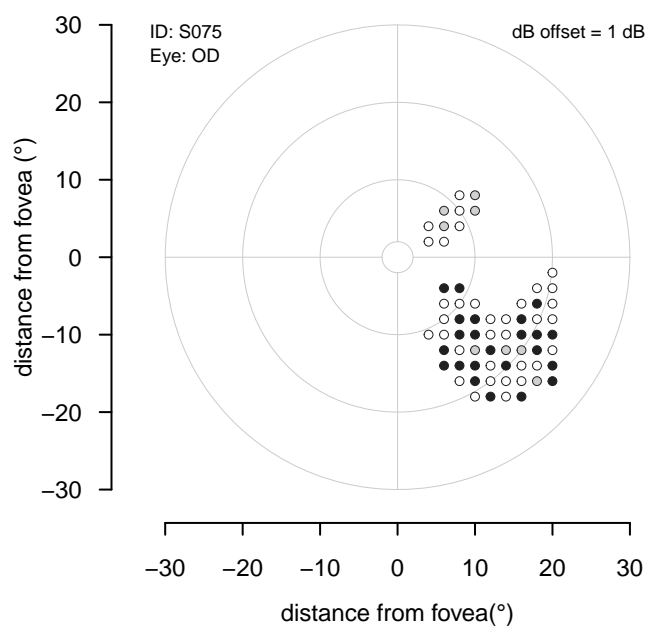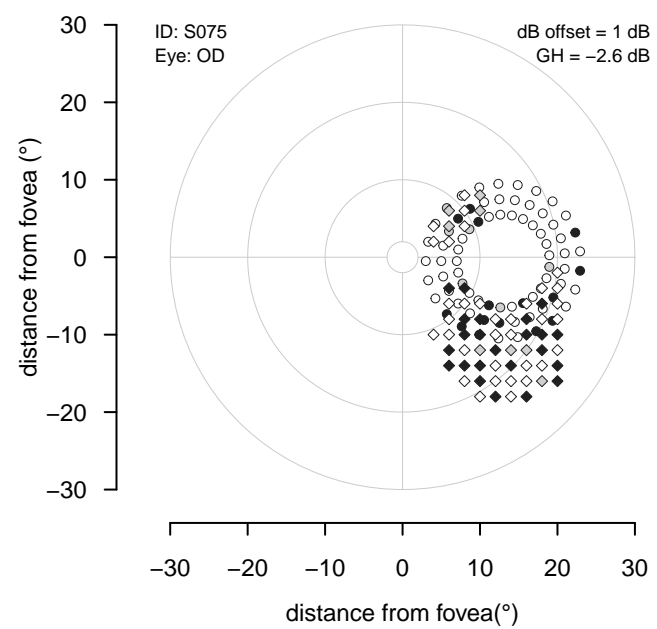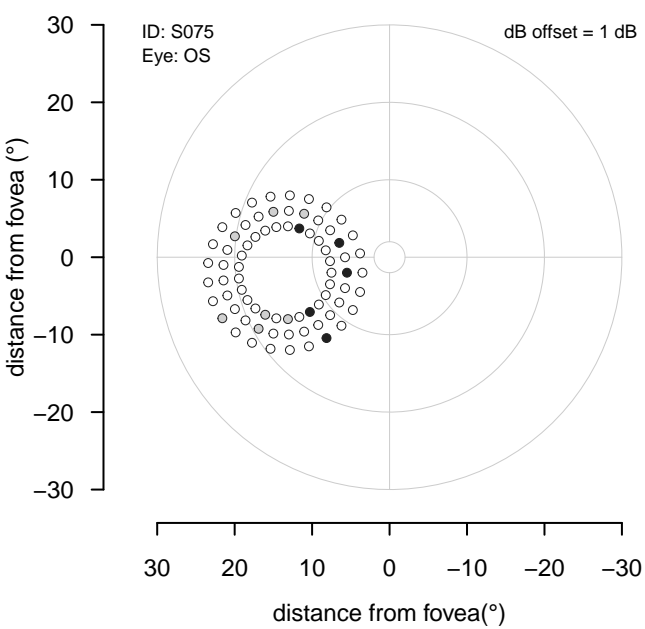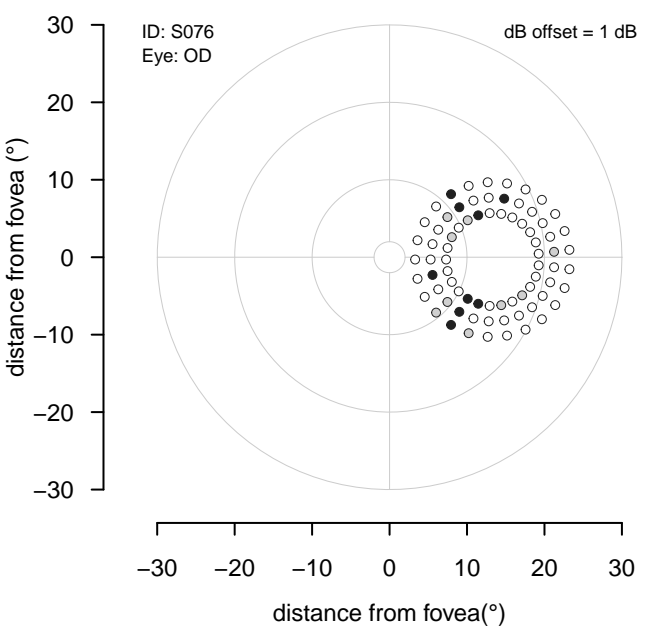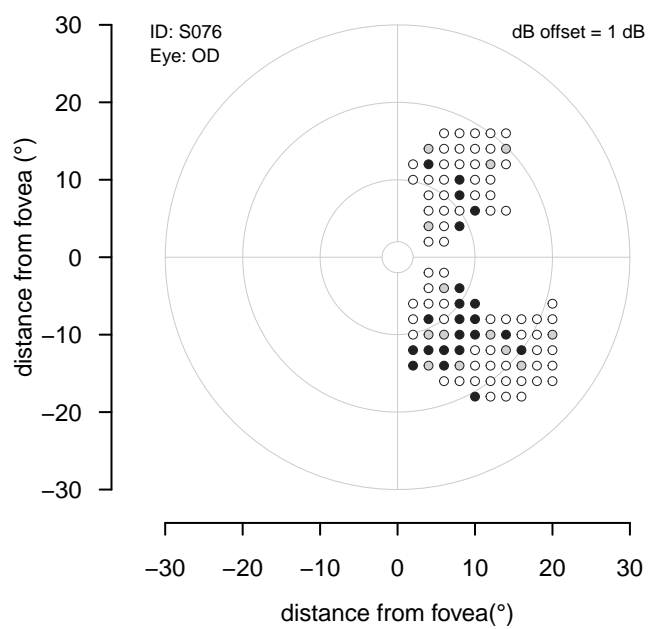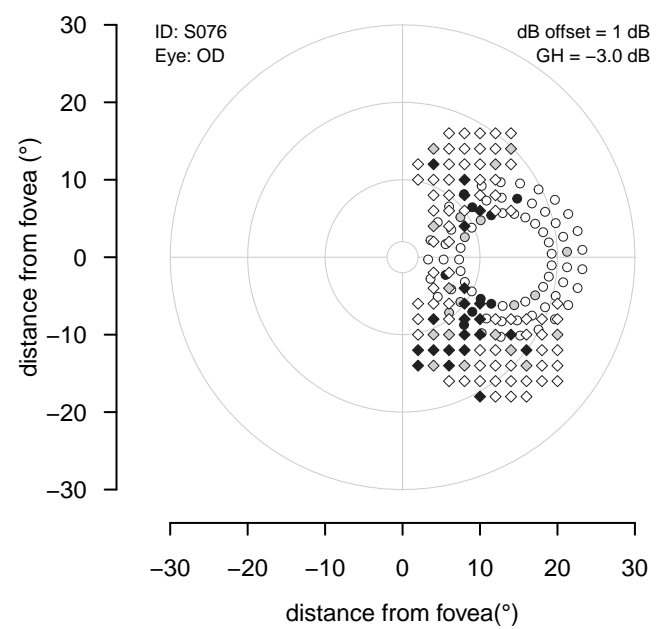

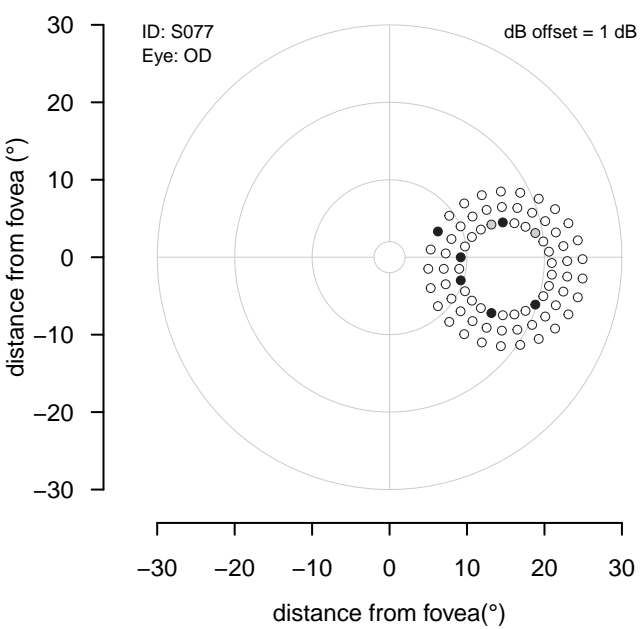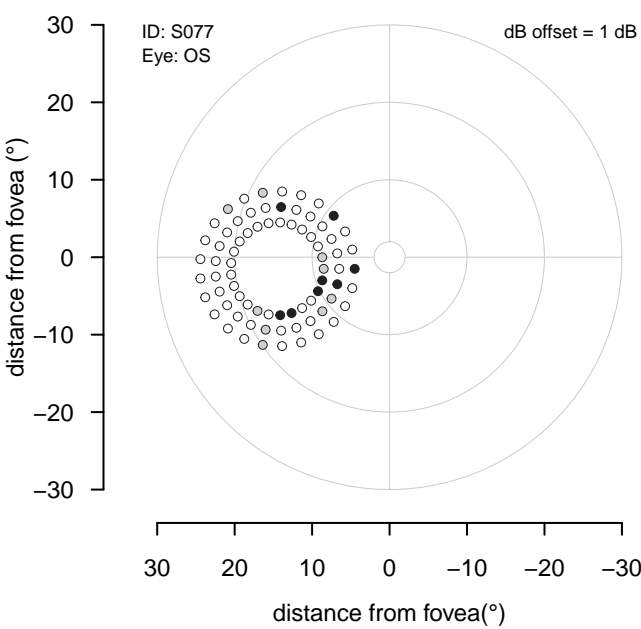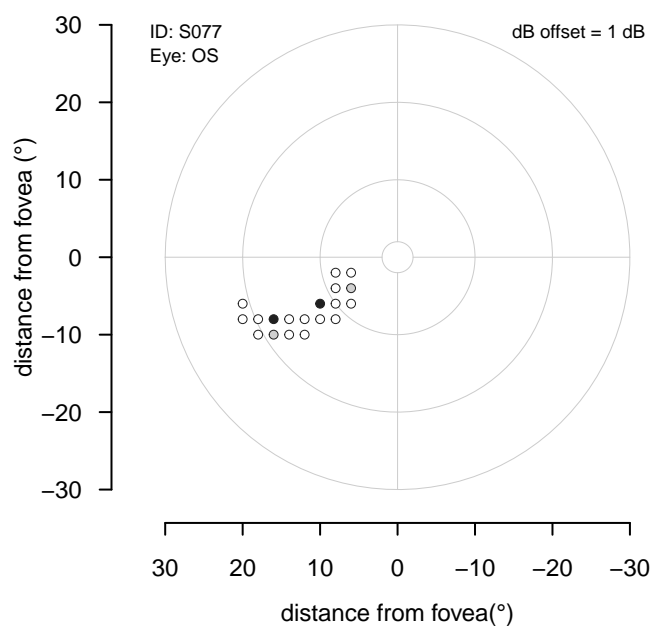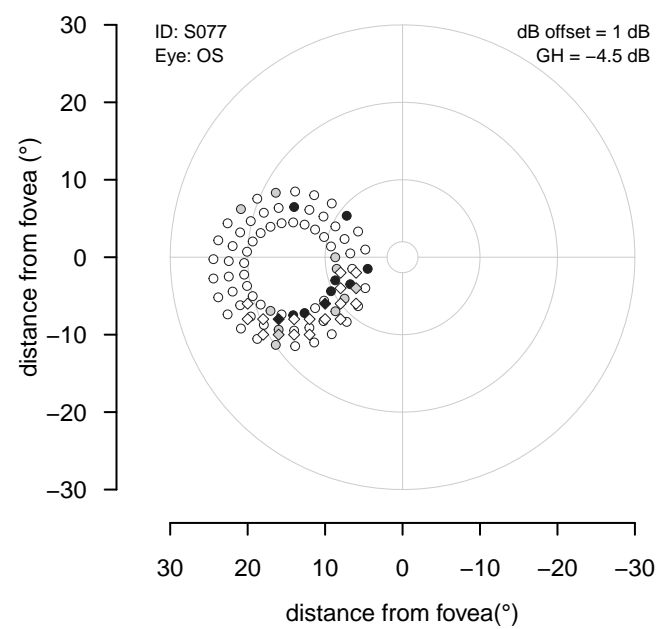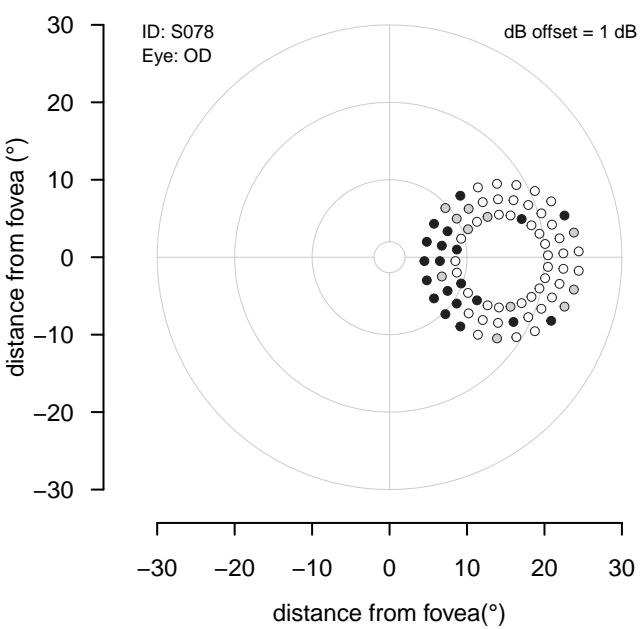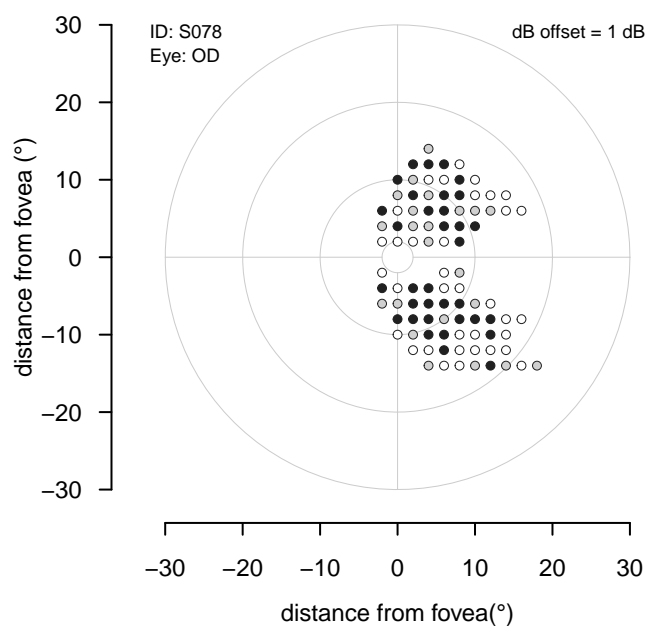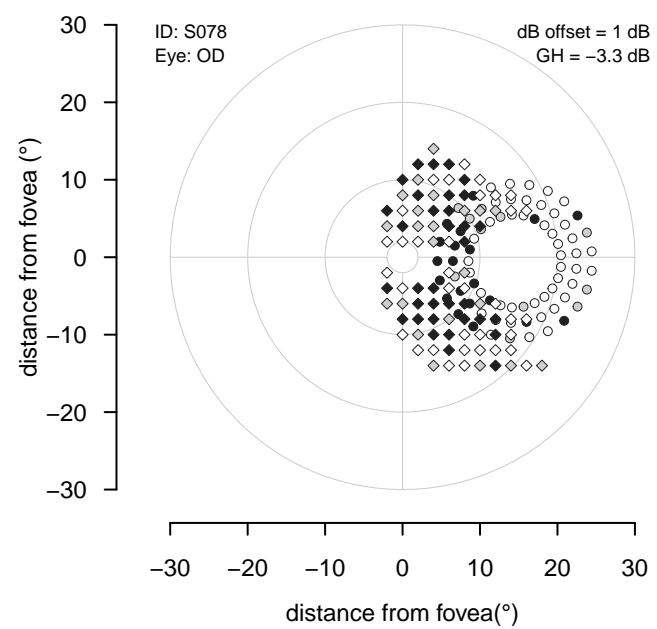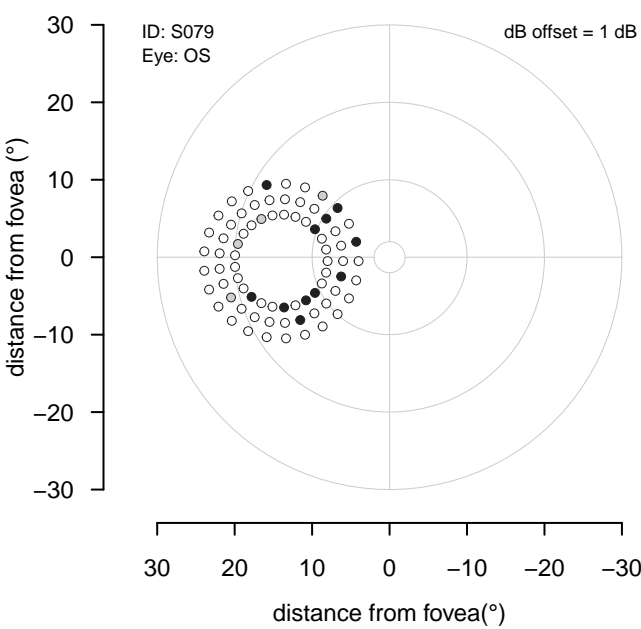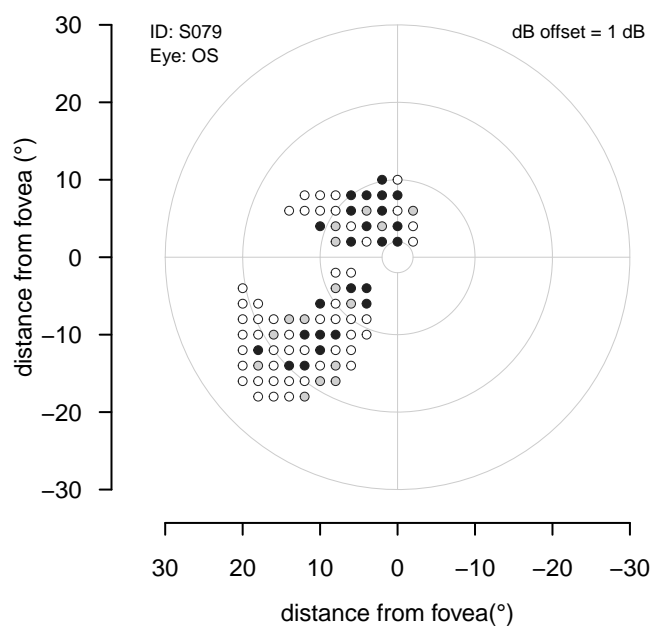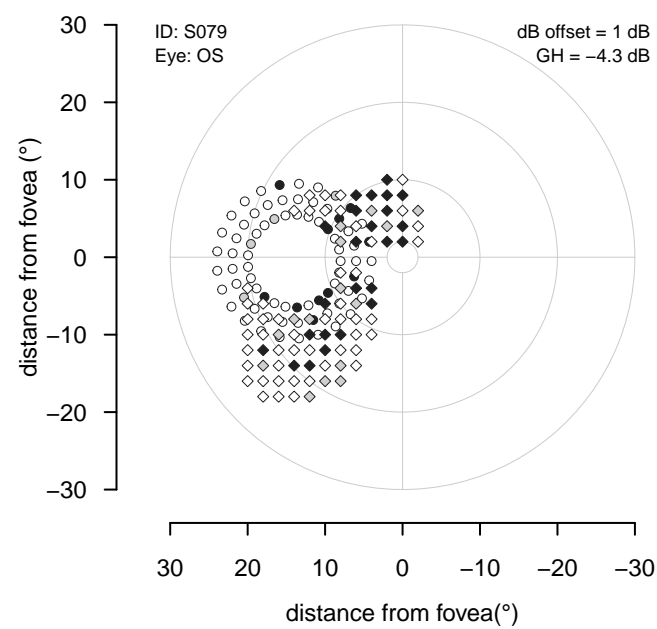

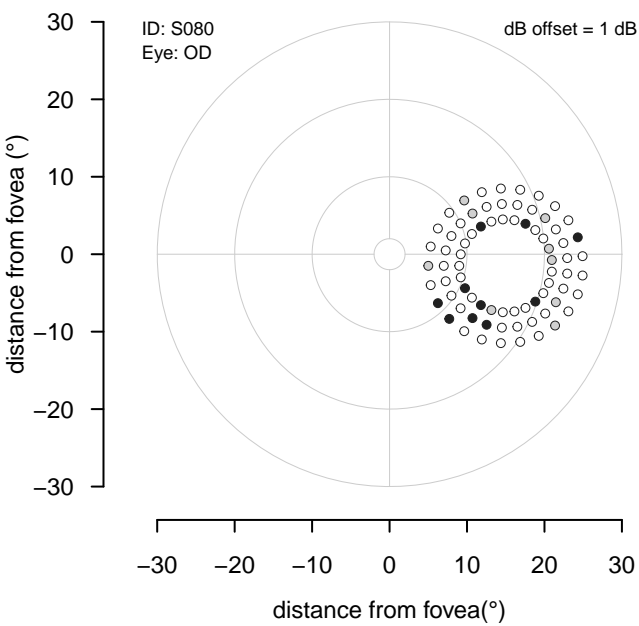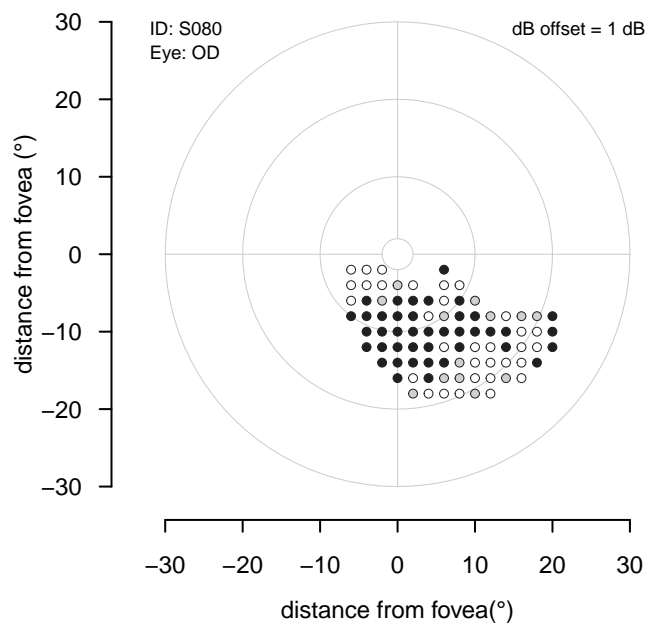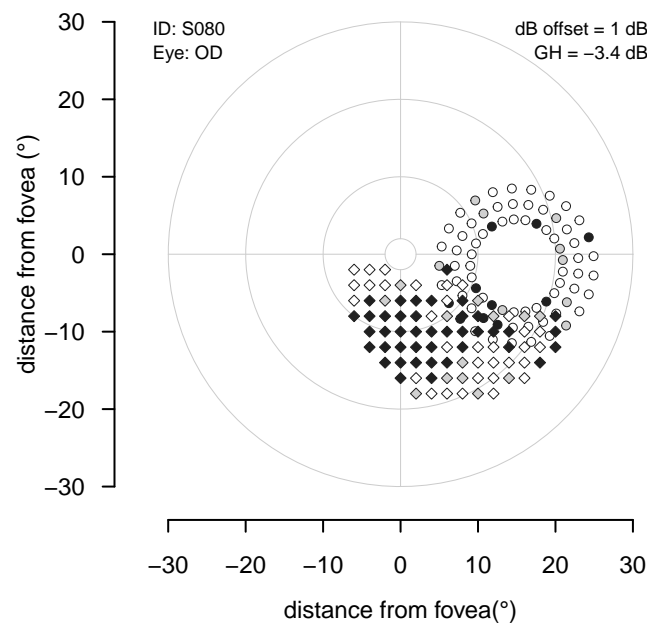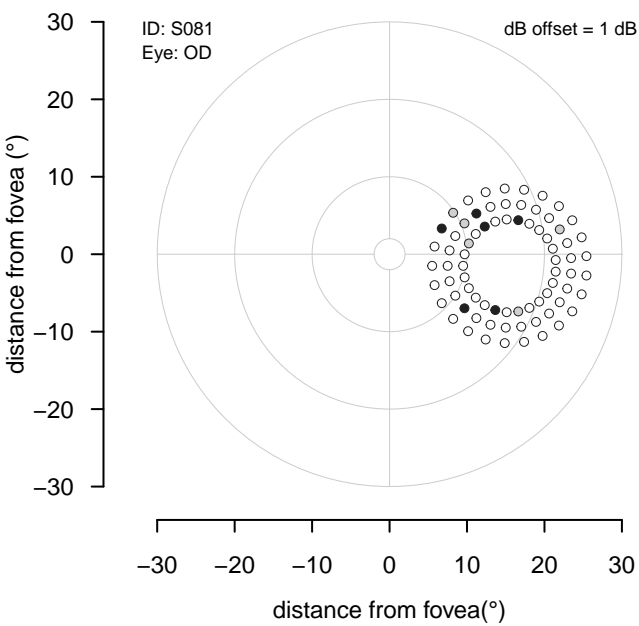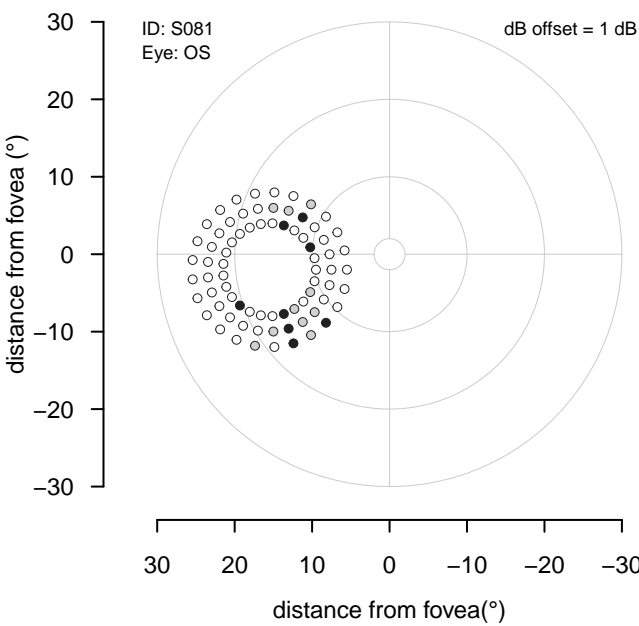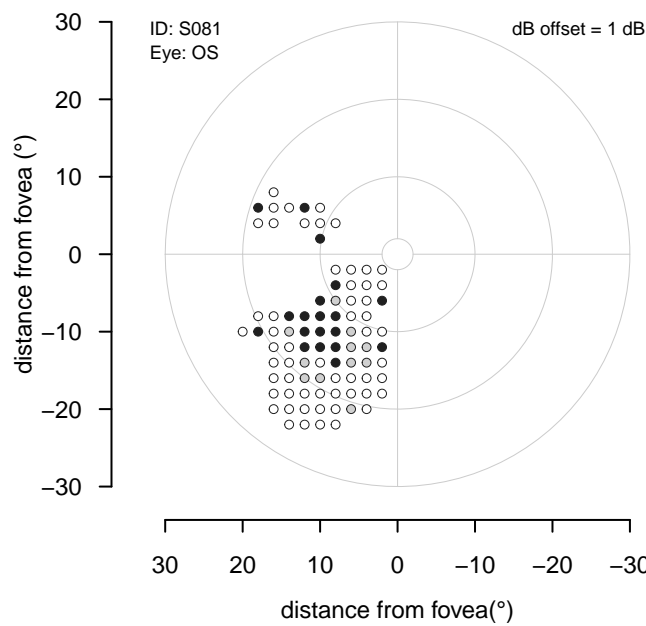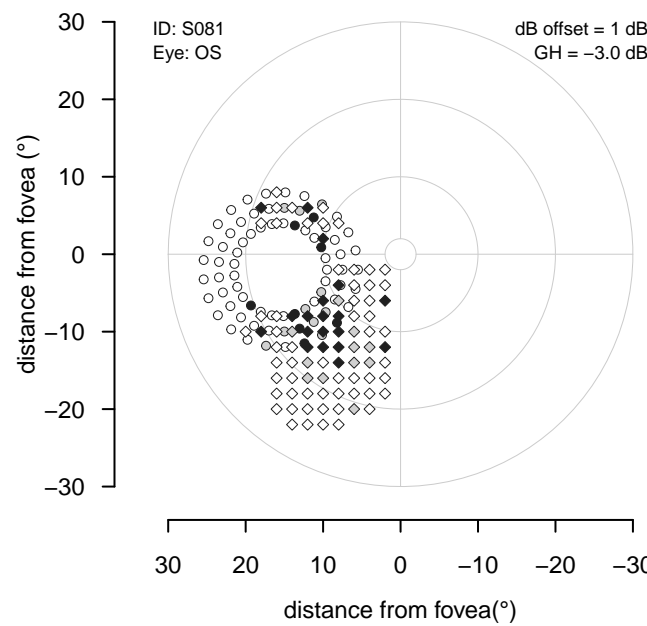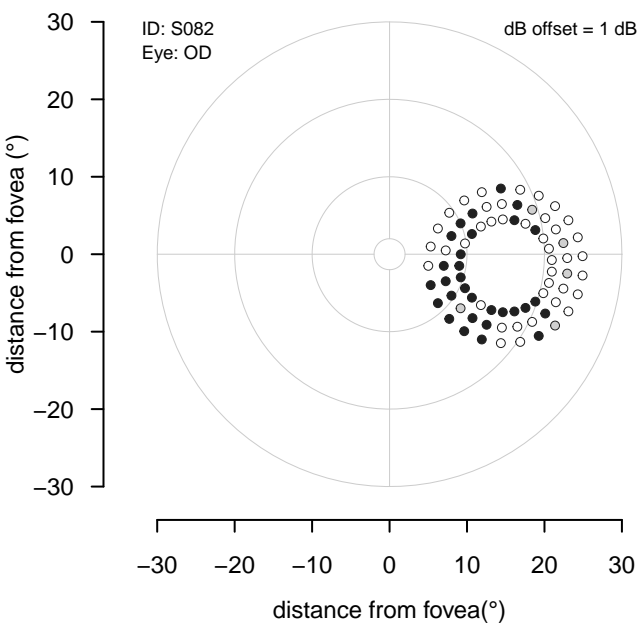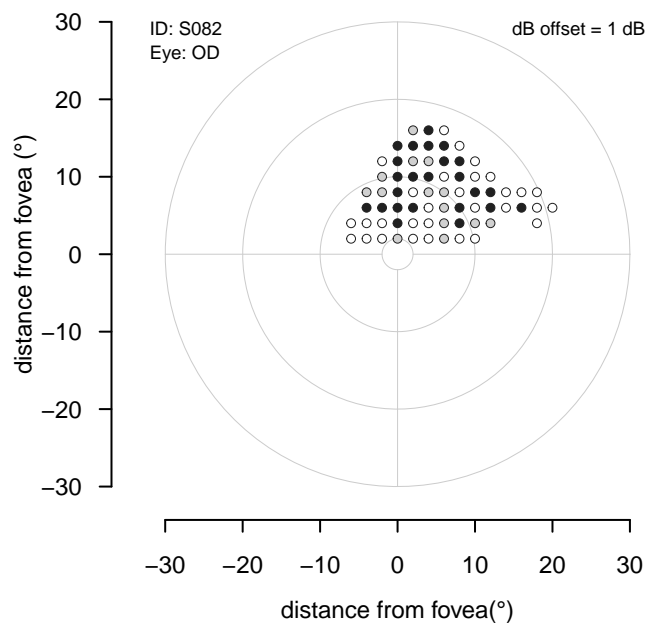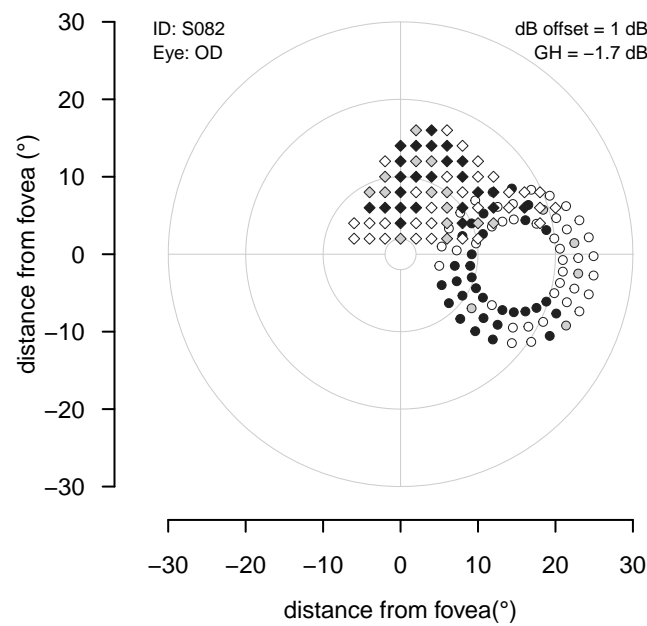

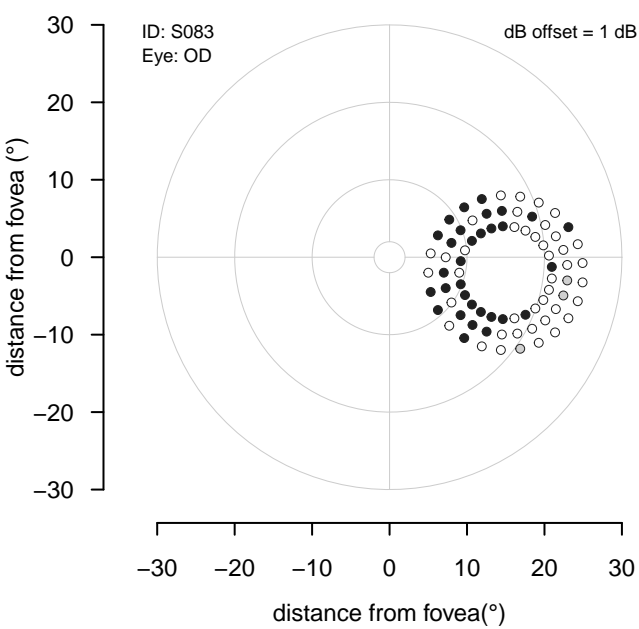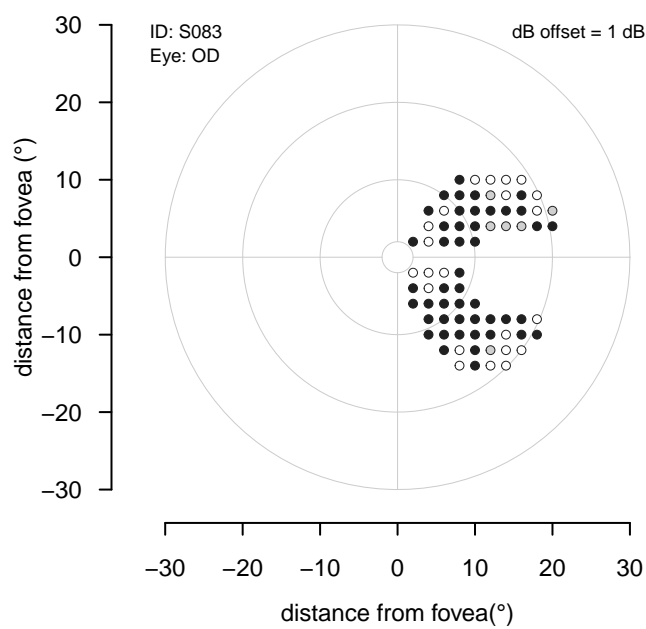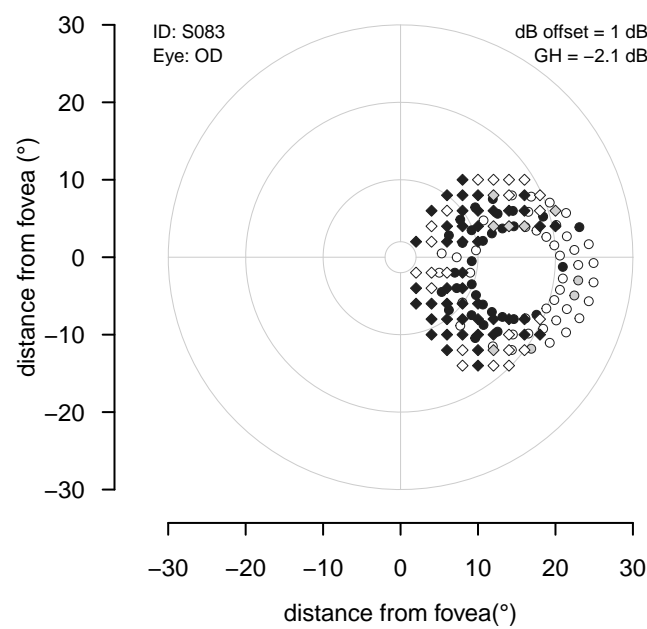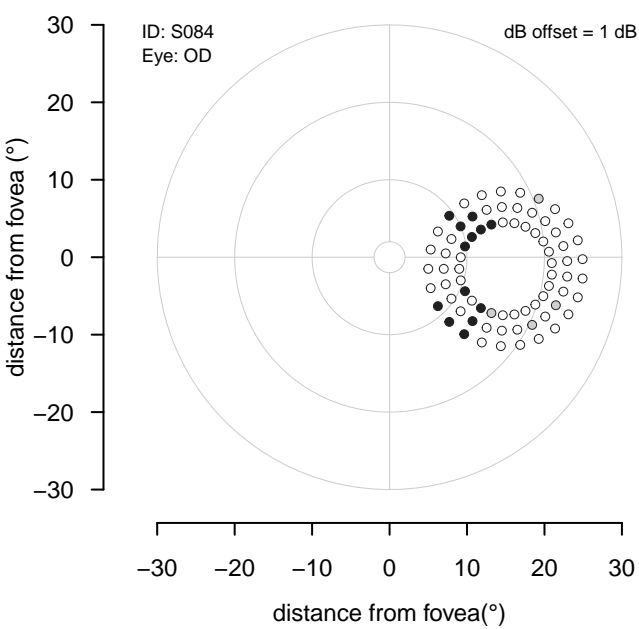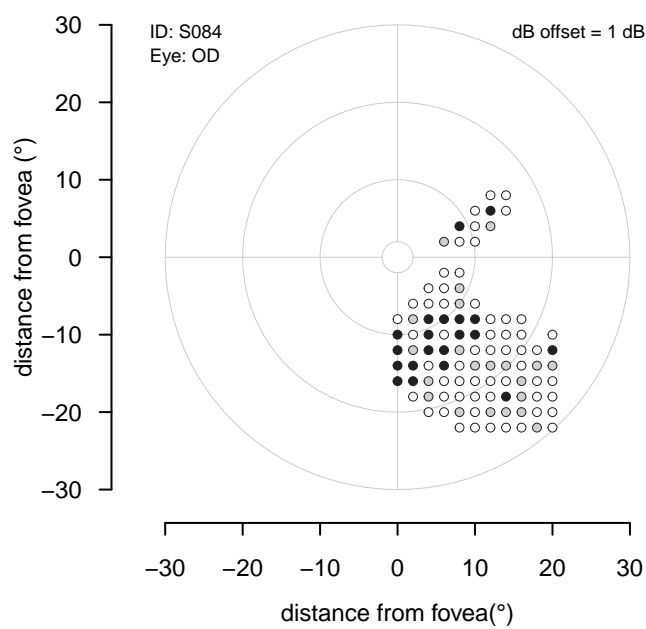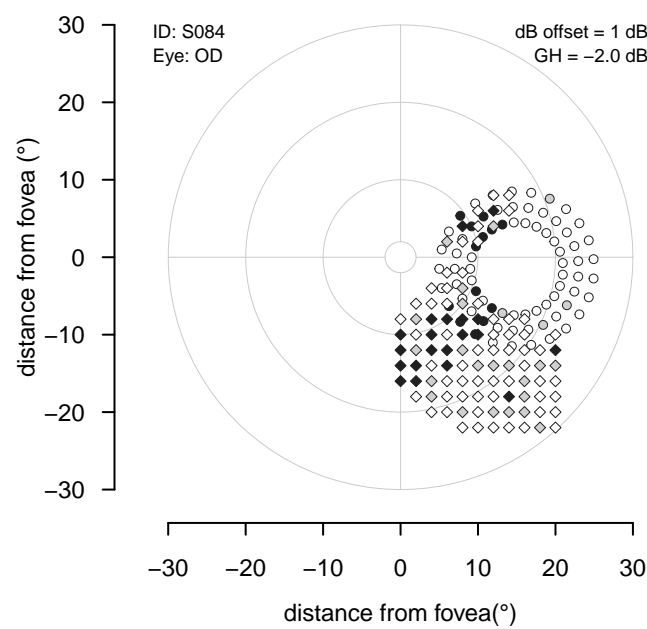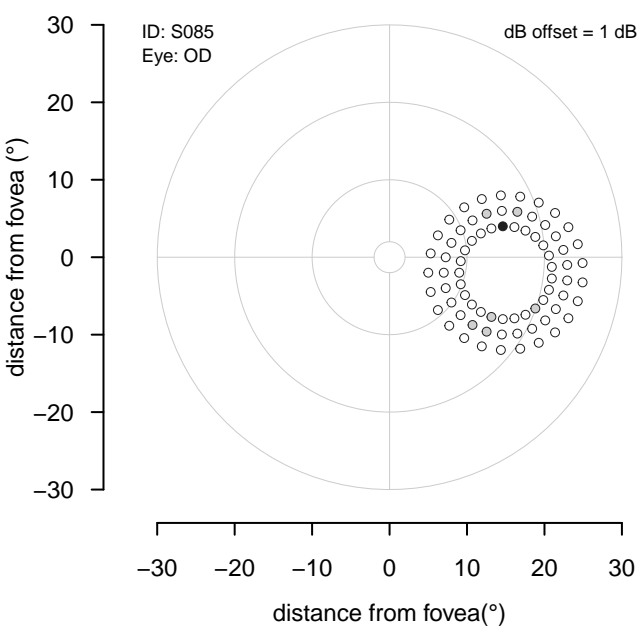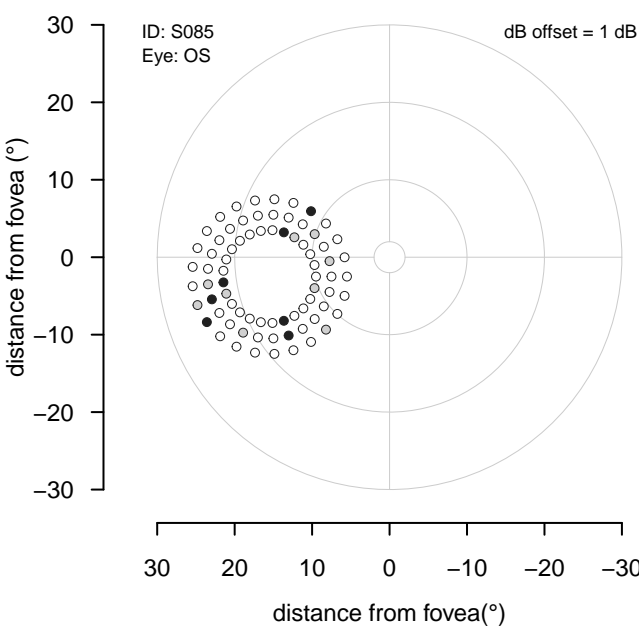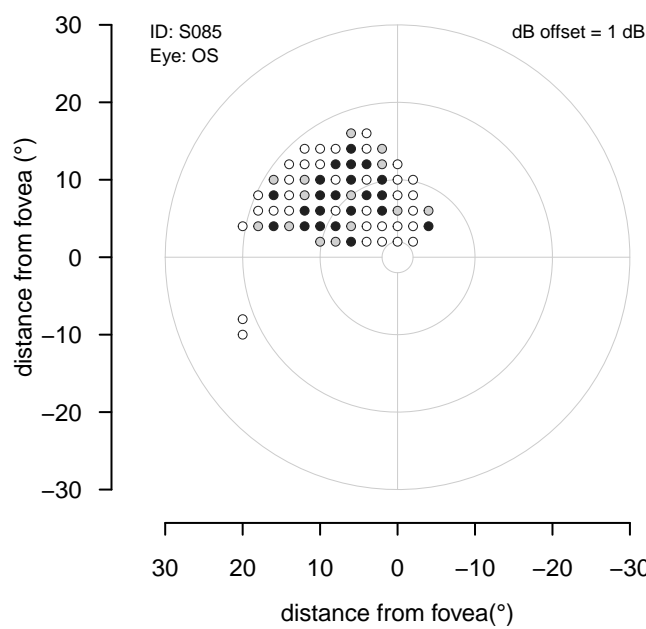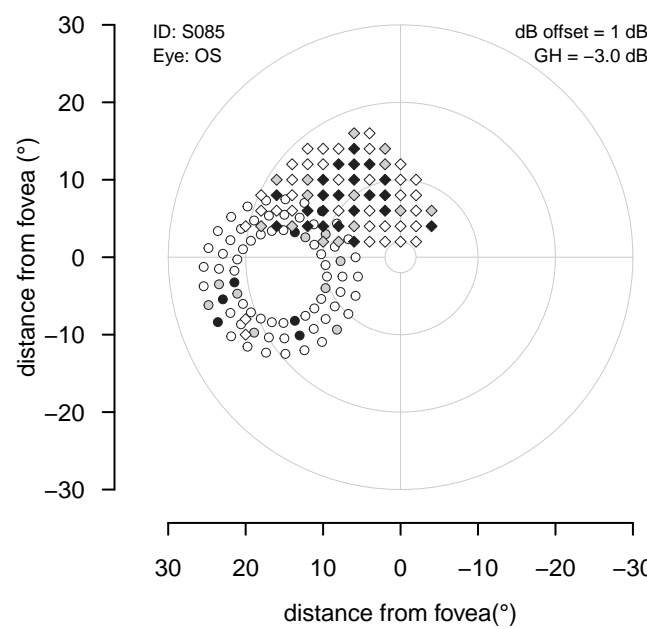

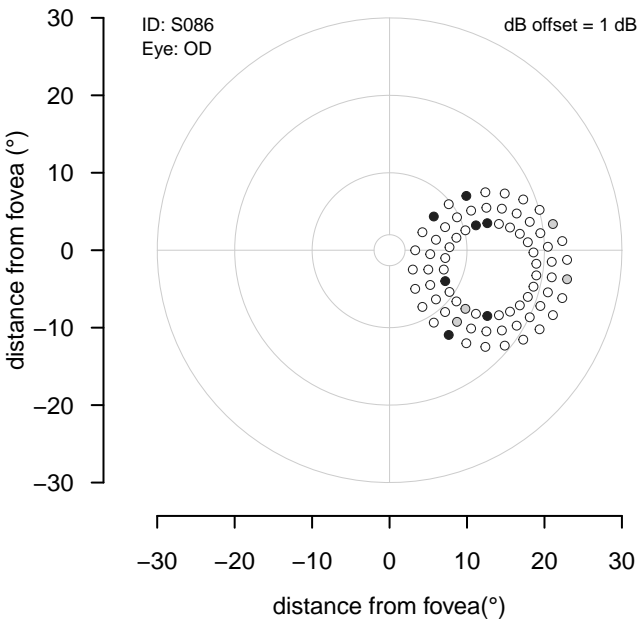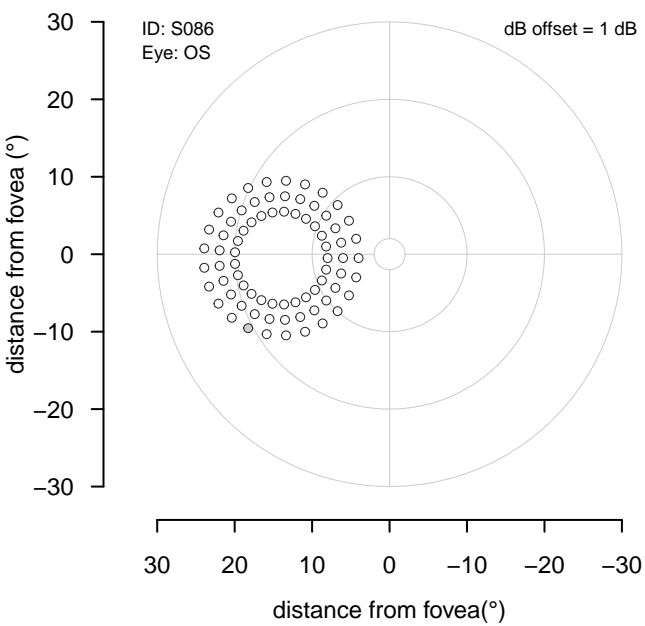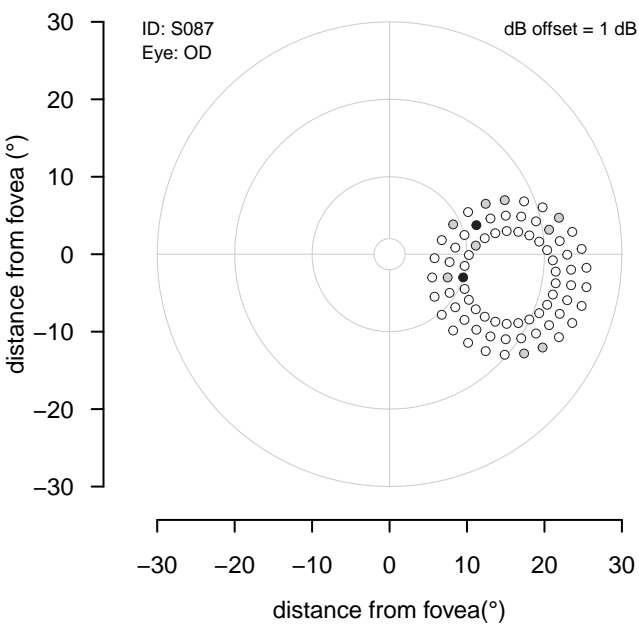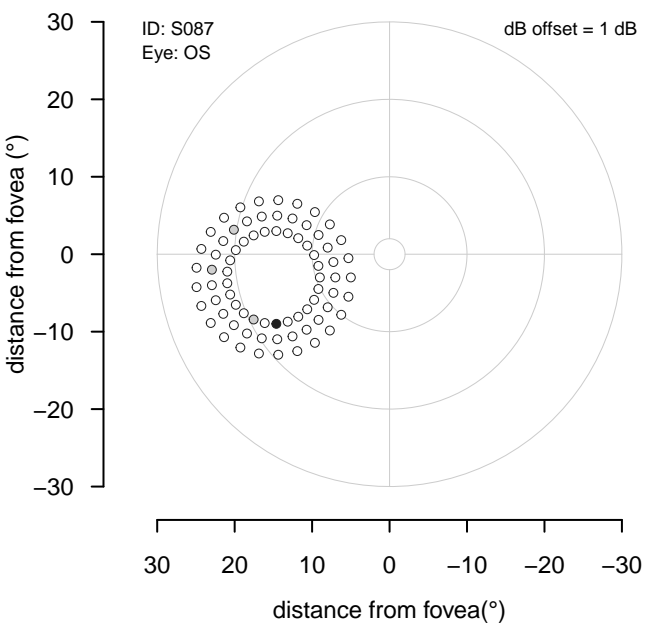

Supplement: Supplementary file 1 — Appendix S1. (ZIP 3.30 MB) [file 44402_2025_4507042_MOESM1_ESM.zip › opo70014-sup-0005-Supinfo5.pdf]

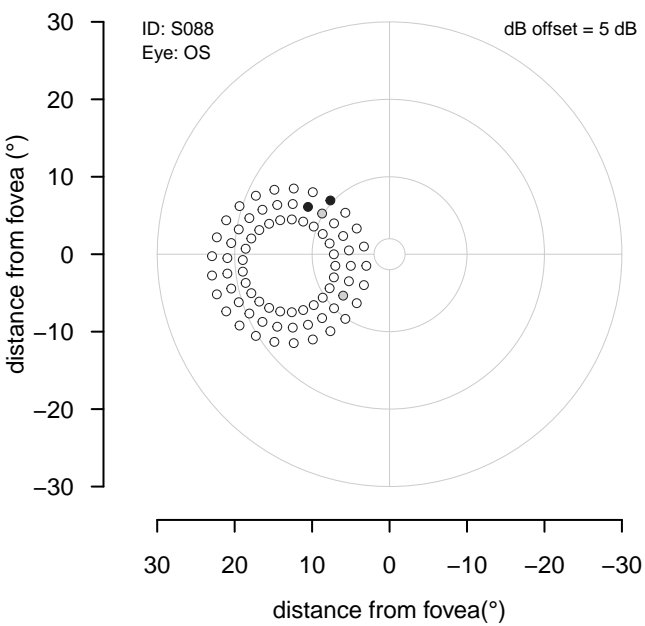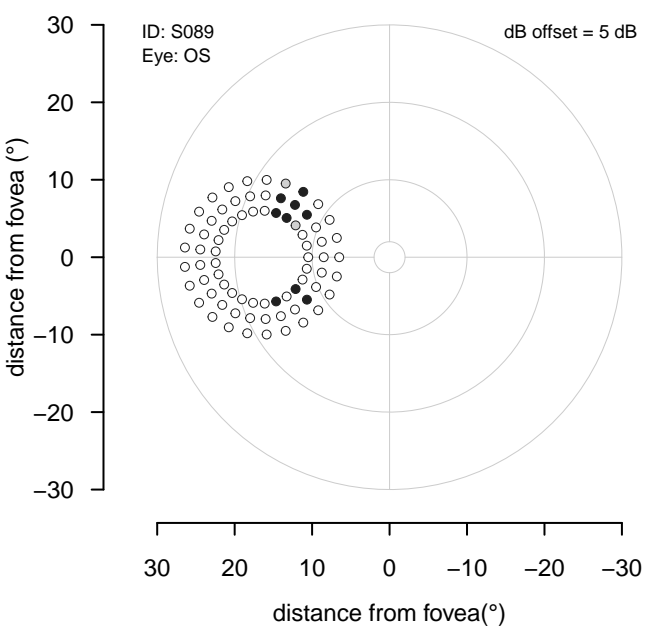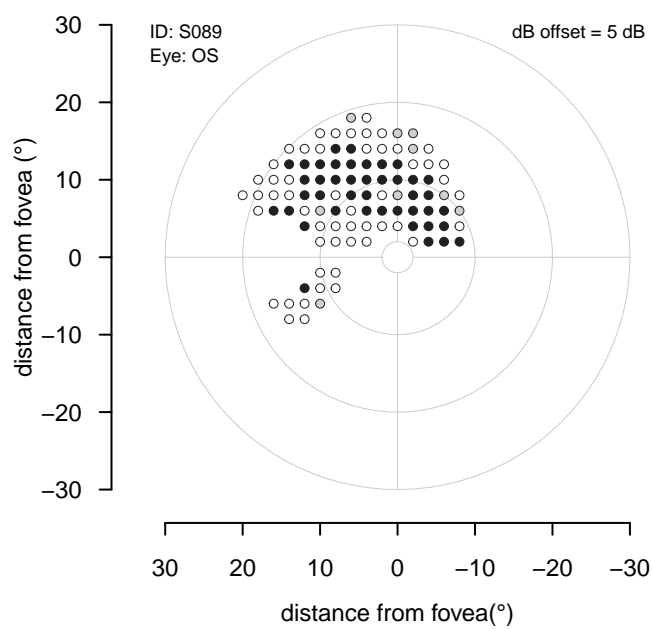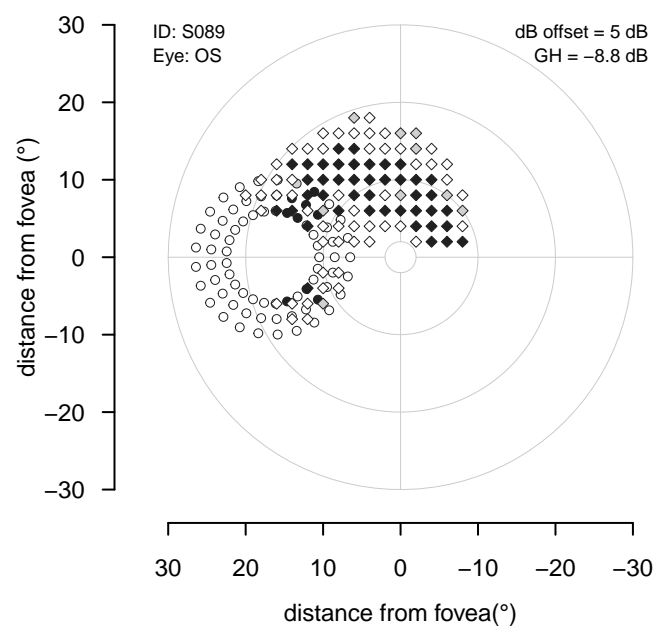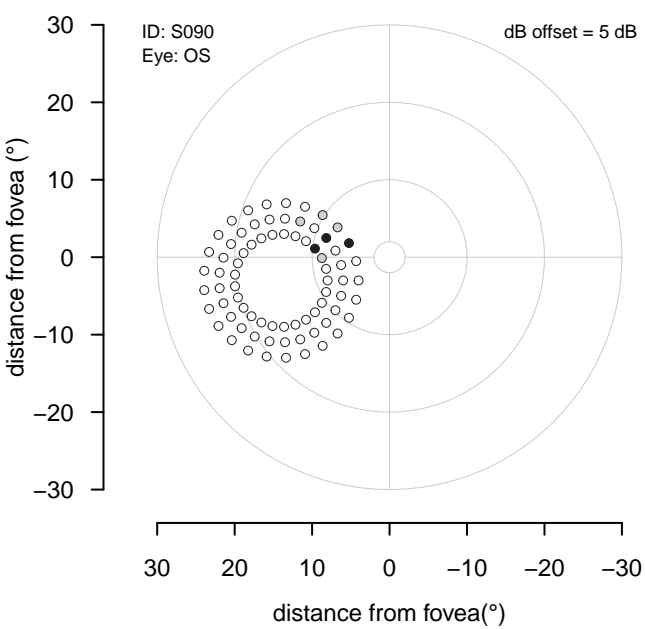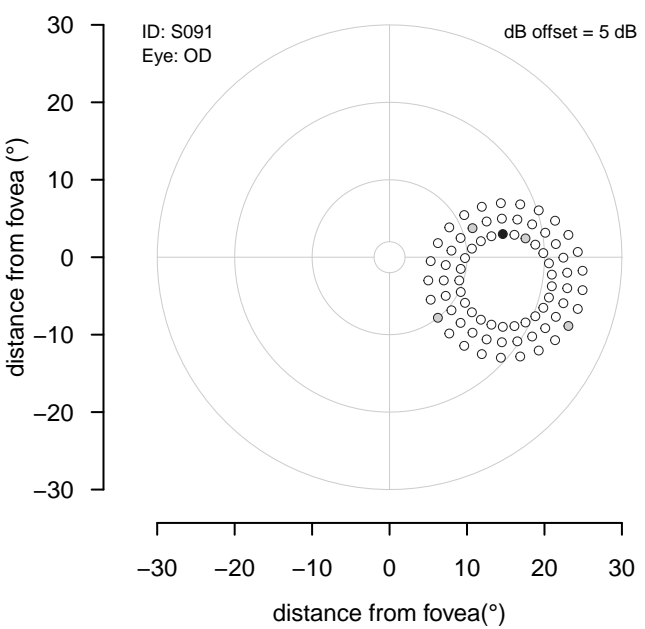

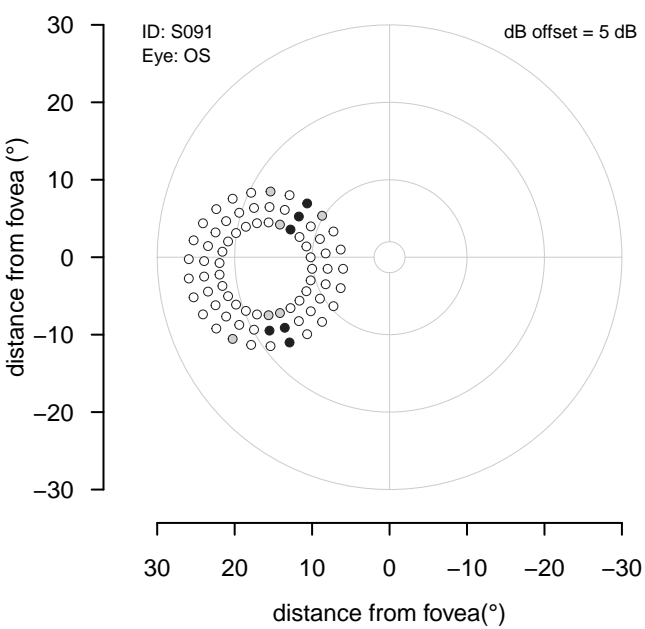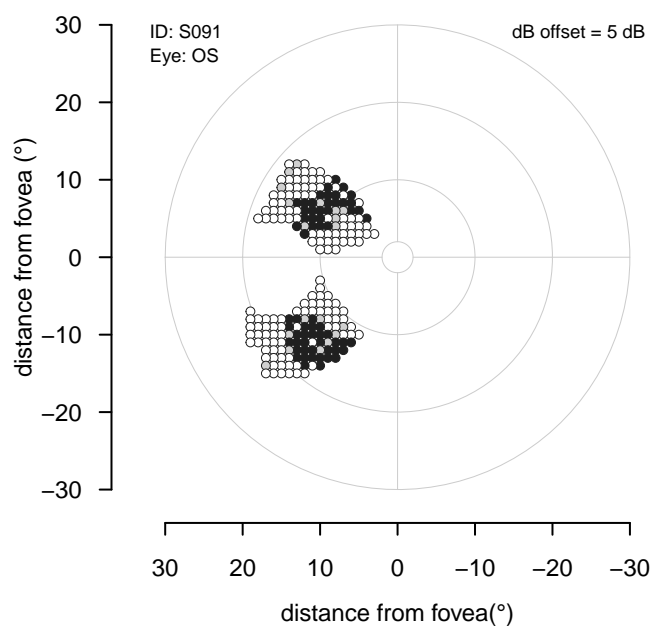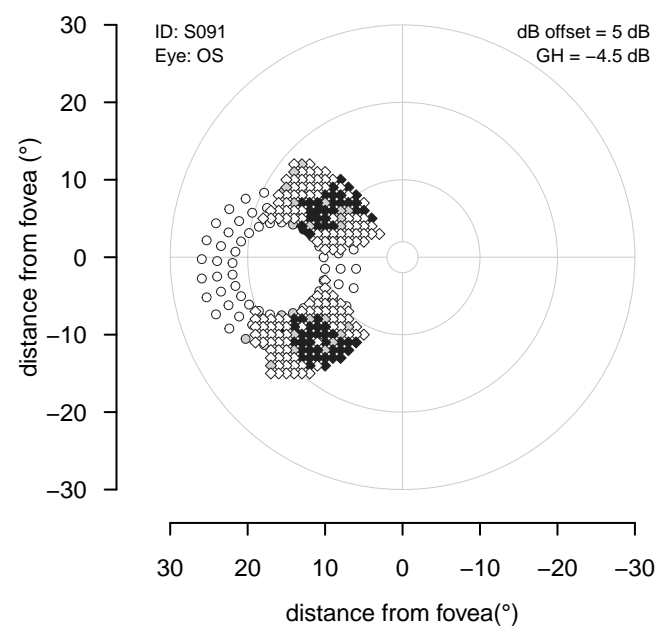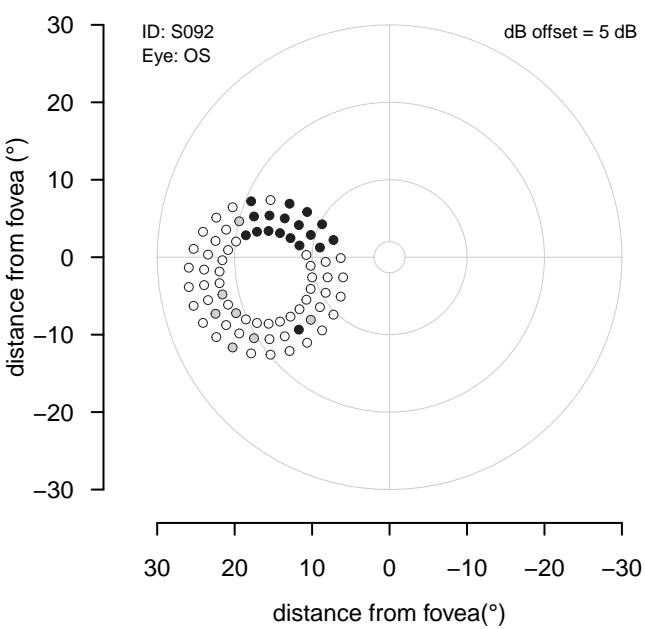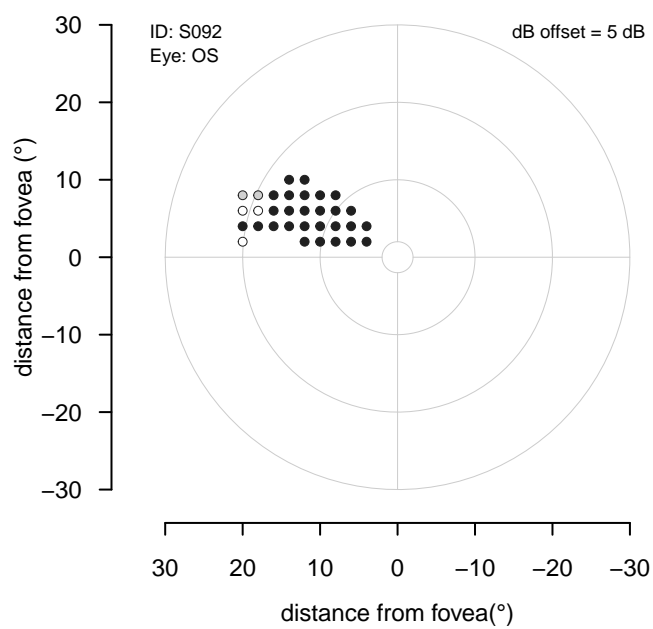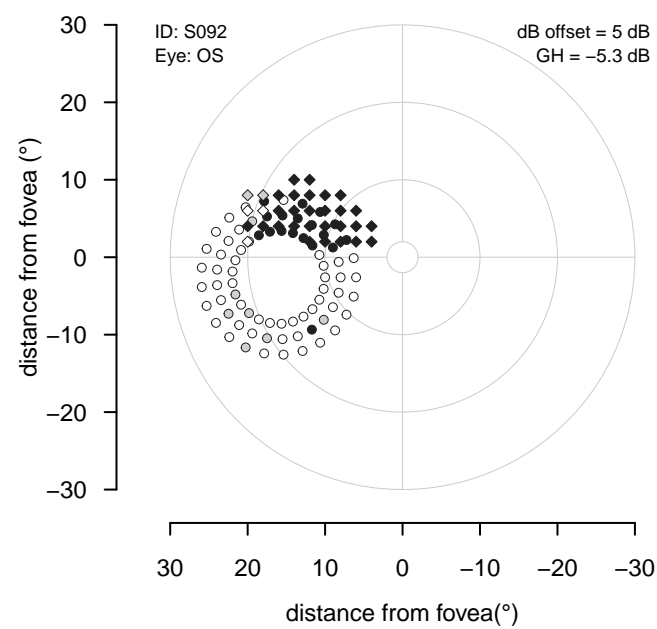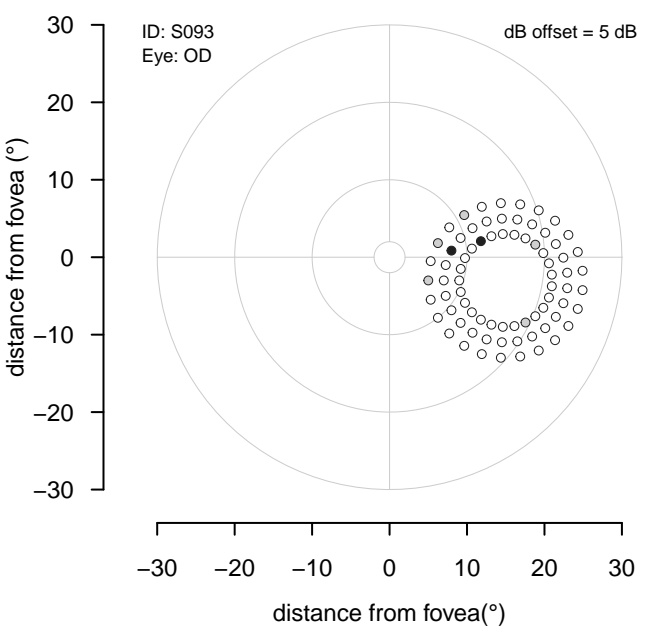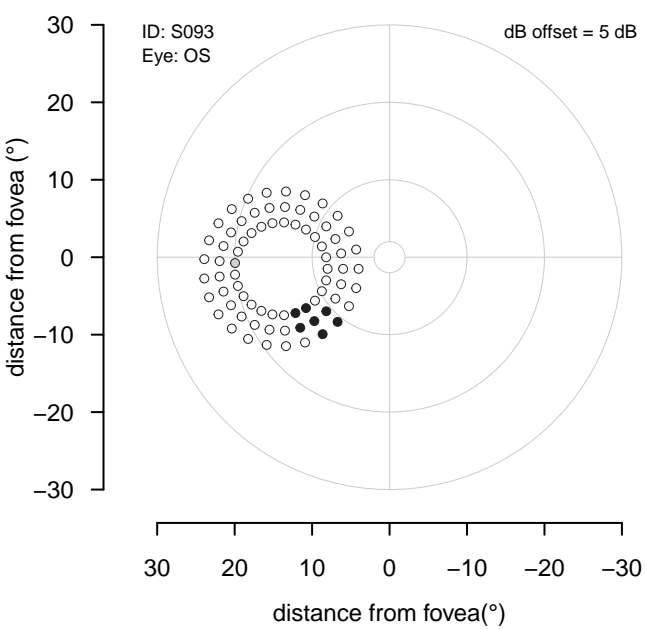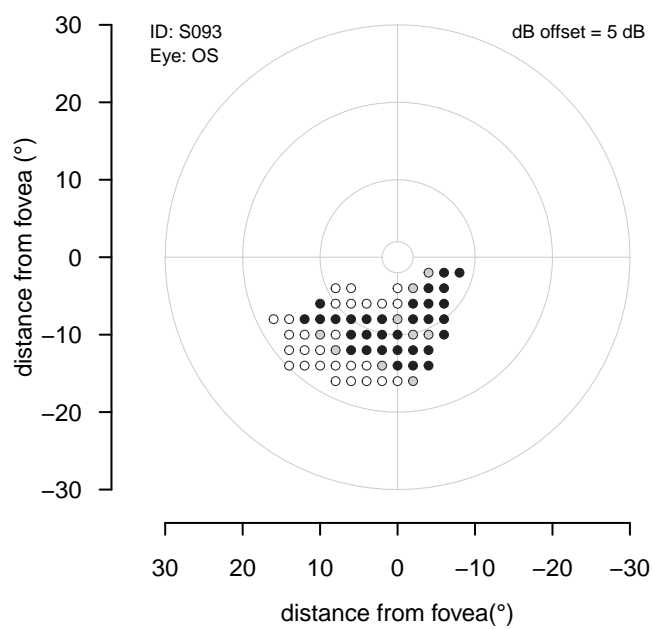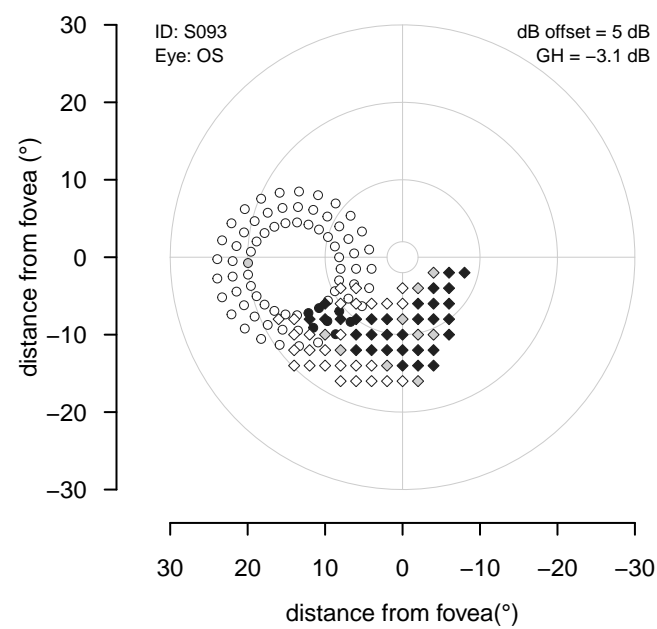

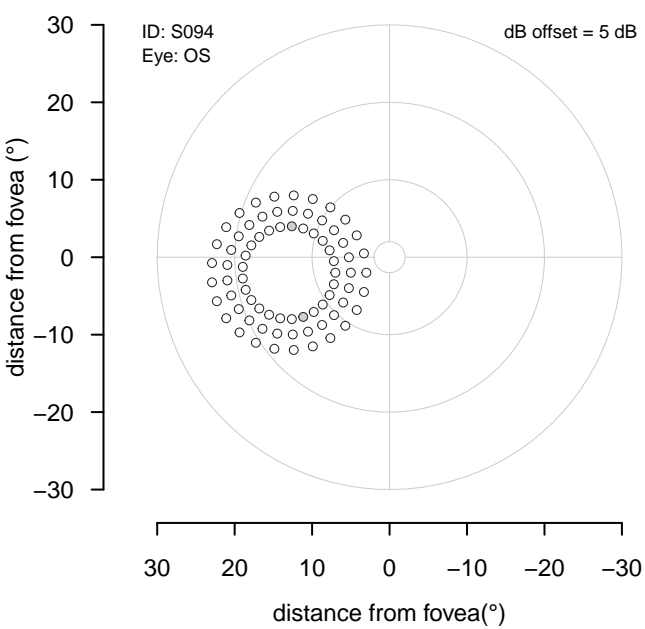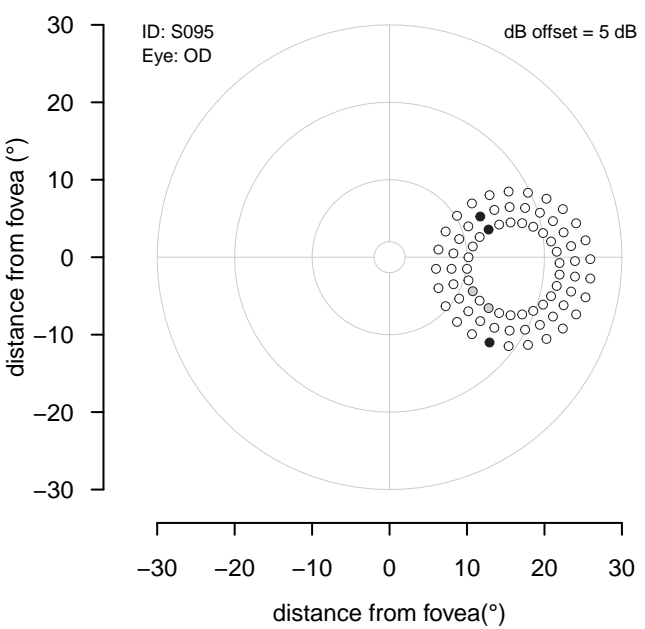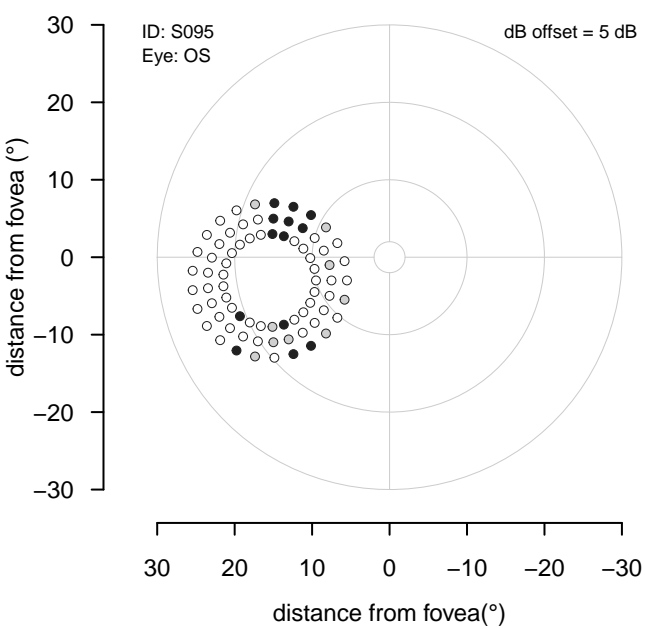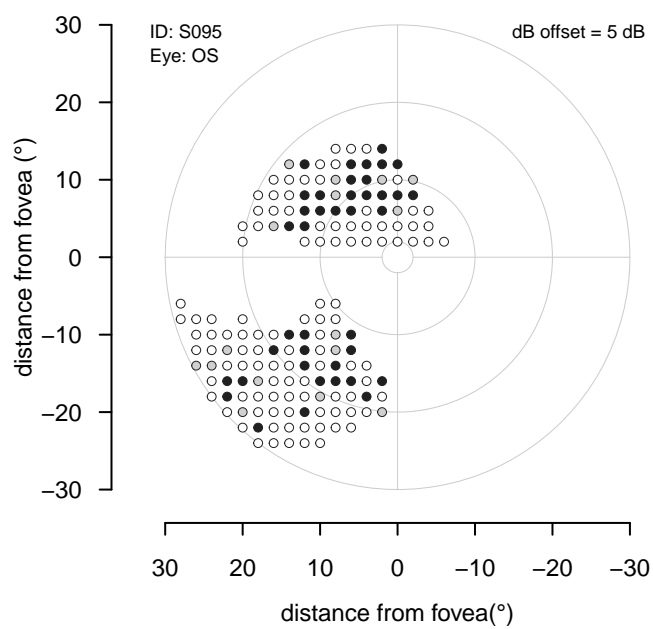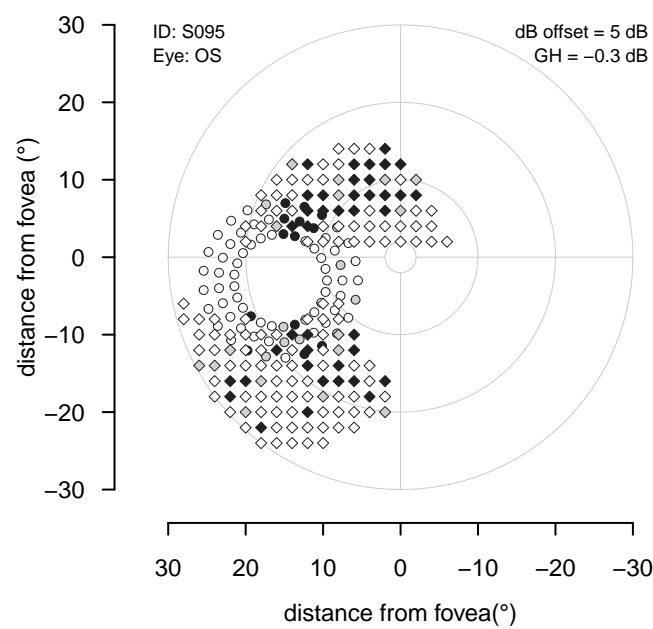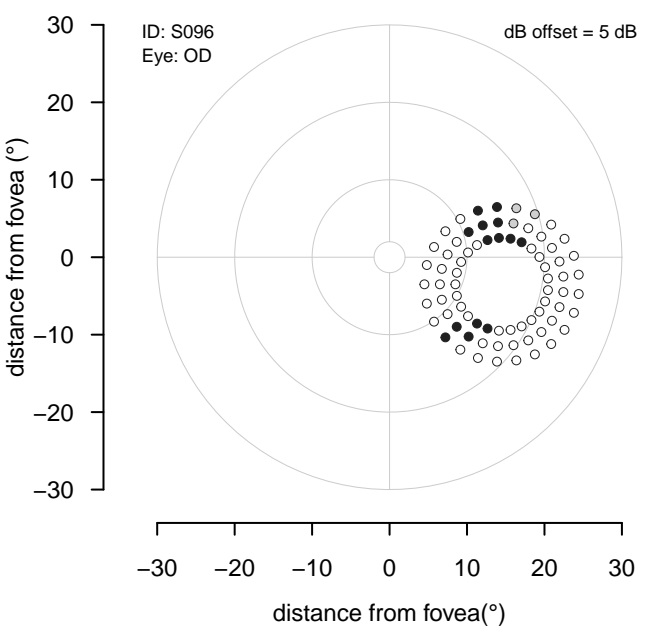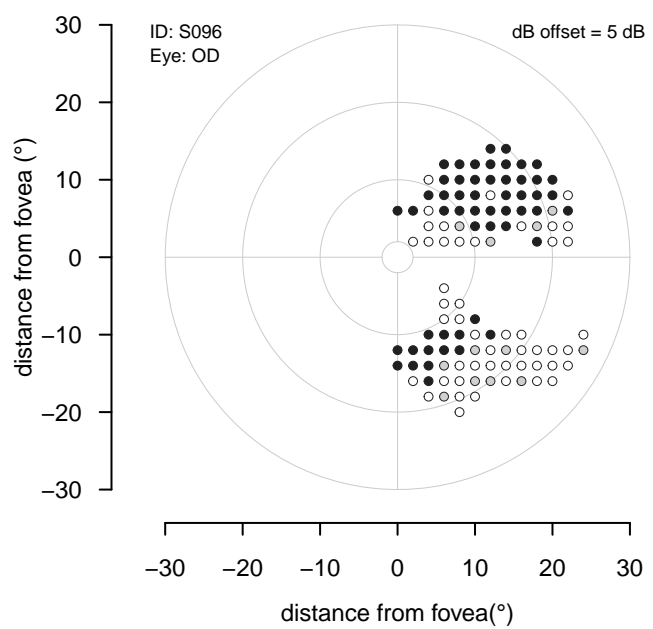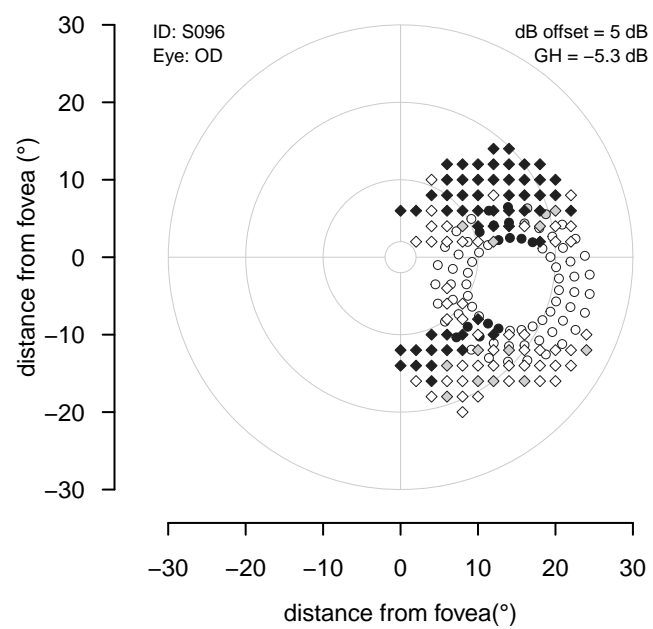

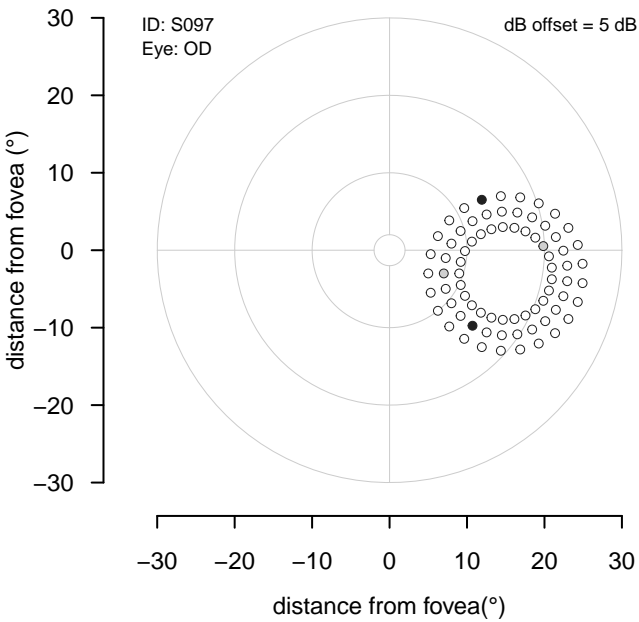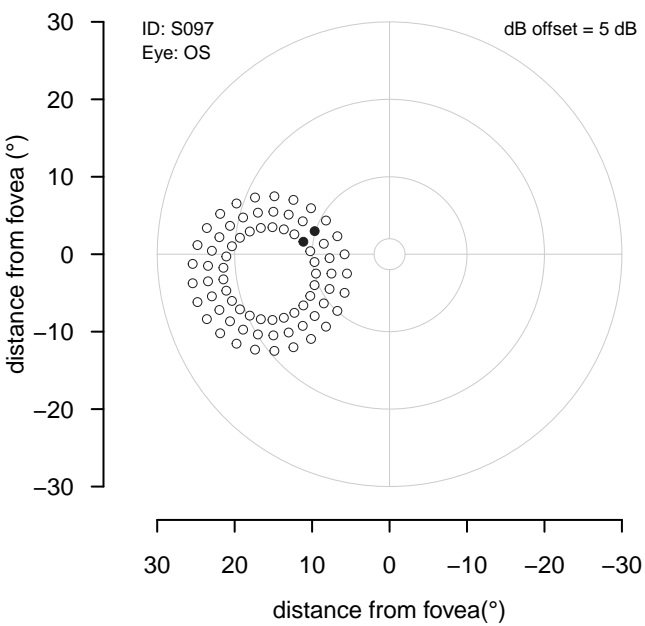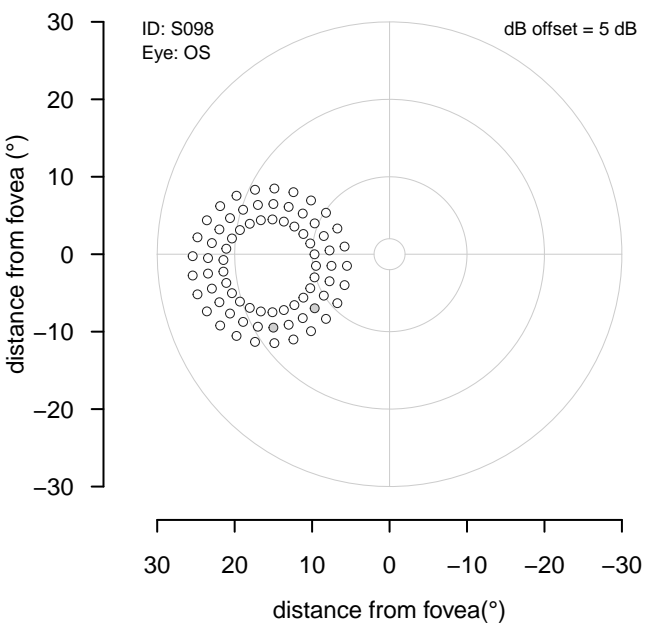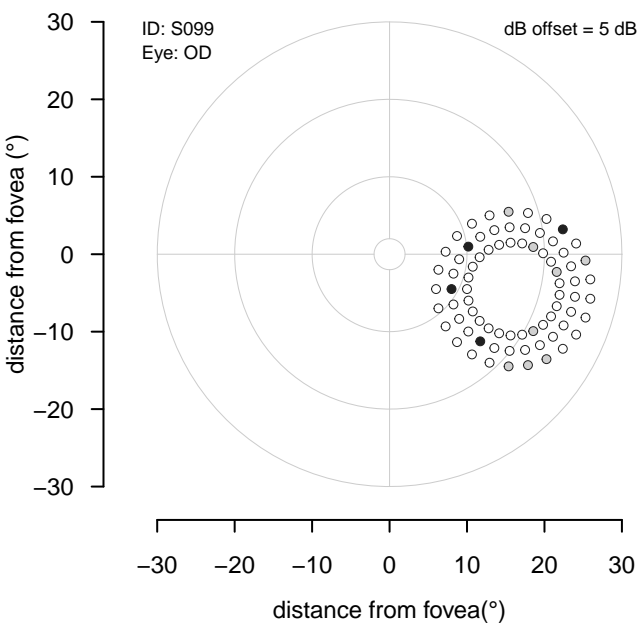

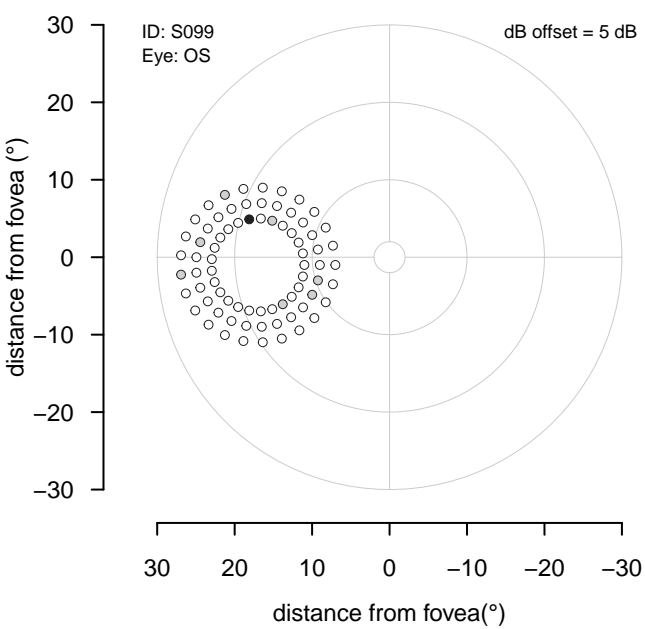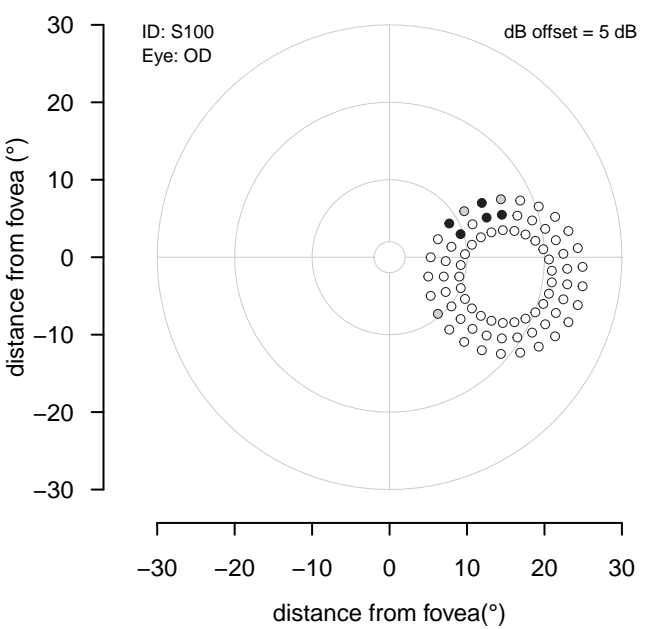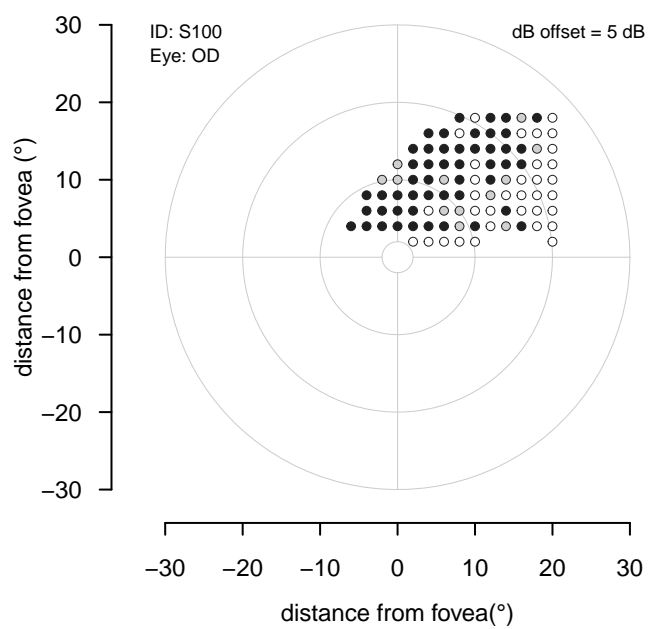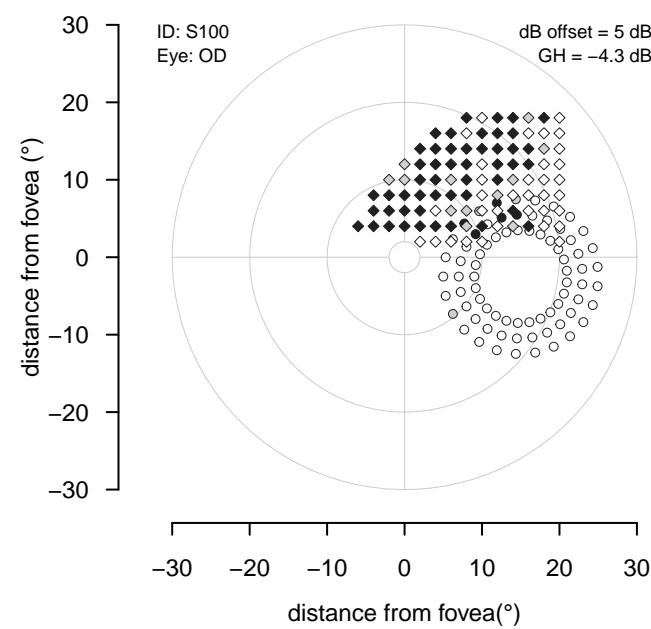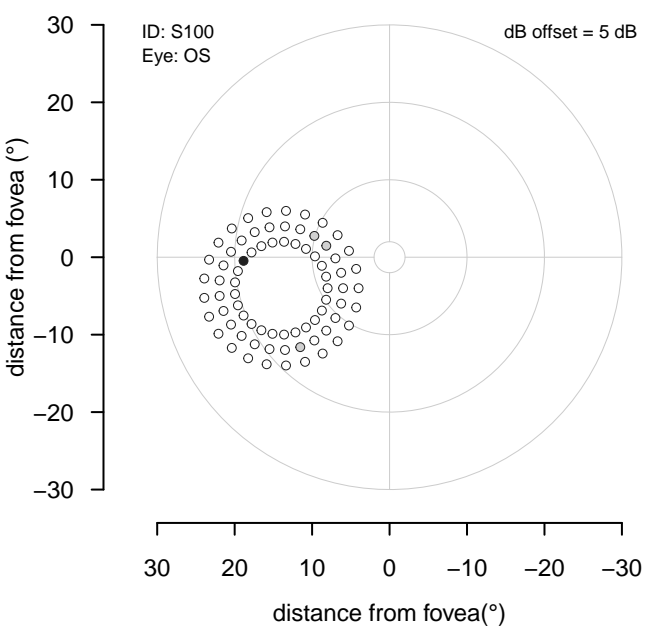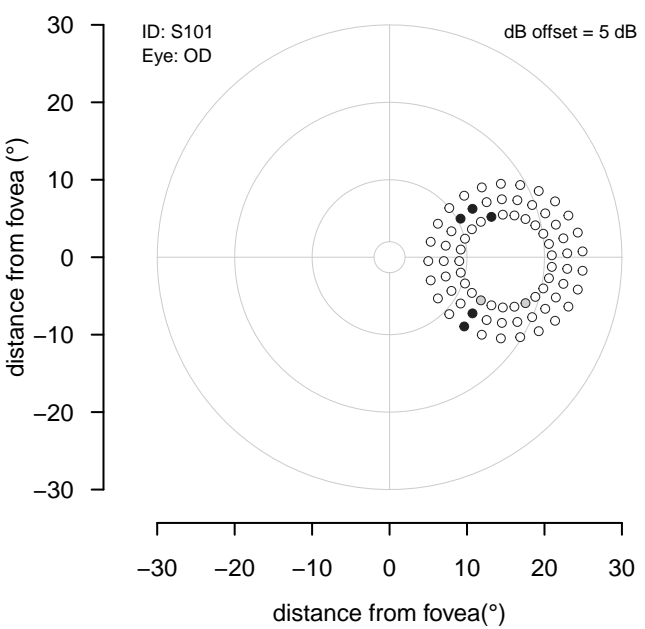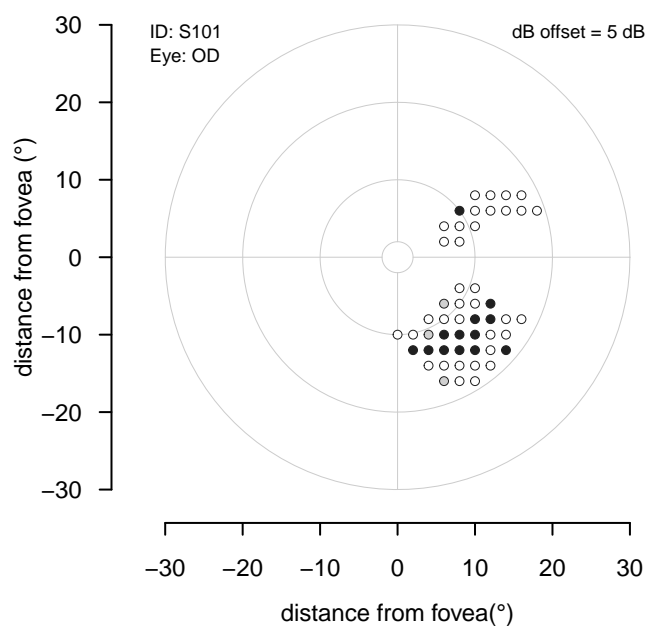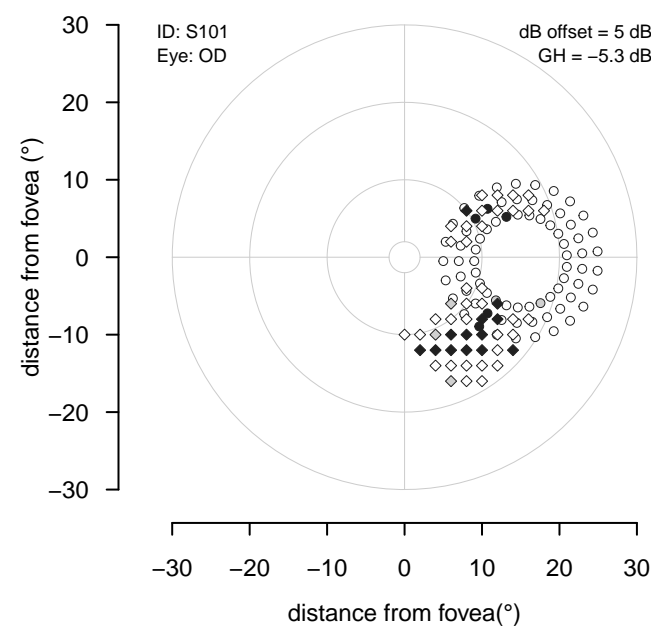

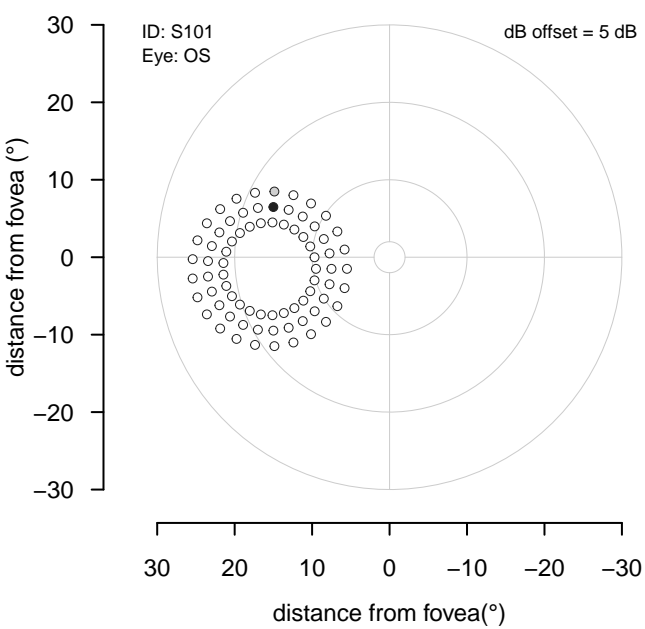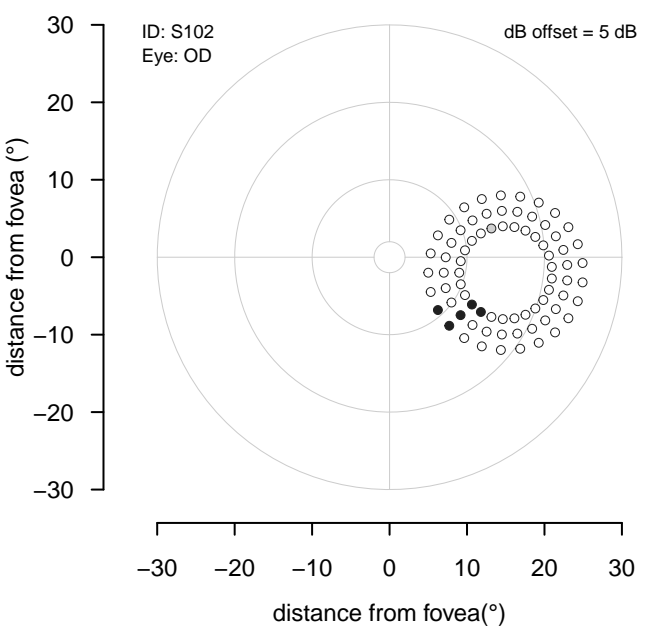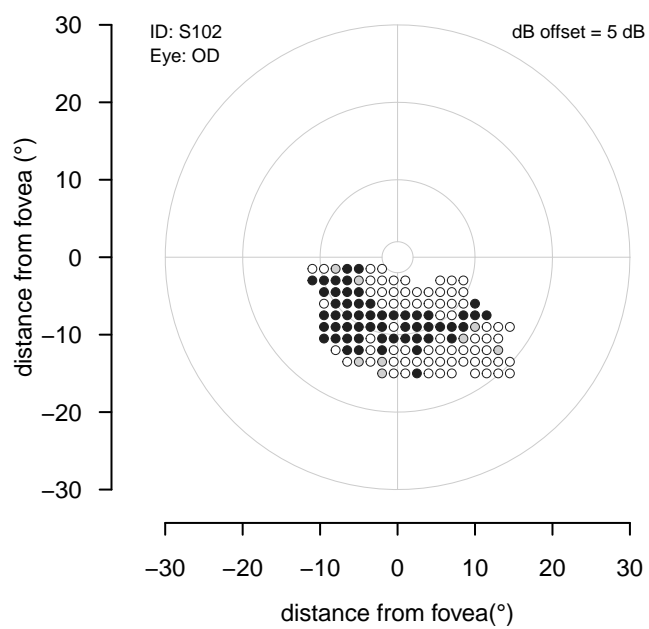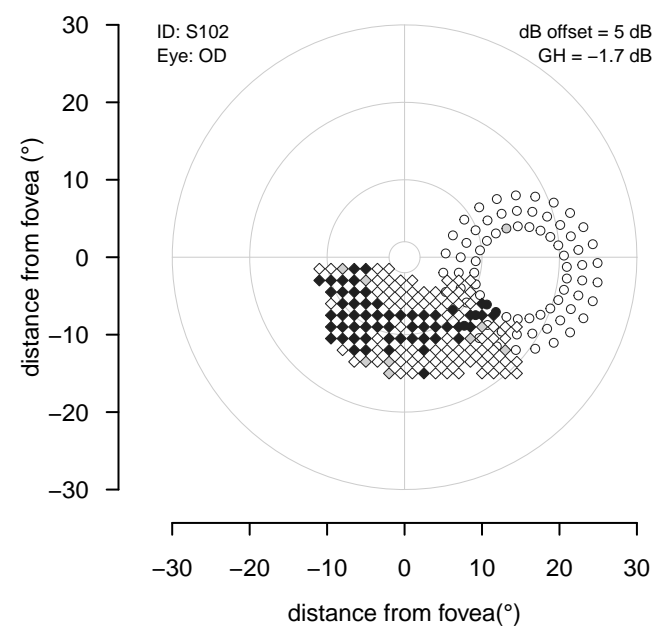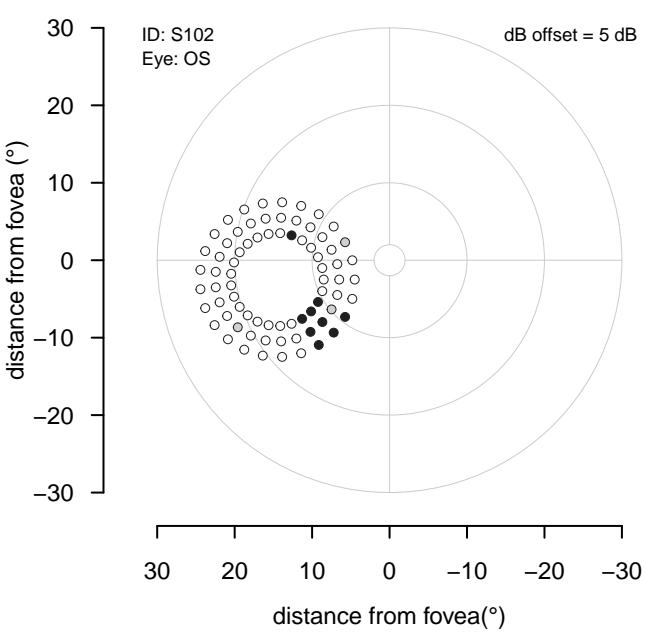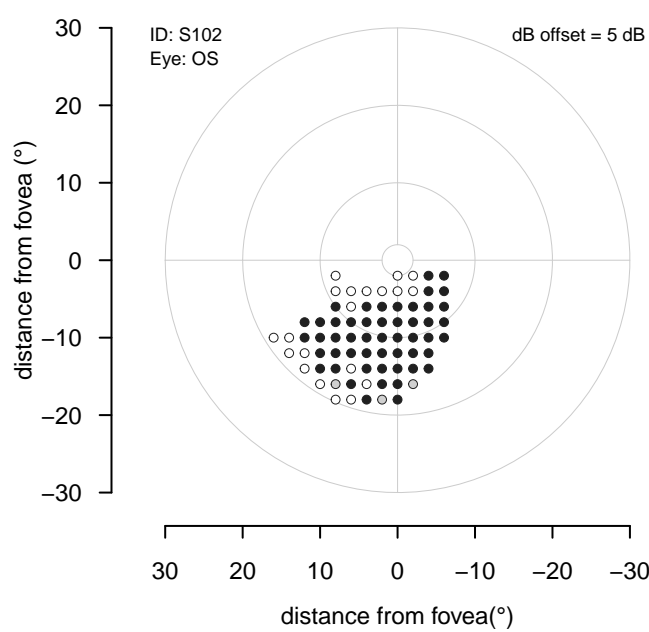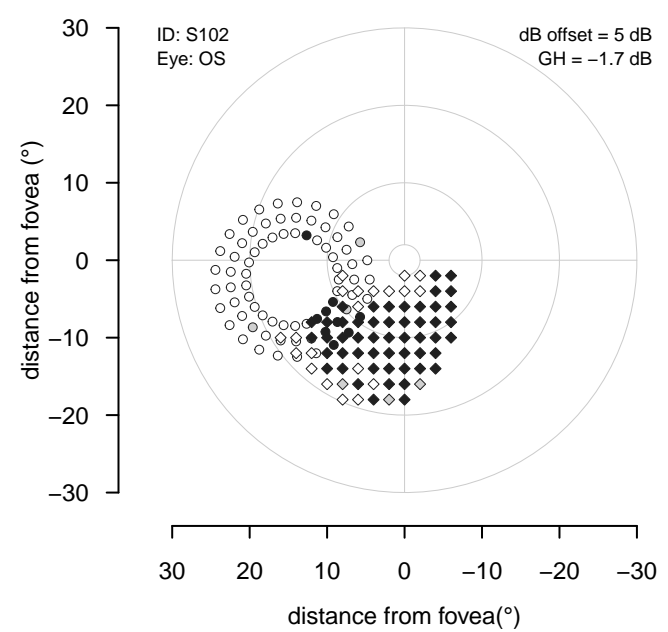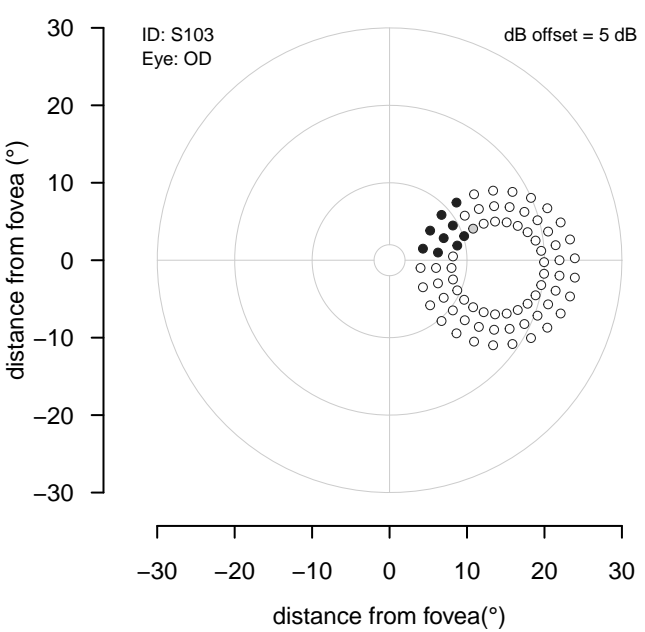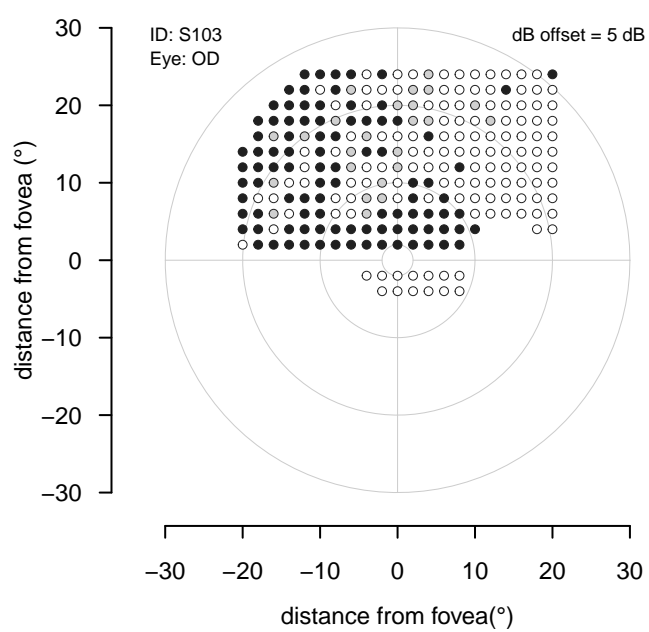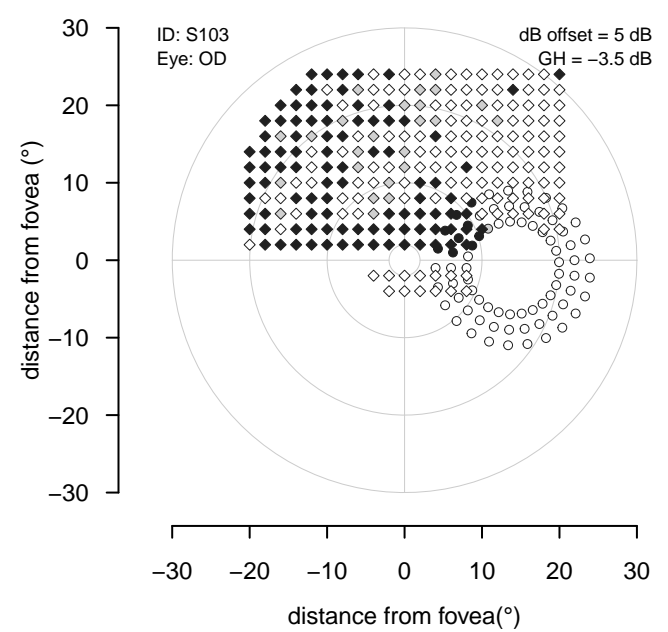

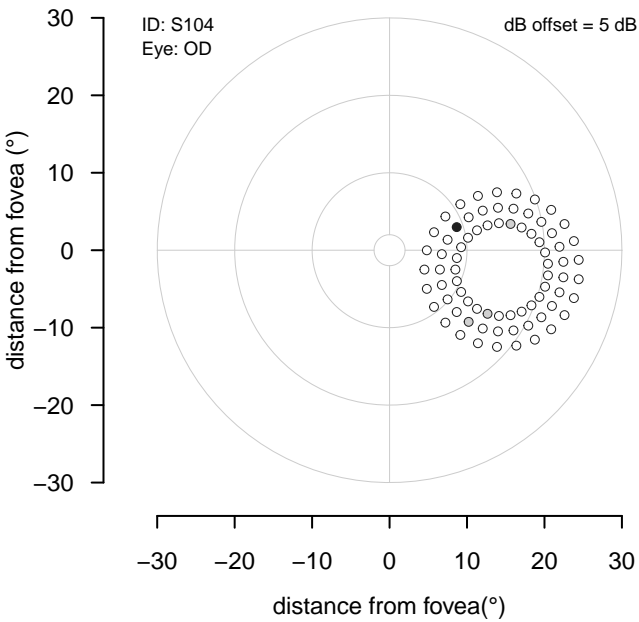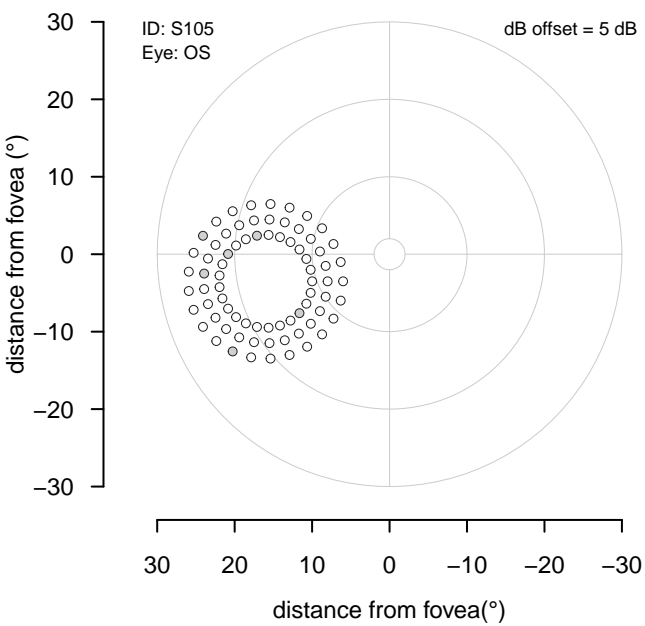

Supplement: Supplementary file 1 — Appendix S1. (ZIP 3.30 MB) [file 44402_2025_4507042_MOESM1_ESM.zip › opo70014-sup-0006-Supinfo6.pdf]
